# Identification of fasciclin-like arabinogalactan proteins in textile hemp (*Cannabis sativa* L.): *in silico* analyses and gene expression patterns in different tissues

**DOI:** 10.1186/s12864-017-3970-5

**Published:** 2017-09-20

**Authors:** Gea Guerriero, Lauralie Mangeot-Peter, Sylvain Legay, Marc Behr, Stanley Lutts, Khawar Sohail Siddiqui, Jean-Francois Hausman

**Affiliations:** 1 Environmental Research and Innovation (ERIN) Department, Luxembourg Institute of Science and Technology (LIST), 5, Avenue des Hauts-Fourneaux, L-4362 Esch/Alzette, Luxembourg; 2 Present address: Institut National de la Recherche Agronomique, Université de Lorraine, UMR 1136, Interactions Arbres-Microorganismes, Champenoux, France; 3 Groupe de Recherche en Physiologie Végétale, Earth and Life Institute-Agronomy, Université catholique de Louvain, Louvain-la-Neuve, Belgium; 4 Life Sciences Department, King Fahd University of Petroleum and Minerals (KFUPM), Dhahran, Saudi Arabia

**Keywords:** Fasciclin-like arabinogalactan proteins, Bast fibres, Cell wall, RT-qPCR, *Cannabis sativa*

## Abstract

**Background:**

The fasciclin-like arabinogalactan proteins (FLAs) belong to the arabinogalactan protein (AGP) superfamily and are known to play different physiological roles in plants. This class of proteins was shown to participate in plant growth, development, defense against abiotic stresses and, notably, cell wall biosynthesis. Although some studies are available on the characterization of *FLA* genes from different species, both woody and herbaceous, no detailed information is available on the *FLA* family of textile hemp (*Cannabis sativa* L.), an economically important fibre crop.

**Results:**

By searching the *Cannabis* genome and EST databases, 23 *CsaFLA*s have been here identified which are divided into four phylogenetic groups. A real-time qPCR analysis performed on stem tissues (isolated bast fibres and shivs sampled at three heights), hypocotyls (6-9-12-15-17-20 days-old), whole seedlings, roots, leaves and female/male flowers of the monoecious fibre variety Santhica 27, indicates that the identified *FLA* genes are differentially expressed. Interestingly, some hemp *FLA*s are expressed during early phases of fibre growth (elongation), while others are more expressed in the middle and base of the stem and thus potentially involved in secondary cell wall formation (fibre thickening). The bioinformatic analysis of the promoter regions shows that the *FLA*s upregulated in the younger regions of the stem share a conserved motif related to flowering control and regulation of photoperiod perception. The promoters of the *FLA* genes expressed at higher levels in the older stem regions, instead, share a motif putatively recognized by MYB3, a transcriptional repressor belonging to the MYB family subgroup S4.

**Conclusions:**

These results point to the existence of a transcriptional network fine-tuning the expression of FLA genes in the older and younger regions of the stem, as well as in the bast fibres/shivs of textile hemp. In summary, our study paves the way for future analyses on the biological functions of FLAs in an industrially relevant fibre crop.

**Electronic supplementary material:**

The online version of this article (doi:10.1186/s12864-017-3970-5) contains supplementary material, which is available to authorized users.

## Background

Arabinogalactan proteins (AGPs) are cell surface glycoproteins belonging to the hydroxyproline-rich glycoprotein superfamily ([1] and references therein) which are involved in many aspects of plant development, i.e. pattern formation, phytohormone interaction, tissue differentiation, reproduction, response to (a)biotic stresses, cell expansion and secondary cell wall deposition [2, 3]. These heavily glycosylated proteins are subdivided into four main classes: classical AGPs, AG peptides, Lys-rich AGPs, fasciclin-like AGPs (FLAs) [3–6].

FLAs are characterized by the occurrence of one or two AGP domains, as well as one or two fasciclin (FAS) domains [7]. FAS domains were first identified in the fruit fly *Drosophila melanogaster* and later found in many other organisms, from bacteria to higher plants to animals [5]. Although a consensus sequence for the FAS domains is lacking, two regions are highly conserved, named H1 and H2 (of ca. 10 amino acids) [5]. Additionally, most FLAs show an N-terminal signal peptide and a C-terminal glycosylphosphatidylinositol (GPI) membrane anchor [5, 7], mediating attachment to the cell surface.

*FLA*s constitute multigene families in plants: for example, 21 *FLA*s have been identified in thale cress, 24 in rice, 35 in poplar, 34 in wheat, 19 in cotton, 33 in chinese cabbage and 18 in eucalypt [5, 7–11]. Molecular studies focused on *FLA*s are important, since they increase our understanding of the molecular functions of this protein family: the available literature on the topic has shown that FLAs in plants are not only related to tissue-specific functions, but also involved in generalized responses to environmental constraints, both biotic and abiotic [3, 7, 11, 12].

Additionally, a strong body of evidence in the literature has highlighted the importance of FLAs in regulating aspects linked to cell wall biosynthesis and, more generally, to stem mechanics in both herbaceous and woody species, as well as fibre growth. For instance, in *Arabidopsis*, insertional mutants of *Atfla11* and *Atfla12* and *Atfla11*/*fla12* double mutants show modified stem mechanics, due to a decrease in cellulose, arabinose and galactose in secondary cell walls [12]. Likewise, in *Eucalyptus*, FLAs belonging to the subgroup A [5, 12] are involved in stem mechanics [11]: in particular *EgrFLA2* is linked to cellulose microfibril angle. In poplar, antisense expression of *PtFLA6* alters secondary cell wall composition in the xylem, by affecting the biosynthesis of lignin and cellulose [13]. In cotton, *GhFLA1* is involved in fibre initiation and elongation: its overexpression increases fibre length, while its silencing results in shorter fibres with an altered primary cell wall composition [14]. In the fibre crop flax, some *FLA*s were shown to be upregulated at the snap point, a physical region marking the transition from elongation to cell wall thickening, hence confirming the potential function of these genes in the regulation of fibre development [15, 16].

**Fig. 1:**
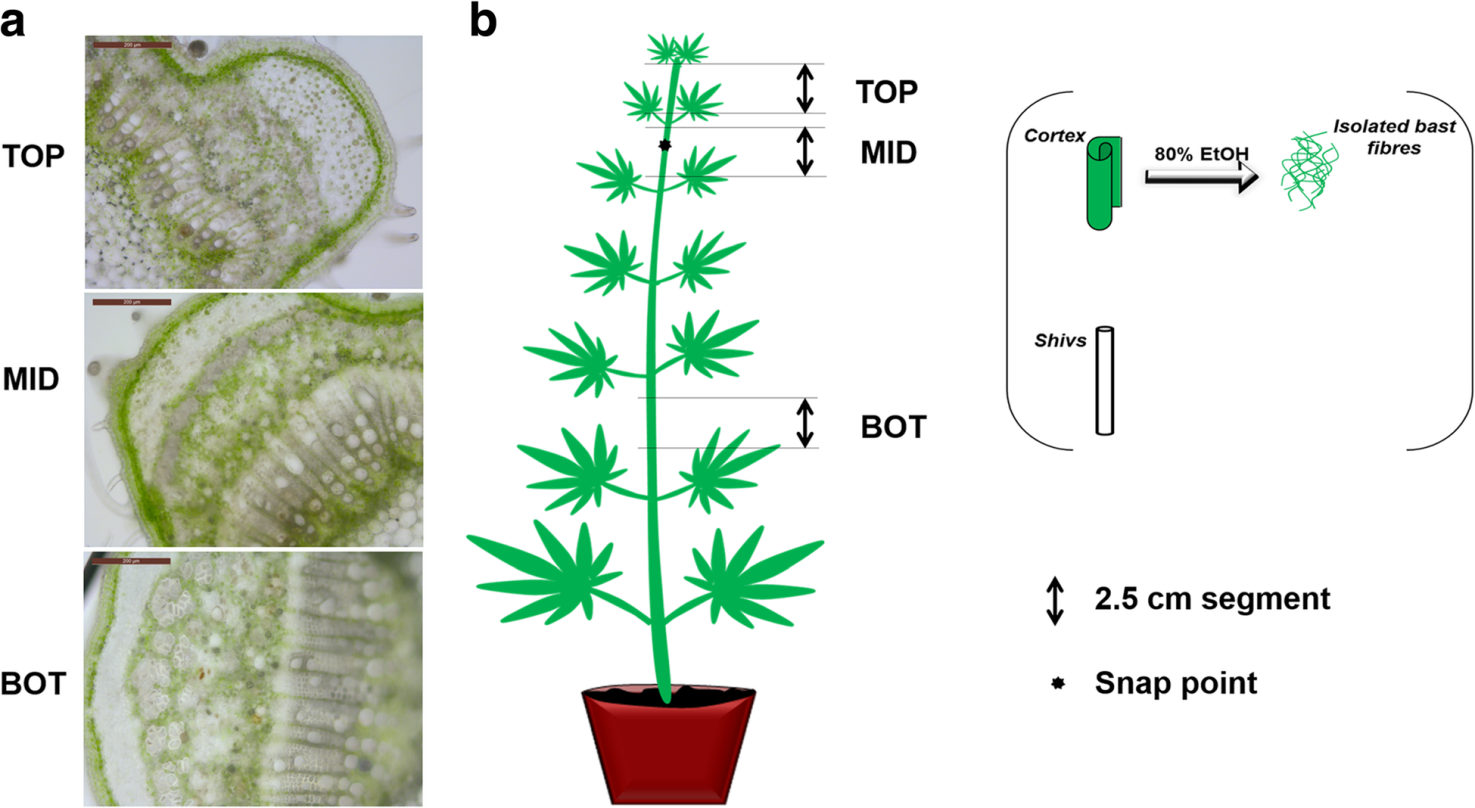
Sampling strategy of the stem tissues. **a** Stem cross-sections of the sampled internodes showing the progressive thickening of bast fibres and the development of the xylem tissue. **b** The segments excised from the sampled internodes are depicted, together with the separated fibres and shivs

Textile hemp (*Cannabis sativa* L.) is an economically important bast fibre-producing crop, with several applications in industry, namely the biocomposite, textile, construction sector [17]. This plant is not only important as a multi-purpose crop, but also useful for fundamental studies centered on cell wall biosynthesis/remodeling [18], because its stem tissues show strong differences in cell types and cell wall composition [19, 20]. The core of hemp stems (a.k.a hurd/shiv) is indeed woody, while the cortex harbors long gelatinous fibres, the bast fibres, with a high content in crystalline cellulose and poor in lignin [21]. The different stem heights correspond to distinctive stages of bast fibre development (from intrusive growth to thickening; Fig. 1a). It is hence possible to study the mechanisms involved in the development of cellulosic and woody fibres by separating the stem tissues of the same plant. The cortex can be peeled from the hurds and the bast fibres can be separated from the surrounding parenchymatic cells with the use of 80% ethanol, a mortar and a pestle [20, 22, 23].

The molecular steps involved in the regulation of bast fibre initiation, development and intrusive growth comprise many still unexplored aspects [24–26]; hence an increased knowledge in these mechanisms would favor the development of biotechnological tools focused on bast fibre improvement.

In the light of the above-mentioned relationships between *FLA*s and cell wall-related processes and considering the industrial applications of *C. sativa*, we here sought to identify and study the expression patterns of hemp *FLA* genes in the different stem tissues, as well as in other organs. By using bioinformatics coupled to RT-qPCR, we show that some *FLA* genes are highly expressed in bast fibres. Moreover, we identify groups of *FLA*s, upregulated either at the top or the bottom of the stem, which share putative conserved elements in their promoters. Our study therefore lays the foundation to further molecular analyses on a unique family of proteins in an important herbaceous crop.

## Methods

### Plant material and growth conditions

A hemp monoecious fibre variety (*C. sativa* cv. Santhica 27) was studied in this work. Plants were grown and sampled as described in [20]. Briefly, after six weeks of growth in controlled chambers, samples were taken along three stem regions localized at different heights with respect to the “snap point” (e.g an empirically-defined reference region marking the transition from elongation to secondary cell wall thickening; [27]). The “TOP” segment internode corresponds to the region right below the apex (above the snap point), the “MID” (middle) segment is the internode containing the snap point and the “BOT” (bottom) segment is located two internodes below the “MID” sample (for clarity, a cartoon depicting the sampling strategy is shown in Fig. 1b). A segment of 2.5 cm was collected in the middle of each internode to avoid too much variation in gene expression, due to the varying developmental stages of the cell types.

Fibres were separated from the shivs by peeling the cortical tissues and by quickly processing them as described in [23]. The shivs were directly plunged in liquid nitrogen and stored at −80 °C. The number of independent biological replicates is four, with the exception of the BOT core tissues, for which the biological replicates are three. A total of 13 plants were pooled for each replicate.

Two leaves (sampled below the TOP region from 4 biological replicates, each composed of a pool of 16 plants) were frozen in liquid nitrogen after removal of the midrib with a scalpel and subsequently stored at −80 °C. Hemp seedlings were obtained by germinating the seeds for 2 days at 25 °C (16 h 25 °C/8 h 20 °C light/dark cycles) on moist cotton wool; four biological replicates, each composed of 15–20 seedlings were frozen in liquid nitrogen and stored at −80 °C until RNA extraction. A pool of 4–5 female and male flowers sampled from 4 biological replicates, each composed of a pool of 5 plants (grown at 60% humidity with a 10 h light 25 °C/14 h dark 20 °C cycle during 5 weeks) were sampled, immediately plunged in liquid nitrogen and stored at −80 °C. Roots from four biological replicates, each composed of 16 plants, were extensively rinsed with tap water to remove soil particles, then blotted dry, directly frozen in liquid nitrogen and stored at −80 °C.

The hypocotyls, aged from 6 to 20 days after sowing, were grown and sampled as described in [18]. Three biological replicates, each consisting of a pool of 20 hypocotyls, were used.

### Identification of *CsaFLA* genes using bioinformatics

In order to identify the *FLA* genes in *C. sativa* (hereafter referred to as *CsaFLA*s for the genes and CsaFLAs for the corresponding proteins), different databases were searched: the Medicinal Plant Genomics Resource (http://medicinalplantgenomics.msu.edu/mpgr_external_blast.shtml) and the *Cannabis sativa* Genome Browser Gateway (http://genome.ccbr.utoronto.ca/cgi-bin/hgBlat?command=start&org=C.+sativa&db=finola1&hgsid=93256). *CsaFLA*s were identified by using orthologous FLA protein sequences of *Arabidopsis thaliana* [5] and *Populus trichocarpa* [7]*.* These sequences were used to perform a BLAT analysis against the hemp Finola and Purple Kush database (*Cannabis* Genome Browser Gateway; [28]) and a BLASTP in the MPGR database. Several incomplete sequences were retrieved when using the MPGR database; however it was possible to deduce their full length sequences either by querying the *Cannabis* Genome Browser Gateway, or the EST database at NCBI (dbEST; available at http://www.ncbi.nlm.nih.gov/dbEST/).

### *In silico* and phylogenetic analyses of *CsaFLA* protein sequences

Putative FAS domains were identified with the Motif Scan algorithm (http://myhits.isb-sib.ch/cgi-bin/motif_scan), N-terminal signal peptides were identified with SignalP (http://www.cbs.dtu.dk/services/SignalP/) and SignalBlast (http://sigpep.services.came.sbg.ac.at/signalblast.html); the subcellular localization was predicted with TargetP (http://www.cbs.dtu.dk/services/TargetP/).

The big-PI Plant Predictor program ([29]; available at http://mendel.imp.ac.at/gpi/plant_server.html) was used to identify the glycosylphosphatidylinositol (GPI) anchor. The 3D homology models of the hemp FLA 10 and FLA 11 were generated with iTASSER Suite ([30] using 4ut1 and 1o70 as targets respectively; available at http://zhanglab.ccmb.med.umich.edu/I-TASSER/) employing LOMETS, SPICKER and TM-align. The models were then refined using REMO by optimizing the backbone hydrogen-bonding networks and FG-MD by removing the steric clashes and improving the torsion angles. The H1 and H2 conserved regions, motifs and residues implicated in adhesion in both proteins were manually annotated according to Johnson et al. [5]. The final structures showing various domains, conserved regions, motifs and residues involved in adhesion were visualized with Swiss PDB Viewer v4.1 [31]. Conserved motifs in the *CsaFLA* promoter sequences (retrieved at the *Cannabis sativa* Genome Browser Gateway) were identified using the MEME Suite 4.11.2 ([32]; available at http://meme-suite.org/doc/cite.html?man_type=web). The identified motifs were subsequently analyzed with Tomtom ([33]; available at http://meme-suite.org/tools/tomtom) for a comparison against the available motifs in the JASPAR CORE plant database 2016 [34]. For the phylogenetic analysis, full-length sequences were aligned with ClustalOmega (http://www.ebi.ac.uk/Tools/msa/clustalo) and the generated alignment submitted to PHYML (http://www.phylogeny.fr) to obtain a maximum likelihood phylogenetic tree. The Maximum Likelihood tree was constructed using an aLRT (approximate likelihood ratio test) for non-parametric branch support, based on a Shimodaira-Hasegawa-like procedure. The tree was visualized with iTOL-Interactive Tree Of Life (http://itol.embl.de/). Intron-exon junctions were visualized with Gene Structure Display Server 2.0 (GSDS, http://gsds.cbi.pku.edu.cn/) [35].

### Immunohistochemistry

Immunohistochemical analyses were performed on resin-embedded tissue sections, as previously described [18]. The LM14 antibody (PlantProbes) was diluted 1:10 in milk protein (MP)/PBS (5% *w*/*v*). Sections were incubated for 1.5 h, rinsed three times in PBS and subsequently incubated for 1.5 h with the anti-rat IgG coupled to FITC (Sigma) diluted 100-fold in MP/PBS.

### RNA extraction and RT-qPCR

Total RNA was extracted using a modified CTAB extraction protocol combined with an RNeasy Plant Mini Kit (Qiagen) according to [23]. The RNA concentration and quality were measured by using a Nanodrop ND-1000 (Thermo Scientific) and a 2100 Bioanalyzer (Agilent), respectively. One microgram of RNA was retrotranscribed into cDNA using the ProtoScript II RTase (NEB) and random primers, according to the manufacturer’s instructions.

The cDNA was diluted to 2 ng/μL and 2 μl used for the RT-qPCR analysis in 384-wells microplates. An automated liquid handling robot (epMotion 5073) was used to prepare the 384-wells microplates (10 μl final volume). A tissue maximization design was used to prepare the microplates [36]. The expression of each *CsaFLA* was normalized using 5 reference genes (tubulin, CDPK, RAN, clathrin and F-box, which geNORM^PLUS^ identified as sufficient for appropriate data normalization) for the stem tissues, as described in [20], and 3 (RAN, TIP41 and F-box) for the other tissues (leaves, seedlings, flowers and roots). For statistical analysis, the normalized relative quantities exported from qBase^PLUS^ were log2 transformed. A one-way ANOVA was carried out using IBM SPSS Statistics v19. A Tukey’s HSD was performed as post-hoc test. The normal distribution of the data was verified with a Kolmogorov–Smirnov test.

### Primer design

Primers were designed using Primer3Plus (http://www.bioinformatics.nl/cgi-bin/primer3plus/primer3plus.cgi/) and verified with the OligoAnalyzer 3.1 tool from Integrated DNA technologies (http://eu.idtdna.com/calc/analyzer). Primer efficiencies were checked via qPCR using a serial five-fold dilution of cDNA (25, 5, 1, 0.2, 0.04, 0.008 ng/μL). The primer sequences, amplicon length and T_m_, amplification efficiencies and R^2^ are indicated in Additional file 1: Table S1.

### Sequencing of some representative *CsaFLA* promoters

To determine the homology of the promoter sequences of the variety Santhica 27 with those from the PurpleKush and Finola reference genomes, primers were designed on 3 representative genes (*CsaFLA2*–7-*16*) using the available sequences at the *Cannabis sativa* Genome Browser to perform nested PCRs (Additional file 2: Table S2). Genomic DNA was extracted from stem tissues (whole internodes) by using a CTAB-based protocol coupled to the NucleoSpin Plant II kit (Macherey-Nagel). Briefly, 500 μl of extraction buffer (2% CTAB, 2.5% PVP-40, 2 M NaCl, 100 mM Tris-HCl pH 8.0, 25 mM EDTA and 10 μl RNase) were added to 100 mg of finely ground sample and the slurry was vortexed vigorously. After an incubation step at 60 °C for 10 min, 20 μl β-ME/ml buffer were added and the samples were further incubated for 20 min at 60 °C. Subsequently, 500 μl chloroform/isoamyl alcohol 24:1 were added, the samples were vortexed and centrifuged at RT for 10 min at 10000 g. To the aqueous phase, 2/3 cold isopropanol were added and the DNA was precipitated for 1 h at −20 °C. After this stage, the Nucleospin II columns were used to bind the DNA and the manufacturer’s instructions were followed to elute genomic DNA.

PCRs were performed using 50 ng DNA and the Q5 Hot Start High-Fidelity 2X Master Mix, following the manufacturer’s instructions. The optimal annealing temperatures were computed using the NEB T_m_ calculator (available at http://tmcalculator.neb.com/#!/).

PCR products were ligated into the pGEM-T Easy vector, following the manufacturer’s instructions and cloned into JM109 chemically competent cells. Three positive clones for each gene promoter were grown o/n at 37 °C in LB medium supplemented with ampicillin 100 μg/ml. Plasmids were extracted using the QIAGEN plasmid miniprep kit and sequenced on an Applied Biosystems 3500 Genetic Analyser using the BigDye Terminator v3.1 Cycle Sequencing and the BigDye XTerminator Purification kits, according to the manufacturer’s instructions.

## Results

### Identification of putative *FLA*s in *C. sativa*: Protein architecture and phylogenetic analysis

BLAST/BLAT analyses of the 21 *A. thaliana* sequences (*AtFLAs*) performed against the Medicinal Plant Genomics Resource, the NCBI EST and the *Cannabis* Genome Browser Gateway databases led to the identification of 23 *CsaFLA*s (Additional file 3: Table S3). It should be noted that, during the database queries, a contig, i.e. csa_locus_44222_iso_1_len_407_ver_2, which was initially called *CsaFLA22* and retrieved at the Medicinal Plant Genomics Resource (MPGR), was also found. However, we believe that this partial gene was erroneously attributed to *C. sativa*, since we never amplified any product with different primers designed on it and the reported FPKM values at the MPGR are 0 for all the tissues examined. We discarded this gene from our analyses, but kept the original nomenclature given to the hemp FLA genes (i.e. *CsaFLA1*–*24*), as at this stage we cannot rule out the existence of this gene in textile hemp.

The intron-exon structure analysis highlighted the presence of 5 genes (*CsaFLA2*-*17*-*8*-*12*-*4*) containing one intron: of these genes, *CsaFLA12* and *4*, both possessing a small intron, group together according to the maximum likelihood (ML) phylogenetic tree, while *CsaFLA2*–*17*–*8*, containing longer introns, are in a different branch (Additional file 4: Figure S1). To check that the putative *CsaFLA*s belong to the FLA family, the occurrence of the following features was checked: the presence of at least one FAS domain, a signal peptide at the N-terminus, (in some cases) a GPI anchor at the C-terminus and the presence of AGP domains (Additional file 5: Table S4; Additional file 6: Figure S2). The identified FLAs show a PAST% ranging from 19.4 to 44.5% (Table 1), which is in agreement with the values reported for poplar FLAs [7].

**Table 1:** Proline, Alanine, Serine, and Threonine (PAST) proportions in CsaFLAs

FLA	Proline%	Alanine%	Serine%	Threonine%	PAST%	Class
CsaFLA1	5.7	13.8	6	7.6	33.1	C
CsaFLA2	3.6	7.2	9.3	9.3	29.4	C
CsaFLA3	8	9.9	9.9	6.9	34.7	A
CsaFLA4	8.2	9.8	12.8	8	38.8	C
CsaFLA5	14.2	4.7	13.9	6.4	39.2	B
CsaFLA6	7.9	8.8	9.6	7.9	34.2	A
CsaFLA7	9.6	10.3	11.9	8.4	40.2	A
CsaFLA8	8.3	6.6	7.5	5.8	28.2	B
CsaFLA9	8.4	7.3	10.2	10.2	36.1	A
CsaFLA10	8.9	11.2	11.8	9.4	41.3	C
CsaFLA11	6.4	9.2	11.2	9.6	36.4	A
CsaFLA12	8.3	8	16	11.5	43.8	A
CsaFLA13	12.6	8.4	12.3	10.2	43.5	A
CsaFLA14	8.7	15.5	12.9	7.4	44.5	C
CsaFLA15	8.6	11.7	11.7	10.5	42.5	A
CsaFLA16	8.2	10.4	14.6	8.8	42	A
CsaFLA17	8.2	6.9	7.8	5	27.9	B
CsaFLA18	8.8	8.2	9.8	8.5	35.3	A
CsaFLA19	8.2	8.2	15.2	11.7	43.3	A
CsaFLA20	6.4	7.6	10.9	5.8	30.7	D
CsaFLA21	6.5	7.3	10.1	7.6	31.5	D
CsaFLA23	7.7	6.5	13.6	9.3	37.1	D
CsaFLA24	2.5	3	10.4	3.5	19.4	D

The classes for each target are also indicated

**Fig. 2:**
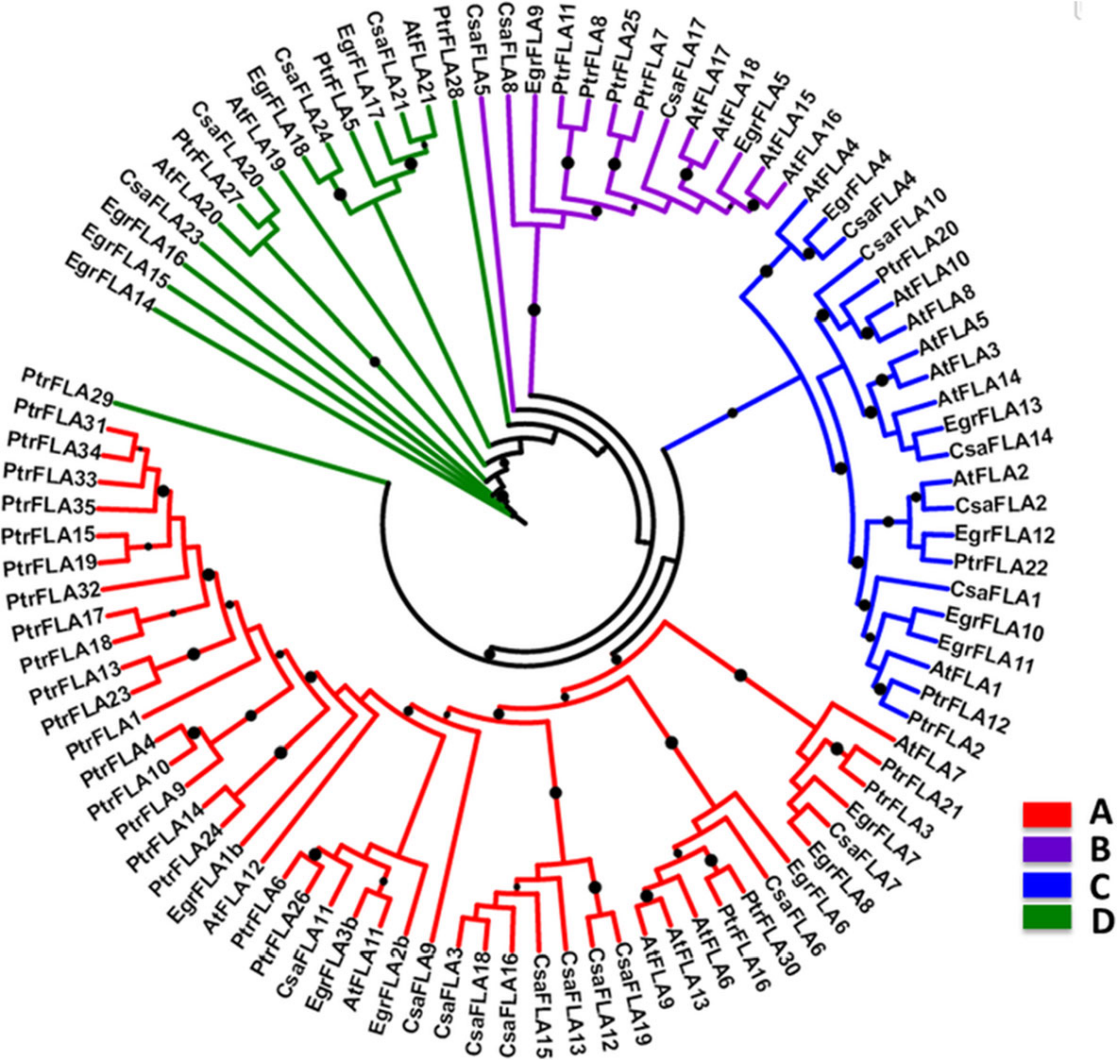
The phylogenetic tree of FLA proteins from hemp, poplar, eucalypt and thale cress. The maximum likelihood tree was constructed with 23 FLA proteins in hemp, 18 in eucalypt [11], 35 in poplar [7] and 21 in *Arabidopsis *[5]. Only bootstrap values higher than 0.8 are here visualized as black circles (the bigger the circle, the higher the bootstrap value)

In order to investigate the evolutionary relationship between known plant FLA proteins, a maximum-likelihood phylogenetic tree was built using the identified 23 CsaFLAs, 21 AtFLAs, 18 *Eucalyptus* (EgrFLAs) and 35 poplar sequences (PtrFLAs) (Fig. 2 and Additional file 6: Figure S2). It should be noted that we chose to perform the phylogenetic analysis using full-length CsaFLA sequences to conform to the previously published tree of poplar FLAs [7]. Different results may be obtained if the mature protein sequences are used.

CsaFLAs cluster into four major classes (A-D). Class A is the largest clade, with 11 hemp members (CsaFLA3/6/7/9/11/12/13/15/16/18/19) containing a single FAS domain flanked by two AGP domain and a GPI anchor at the C-terminus. Class B includes CsaFLA5/8/17 which contains two FAS domains and a single AGP region. Class C comprises 5 members (CsaFLA1/2/4/10/14), characterized by two FAS, two AGP domains and a GPI anchor. The last class includes 4 proteins (CsaFLA20/21/23/24) with no distinctive protein architecture (although CsaFLA20 and CsaFLA23 have 2 FAS domains like group B, however their lengths are smaller). The percentage of CsaFLA identity with the putative orthologs from thale cress is between 32 and 76% (Table 2).

**Table 2:** Identities of CsaFLAs with AtFLAs

Name	Class	*Arabidopsis* sequence identity	*Arabidopsis* sequence identity
CsaFLA1	Class C	AtFLA1 AT5G55730	63%
CsaFLA2	Class C	AtFLA2 AT4G12730	60%
CsaFLA3	Class A	AtFLA11 AT5G03170	49%
CsaFLA4	Class C	AtFLA4 AT3G46550	56%
CsaFLA5	Class B	AtFLA19 AT1G15190	32%
CsaFLA6	Class A	AtFLA9 AT1G03870	54%
CsaFLA7	Class A	AtFLA7 AT2G04780	62%
CsaFLA8	Class B	AtFLA17 AT5G06390	71%
CsaFLA9	Class A	AtFLA11 AT5G03170	57%
CsaFLA10	Class C	AtFLA10 AT3G60900	72%
CsaFLA11	Class A	AtFLA11 AT5G03170	64%
CsaFLA12	Class A	AtFLA12 AT5G60490	55%
CsaFLA13	Class A	AtFLA13 AT5G44130	42%
CsaFLA14	Class C	AtFLA14 AT3G12660	45%
CsaFLA15	Class A	AtFLA11 AT5G03170	53%
CsaFLA16	Class A	AtFLA12 AT5G60490	55%
CsaFLA17	Class B	AtFLA17 AT5G06390	76%
CsaFLA18	Class A	AtFLA11 AT5G03170	56%
CsaFLA19	Class A	AtFLA11 AT5G03170	55%
CsaFLA20	Class D	AtFLA20 AT5G40940	36%
CsaFLA21	Class D	AtFLA21 AT5G06920	45%
CsaFLA23	Class D	AtFLA20 AT5G40940	36%
CsaFLA24	Class D	No homology	

Details concerning the class and orthologous *Arabidopsis* FLAs are given, together with the identity percentage

### *CsaFLA* expression patterns in hemp tissues

An immunohistochemical analysis carried out with the LM14 antibody (recognizing AGPs) revealed that the epitope is distributed in different tissues of the hemp stem (Additional file 7: Figure S3): this result shows the broad distribution of these proteins in the different hemp stem tissues. In particular, in the bottom internode, AGPs are present in the core tissues (cell walls of fibres and vessels), cortical parenchyma/collenchyma and, notably, in the inner region of the fibres, i.e. the layer (plasma membrane) delimiting the fibre cell lumen.

**Fig. 3:**
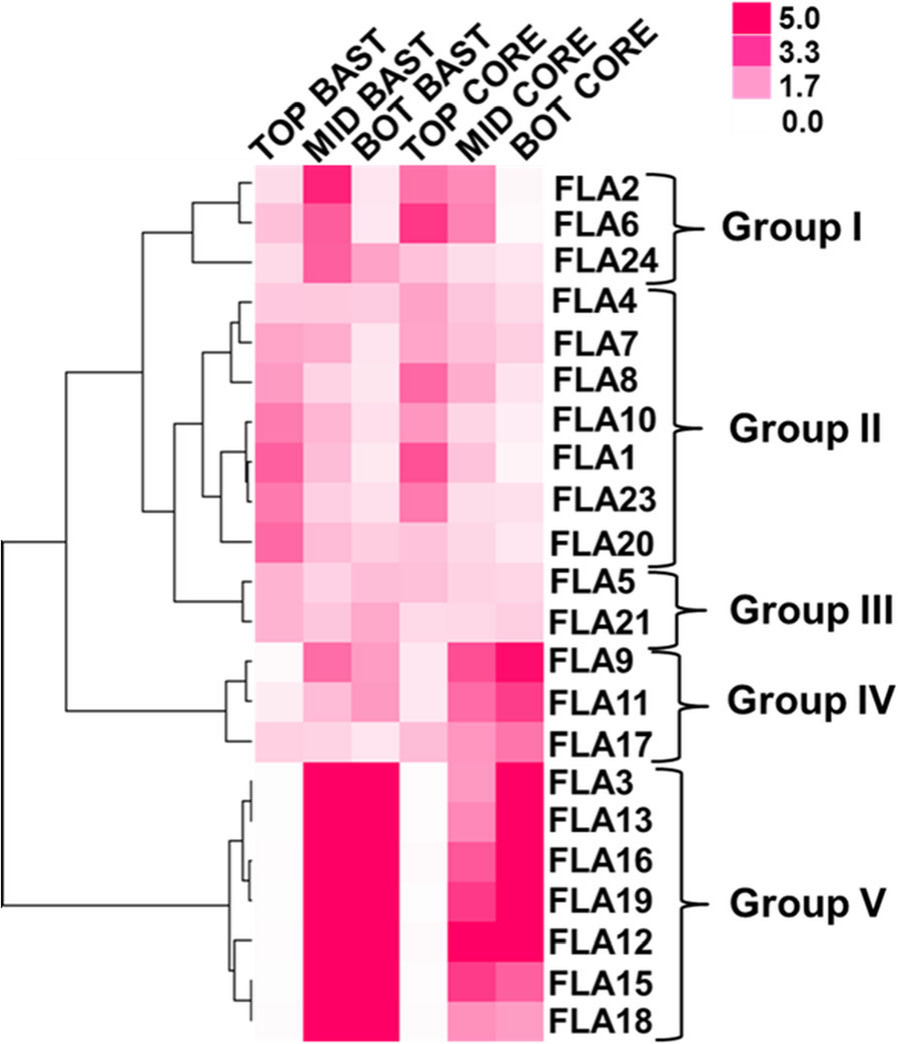
Expression profiles of *CsaFLA*s in hemp stems. Hierarchical clustering of expression profiles of 22 *CsaFLA*s in the three regions of stem. Data in the form of bar graph are shown in Additional file 8: Figure S4

Of the 23 *CsaFLA*s identified, 22 were expressed in the stem tissues (Fig. 3; Additional file 8: Figure S4). *CsaFLA14* was detected at very low levels in the stem tissues (Ct > 32).

In hemp bast fibres, the heat-map hierarchical clustering shows 5 major expression trends (Fig. 3). These are the following: 1) a group of genes (*CsaFLA2*–*6*-*24*) is upregulated at the middle internode containing the snap point (in the core the expression decreases towards the base of the stem); 2) *CsaFLA1*-*4*-*7*-*8*-*10*-*20*-*23* are expressed at higher levels in the top and decreased towards the bottom internode; 3) two *FLA*s, *CsaFLA5* and *21*, are downregulated at the snap point; 4) three genes, *CsaFLA9*-*11*-*17*, show a tendency to upregulation at the snap point, although the pattern is less marked with respect to group I (and in the core the expression increased towards the stem base); 5) the last group comprises *FLA*s upregulated at the bottom (*CsaFLA3*-*12*-*13*-*15*-*16*-*18*-*19*).

**Fig. 4:**
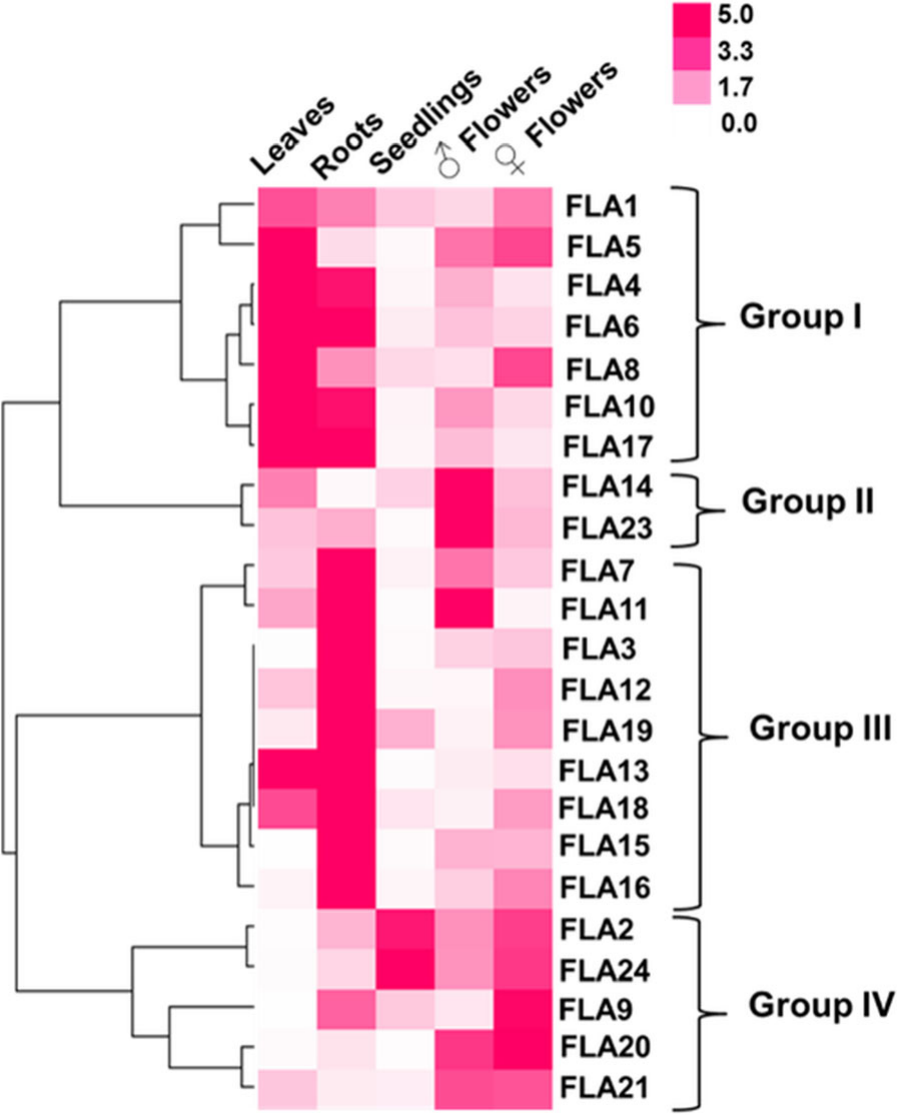
Expression profiles of *CsaFLA*s in the other tissues. Hierarchical clustering of expression profiles of 23 *CsaFLA*s in the leaves, roots, seedlings, male/female flowers. Data in the form of bar graph are shown in Additional file 9: Figure S5

Gene expression analysis was carried out on other hemp tissues, i.e. leaves, roots, male/female flowers and seedlings, to check whether hemp *FLA*s expressed at low levels in the stem showed a distinctive expression pattern in other hemp tissues (Fig. 4; Additional File 9: Figure S5). The heat-map hierarchical clustering reveals the presence of 4 main expression patterns (Fig. 4). More specifically, a group of genes, comprising *CsaFLA1*-*4*-*5*-*6*-*8*-*10*-*17*, is expressed at higher levels in hemp leaves; in group II, two genes specifically upregulated in male flowers are present (*CsaFLA14* and *23*); group III comprises 9 FLAs expressed at higher levels in hemp roots (*CsaFLA3*-*7*-*11*-*12*-*13*-*15*-*16*-*18*-*19*); the fourth group is represented by *CsaFLA2*-*9*-*20*-*21*-*24*, which are overall more expressed in female flowers.

**Fig. 5:**
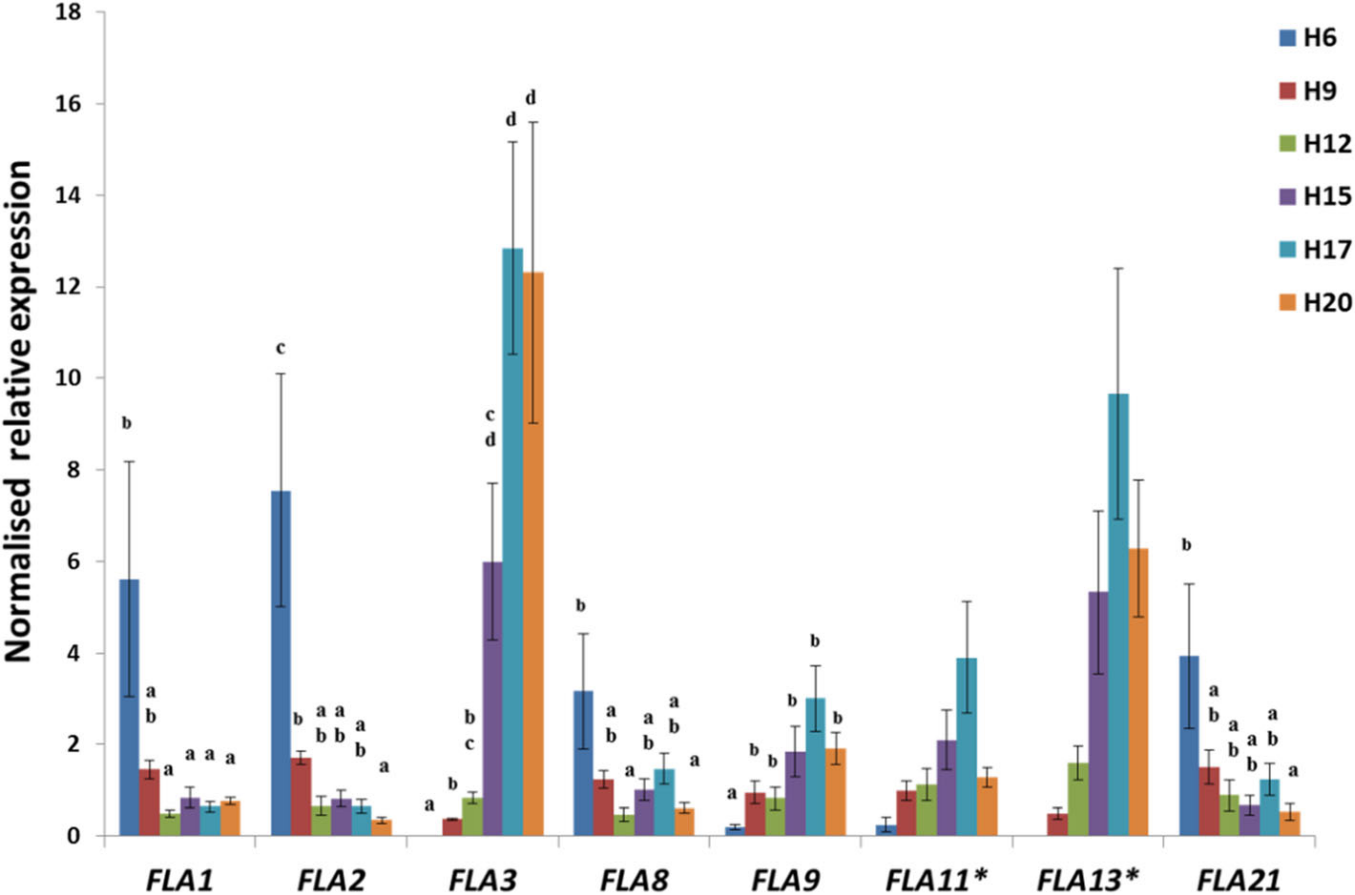
Expression analysis of 8 *CsaFLA*s in the hypocotyls of *C. sativa*. Error bars indicate the standard error of the mean (*n* = 3). Different letters indicate statistically significant values at the one-way ANOVA test (*p* < 0.05) with a Tukey post-hoc test. * indicates a non-normal distribution (Kolmogorov-Smirnov test). H6-H9-H12-H15-H17-H20 indicate hypocotyls aged 6–9–12--15–17–20 days

The expression of some *FLAs* belonging to the above-mentioned 5 stem groups has also been investigated in hypocotyls, aged 6-9-12-15-17-20 (H6 to H20). The hemp hypocotyl was proven to be a suitable model to study cell wall-related processes accompanying secondary growth [18], therefore the goal was to verify whether their expression pattern highlighted the same trend observed in adult stems. *CsaFLA1* **-**2**-**8**-**21 were more expressed in young hypocotyls (H6); *CsaFLA3* **-**9**-**11**-**13 were more expressed in H15, H17 and H20 (Fig. 5).

### Identification of conserved motifs in the promoters of some *CsaFLAs*

A bioinformatic analysis was carried out on the available promoter regions (between 116 and 1064 bp retrieved at the *Cannabis* Genome Browser Gateway) of the *CsaFLA* genes showing a distinctive expression pattern in the bast fibres. The promoters of the genes within group I, II and V were selected (Additional File 10), in the light of their upregulation at the snap point, at the younger and older stem regions respectively (Fig. 3). While no conserved motifs could be found with the MEME suite tool for the genes in group I, 1 motif was found for the genes within group II and V (Table 2). The search carried out with the conserved sequence of group II and V *FLA*s in the JASPAR CORE 2016 plants database identified SOC1 (lowest *p*-value among the matches retrieved) and MYB3 as candidates recognizing similar motifs (Table 3). In addition to that, the promoters of three representative *CsaFLA*s showing upregulation at the snap point, at the top, or at the bottom (*CsaFLA2*, *CsaFLA7* and *CsaFLA16*) were amplified and cloned. The purpose was to verify the sequence conservation between the fibre variety Santhica 27, the Purple Kush strain and Finola variety [28]. The motifs identified by MEME were cross-verified with PlantPAN 2.0 (http://plantpan2.itps.ncku.edu.tw/) and are highlighted in yellow in the promoters of *CsaFLA7* and *CsaFLA16* from Santhica 27 (Additional File 11).

**Table 3:** Conserved motifs in the promoters of *FLA*s from group II and V

*FLA*s	Conserved motif (MEME)	Similar motifs (Tomtom/JASPAR CORE 2016 plants)	Function	*p-*value
Group II		MA0554.1 (SOC1)	Transcription activator controlling flowering time. Probably also involved in photoperiod perception.	5.39e-05
Group V		MA1038.1 (MYB3)	Repression of the phenylpropanoid biosynthesis-related genes. Response to salt stress, wounding, ABA, SA.	1.59e-02

The first motif in each table cell is the one found in the database, the second motif is the one found in the promoters of hemp *FLAs*. The function and computed *p*-value are indicated.

### Domains, conserved regions, motifs and residues mediating adhesion in CsaFLAs from class A and C.

**Fig. 6:**
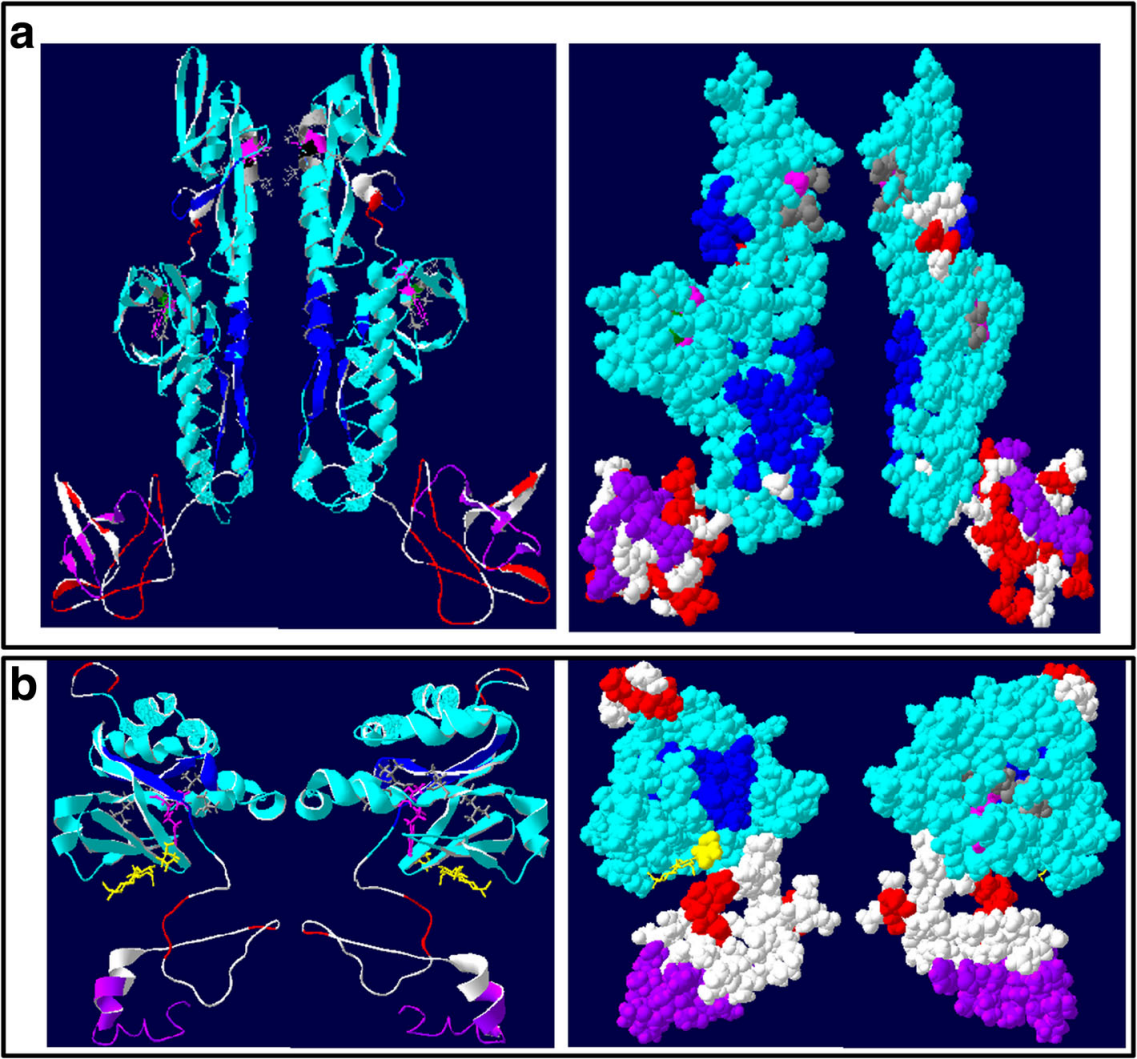
Modeling of representative CsaFLAs. **a** Homology model of FLA10 showing right and left views. *Left*: ribbon model showing secondary structures. *Right*: space-filled model. Both fasciclin (FAS) domains are shown in turquoise and the H1 and H2 conserved regions characteristic of FAS domains within both FAS domains are shown in blue, AGP domains are shown in red and GPI anchors are shown in purple. Conserved aliphatic amino acids (A, L and V, V) on either side of conserved LH motif (pink) and V, L and L on either side of YH motifs (pink) believed to be involved in adhesion are shown in grey whereas polar Ser is shown in green. Residues in white do not belong to any particular domain. **b** Homology model of FLA11 showing right and left views. *Left*: ribbon model showing secondary structures. Right: space-filled model. Fasciclin (FAS) domain is shown in turquoise and the H1 and H2 conserved regions characteristic of FAS domain within a single continuous FAS domain are shown in blue, AGP domains are shown in red and GPI anchors are shown in purple. Conserved aliphatic amino acids (L, V) on either side of conserved YH motif (*pink*) believed to be involved in adhesion are shown in grey. Conserved Asn linked to 2 N-acetyl glucosamine moieties are shown in yellow. Residues in white do not belong to any particular domain

Homology models of one representative each from Class C (FLA 10) and Class A (FLA 11) showing FAS, AGP and GPI anchor domains, H1 and H2 conserved regions, putative amino acids involved in adhesion and [YFL]H motif are given in Fig. 6 and Table 4. The conserved sequences for FLA10 for the first FAS domain are LTVLVLSNGA and ISILEISAPII for H1 and H2 regions respectively and the [YF]H motif is AL-X-LH-VV (Fig. 6a; Table 4). The conserved sequences for FLA10 for the second FAS domain are LTLFAPNDEA and LVIFTVDNVL for H1 and H2 regions respectively and the [YF]H motif is VL-X-YH-SL (Fig. 6a; Table 4). The conserved sequences for FLA11 for a single FAS domain are ITVFAPTDSA and LSVFEVDQVL for H1 and H2 regions respectively and the [YF]H motif is LV-X-YH-VL (Fig. 6b; Table 4).

**Table 4:** Solvent accessibility of residues within conserved motifs

FLA	Conserved motif	Solvent accessibility
CsaFLA10	(AL)-X-LH-(VV) (VL)-X-YH-(SL)	(9,8)-2,4-(4,0) (9,9)-4,3-(4,7)
CsaFLA11	(LV)-X-YH-(VL)	(9,9)-1,6-(6,8)

Residues within brackets are thought to be involved in adhesion. The solvent accessibility of X residue is not given. Solvent accessibility is determined by iTASSER suite and the range is from 0 (completely buried) to 9 (completely solvent exposed)

The two hydrophobic residues preceding and after [YF]H motif are thought to be implicated in mediating adhesion of these proteins. It is interesting to note that these residues are generally aliphatic amino acids such as I, L and V [5], however in FLA10 an Ala and polar Ser are found in the first and second FAS domains respectively (Fig. 6a).

It is noteworthy that [YF]H residues are either completely or partially buried, however both residues that flank [YF]H motif on the N-terminal side (AL, VL, LV) are completely solvent-exposed in both FLA10 and FLA11 (Table 4). In contrast, aliphatic residues (VV) on the C-terminal side of [YF]H motif in H1 domain of FLA10 are either completely or partially buried, whereas polar Ser is partially buried, but Leu is exposed in the H2 domain (Fig. 6a, space-filled). For FLA11 both VL residues on the C-terminal side of [YF]H motif are solvent exposed (Fig. 6b, space-filled). In general, residues belonging to FLA11 and those located towards the N-terminal side are exposed more favorably to mediate adhesion than FLA10 and those located on the C-terminal side. This suggests that adhesion for both these proteins may be mediated via hydrophobic interactions.

## Discussion

The FLAs identified in *C. sativa* group into the previously described four phylogenetic classes (Fig. 2) [5]. A nomenclature of *CsaFLA*s is hereby also proposed which follows the *Arabidopsis* classification (i.e. when the phylogenetic tree highlighted clustering of a CsaFLA proteins with a specific AtFLA, the same number was assigned to *C. sativa*).

Within class A, the largest, it is possible to observe a separate clade represented by *CsaFLA3-12-13-15-16-18-19* which is highly expressed at the snap point and in the older stem regions, both in the bast fibres and the shivs (Fig. 3). A subset of class A genes (*CsaFLA3-9-11-13*) was more expressed in the old hypocotyls (peaking at H17 with high values at H15 and H20). As previously shown [18], the hypocotyl undergoes secondary growth in H9 and later time-points. The phylogenetic position of this cluster of FLAs, together with their common expression pattern, might indicate a specific role in secondary growth. This group of genes may indeed represent hemp-specific single FAS domain FLAs specialized in secondary growth, in a manner analogous to what was previously shown in eucalypt and thale cress [11, 12]. Hemp is unfortunately recalcitrant to transformation, therefore homologous testing, as previously performed on e.g. eucalypt *FLA*s [11], is cumbersome. However heterologous testing in a more amenable system, e.g. *Nicotiana tabacum*, can confirm or refute the hypothesis.

It is here worth discussing also the phylogenetic position of CsaFLA11 in a clade grouping AtFLA11, EgrFLA2b and EgrFLA3b (Fig. 2). These genes were shown to affect stem mechanics, as well as cell wall architecture [11, 12]. The *AtFLA11* transcript was detected in the xylem and interfascicular fibres in inflorescence stem, preceding the lignification of those two tissues [37]; *CsaFLA11* also shows a gradual increase in expression towards the older regions of the stem and it is slightly more expressed in the older hypocotyl too (Fig. 5). This FLA represents another interesting candidate putatively involved in cell wall-related processes in textile hemp.

Within class C, CsaFLA4 and CsaFLA1 group together with the characterized orthologs from thale cress. AtFLA4 (SOS5) is involved in cell expansion [38] and AtFLA1 was shown to regulate root and shoot development in tissue culture [39]. *CsaFLA8* was more expressed in the TOP region of the stem, as well as in H6, suggesting a role in elongating tissue. However, it remains to be shown whether the hemp genes are involved in the same regulatory networks as in *Arabidopsis*.

The expression of the 23 *CsaFLA*s was first investigated in the different tissues of the stem, because we wanted to identify those genes specifically associated with a tissue-type and a stem region. Among them, we would like to draw the reader’s attention on the first group of genes, represented by *CsaFLA2*-*6*-*24*, because they show a different expression profile in the bast fibres and the shivs. The expression in the shivs shows a decrease from the top to the bottom of the stem, while in the bast fibres their expression peaks at the snap point. This is quite interesting if we consider that the snap point is the region marking a shift in the stem mechanical properties, as it determines the transition from cell elongation to thickening [27]. It was shown that the young stem regions of hemp at the vegetative stage of growth are characterized by the presence of ca. 66% glucose, while older regions have about 82%: this result confirms that during their transition from elongation to thickening, bast fibres require great amounts of glucose for the synthesis of cellulose [40]. The 3 FLAs may therefore be involved in cell wall-related processes occurring during this transition. Additionally, this is in agreement with the flax microarray data showing upregulation of certain *FLA*s around the snap point [15] and with the increased expression of poplar *FLA*s in tension wood, which, like bast fibres, is composed of a cellulosic G-layer [41, 42]. As previously discussed for poplar tension wood, specific FLAs with a GPI-anchor might be involved in the cytoskeleton-cell wall connections during fibre expansion/elongation [41]. This would be the case of CsaFLA2 and CsaFLA6, which possess a GPI-anchor (Additional file 5). In the hypocotyl, *CsaFLA2* was significantly more expressed in H6 (Fig. 5). FLAs might also be involved in triggering a cellular signal inducing the formation of the G-layer, via the cleavage of their GlcNAc oligosaccharides by the action of chitinases [22, 41]. It was shown that in flax stems, specific chitinases are highly expressed in bast fibres and may regulate G-layer formation in these cell types [22]. Therefore, it is reasonable to assume that the concerted action of specific FLAs and chitinases may be involved in the transition from elongation to G-layer formation in hemp.

In group II and V are FLAs which, in the bast fibres, show a gradual decrease from the apical to the basal part of the stem and an increase in expression, respectively. A similar trend was observed in the hypocotyls: *CsaFLA8* (belonging to the stem group II) was more expressed in H6; *CsaFLA13* (belonging to the stem group V) was more expressed in H15, H17 and H20 (Fig. 5). In addition, the hypocotyl expression pattern of *CsaFLA3* was similar to the one of *CsaFLA13*. Our study therefore identified specific FLAs likely involved in bast fibre elongation during intrusive growth (*CsaFLA1*-*4*-*7*-*8*-*10*-*20*-*23*) and others involved in secondary cell wall deposition during the thickening stage (*CsaFLA3*-*12*-*13*-*15*-*16*-*18*-*19*).

The expression of hemp FLAs was also investigated in other tissues, notably leaves, roots, male/female flowers and in seedlings (Fig. 4).

The genes belonging to group III in stem tissues (Fig. 3) are highly expressed in roots: within this cluster of *FLA*s are the orthologs of *AtFLA7* and *﻿AtFLA11* (Fig. 2) for which a higher number of ESTs was retrieved in the roots of thale cress [5].

In reproductive organs, the RT-qPCR results show that some genes are highly expressed in male and female flowers. This suggests that some *FLA*s are involved in hemp inflorescence formation.

In seedlings, two genes showed higher expression, i.e. *CsaFLA2* and *24* (Fig. 4). Notably, *CsaFLA2* is the ortholog of *AtFLA2* (Fig. 1), whose EST number is higher in *Arabidopsis* flower buds [5].

In order to investigate whether specific regulatory elements occurred in the promoters of the genes showing specific expression patterns in the stem tissues, we analyzed the genes from group I-II and V (Fig. 3). While for group I no conserved motifs could be obtained, 2 conserved sequences were found for group II and V (Table 2). A conserved motif recognized by the MADS box transcription factor SOC1 could be identified in the promoters of the genes upregulated in the apical stem regions: this finding suggests that they may be involved in a developmental program regulating the transition from vegetative to reproductive growth and/or the response to hormonal regulation (e.g. via gibberellin). In this respect it is noteworthy that in *A. thaliana* SOC1 was shown to control the annual growth habit [43]: *soc1 ful* mutants show indeed woody growth reminiscent of the perennial lifestyle. Hence the FLAs upregulated at the top of the stems might belong to a regulatory circuit controlling elongation and suppressing secondary growth.

The genes in group V show the presence of a conserved motif putatively recognized by MYB3, which is an R2R3 MYB transcriptional repressor belonging to subgroup S4 together with the characterized AtMYB4 [44]. MYB4 negatively regulates phenylpropanoid biosynthesis (more specifically, in thale cress it is a negative regulator of hydroxycinnamic acid metabolism and it exerts its silencing function by displacing the activators binding to the MYB motifs present in many promoters of genes involved in the phenylpropanoid metabolism; [44]). It is therefore possible that the identified element is involved in the coordination of phenylpropanoid biosynthesis in bast fibres and might regulate the hypolignification observed in these cells [45, 46]. In our recently-published transcriptomic dataset [47], we observed an upregulation of the SOC1 gene at the top (4-fold induction with respect to the bottom and 1.3-fold induction with respect to the middle) and MYB4 at the bottom (1.7-fold induction with respect to the top and 4.6-fold induction with respect to the middle). This result therefore strengthens the existence of a putative regulatory circuit (controlling, among other genes, the expression of *CsaFLA*s) at the top and bottom of adult hemp plants.

## Conclusions

In conclusion, our work has identified (at least) 23 genes coding for FLAs in textile hemp, some of which specific to distinct stages of bast fibre development. Bioinformatics has highlighted the occurrence of conserved motifs in the promoters of genes upregulated either at the top or at the bottom of the stem. This finding points to the existence of a fine regulatory network controlling bast fibre elongation and cell wall composition. Future functional analyses carried out on heterologous systems will shed more light on the functions of the identified genes.

## Additional files

Additional file 1: Table S1. List of primers used to amplify *CsaFLA*s in the study. The details concerning the primer sequences, amplicon length and Tm, PCR efficiency and regression coefficient are given. (DOCX 16 kb)

Additional file 2: Table S2. Primers used to amplify three representative *CsaFLA*s promoters. (DOCX 11 kb)

Additional file 3: Table S3. The coding sequences of the 23 *CsaFLA* genes. (DOCX 21 kb)

Additional file 4: Figure S1. Details highlighting the intron-exon structures, CDS and 5’-3’UTR of *CsaFLA*s. The sequence order follows that of the branches in the maximum-likelihood phylogenetic tree performed with the full-length nucleotide sequences. (DOCX 142 kb)

Additional file 5: Table S4. CsaFLAs from *Cannabis sativa*. FAS domains are in turquoise, AGP domains are in red, signal peptide are in green and GPI anchors are in purple (the color-code is as after [7]) (DOCX 28 kb)

Additional file 6: Figure S2. Schematic representation of CsaFLA domains. The details relative to the signal peptide, GPI anchor, AGP and FAS domain(s) are indicated for the 23 CsaFLAs. (DOCX 86 kb)

Additional file 7: Figure S3. AGP immunodetection in hemp stem with LM14 antibody. Inset shows a detail of bast fibres. Scale bars are 200 μm in the main picture and 50 μm in the inset. (DOCX 1645 kb)

Additional file 8: Figure S4. Expression analysis of 22 *CsaFLAs* in fibres (A) and stem core (B) of *C. sativa*. Error bars indicate the standard error of the mean (*n* = 4). Different letters indicate statistically significant values at the one-way ANOVA test (*p* < 0.05). (DOCX 169 kb)

Additional file 9: Figure S5. Expression analysis of 23 *CsaFLAs* in hemp leaves (A), male and female flowers (B), in roots (C) seedlings (D). Different letters indicate statically significant values at the one-way ANOVA test (*p* < 0.05). (DOCX 239 kb)

Additional file 10: Promoter sequences of a *CsaFLA* subset. (DOCX 18 kb)

Additional file 11: Alignment of the promoters amplified from Santhica 27 with those from Finola and Purple Kush. (DOCX 18 kb)
