# Supplementary material for: Identification of fasciclin-like arabinogalactan proteins in textile hemp (Cannabis sativa L.): in silico analyses and gene expression patterns in different tissues
Source: BMC Genomics. 2017 Sep 20;18:741. doi: 10.1186/s12864-017-3970-5 (PMC5606014; doi:10.1186/s12864-017-3970-5)
Supplement: Supplementary file 1 — List of primers used to amplify CsaFLAs in the study. The details concerning the primer sequences, amplicon length and Tm, PCR efficiency and regression coefficient are given. (DOCX 16 kb) [file 12864_2017_3970_MOESM1_ESM.docx]

| **Name** | **Sequence**  **(5′→3′)** | **Amplicon length**  **(bp)** | **Amplicon Tm**  **(°C)** | **PCR efficiency (%)** | **Regression coefficient (R^2^)** |
| --- | --- | --- | --- | --- | --- |
| **FLA1Fwd** | CGATTTCACAGTCCAGAACG | 94 | 83 | 94 | 0.998 |
| **FLA1Rev** | TGGTTGTTCGTCGATGAGTG |  |  |  |  |
| **FLA2Fwd** | TGCAAACTGACGGTGAAGAC | 84 | 81.2 | 97.7 | 0.997 |
| **FLA2Rev** | ACGGCTCCTTGTCAATCAAC |  |  |  |  |
| **FLA3Fwd** | GCCCAAGCACCAATTACATC | 136 | 86 | 96.6 | 0.997 |
| **FLA3Rev** | TTTTGAGGAGGTGGACGAAG |  |  |  |  |
| **FLA4Fwd** | GGCGATGGAAGCAGACTTAC | 76 | 78.7 | 100.1 | 0.982 |
| **FLA4Rev** | GAGAGGAGAAAGAGGAGGAAGAG |  |  |  |  |
| **FLA5Fwd** | TTATCACCGTCGCAATCTCC | 90 | 84.4 | 106.9 | 0.943 |
| **FLA5Rev** | TGTTGGGGTTGACTCAGATG |  |  |  |  |
| **FLA6Fwd** | CAACAATGCTCTGAGGATGC | 90 | 80.9 | 95.5 | 0.995 |
| **FLA6Rev** | GGTGGCTTAGCTCCAAAAAG |  |  |  |  |
| **FLA7Fwd** | TCCATTCTCCACCTTCCTTG | 133 | 81.3 | 98 | 0.976 |
| **FLA7Rev** | CTGCTTAAGGGATGCGAAAG |  |  |  |  |
| **FLA8Fwd** | ACGATCACAGCCGTTAAACC | 75 | 81.7 | 100 | 0.989 |
| **FLA8Rev** | TGGAGCTGGTTTCTTCAACC |  |  |  |  |
| **FLA9Fwd** | GCTGCCATTTTTAAGTTGTGAC | 89 | 76.3 | 98.4 | 0.997 |
| **FLA9Rev** | CCGATCCAACTCACTTCATC |  |  |  |  |
| **FLA10Fwd** | GCTTTAGCTGCCATTATTCGAG | 71 | 77 | 101.8 | 0.989 |
| **FLA10Rev** | CATTTTGGGGTGTGGAACTG |  |  |  |  |
| **FLA11Fwd** | TTTTGGAACGGCTACAGCTC | 129 | 85 | 93.3 | 0.996 |
| **FLA11Rev** | GCACCAGAATCGTCATCATC |  |  |  |  |
| **FLA12Fwd** | GCGAAAAATCAAATTTTGTTCAC | 72 | 76.8 | 92.3 | 0.996 |
| **FLA12Rev** | ATTTTCGCAATCATGGAAGAG |  |  |  |  |
| **FLA13Fwd** | TACAACGGAGGGAAACTTCG | 142 | 78.6 | 94.5 | 0.998 |
| **FLA13Rev** | CGCTTTCGAGCCAAAGATAC |  |  |  |  |
| **FLA14Fwd** | GCTGATCAACGAGGGTGAGA | 142 | 82.1 | 86.3 | 0.999 |
| **FLA14Rev** | AATGCCCGATACTTGGACCG |  |  |  |  |
| **FLA15Fwd** | CATTTTCGCTCCCACTAAGC | 104 | 83.6 | 100 | 0.983 |
| **FLA15Rev** | ACGAATCTGACCCTTTACCG |  |  |  |  |
| **FLA16Fwd** | TCATGCCCCATCCCAATCAC | 87 | 81.5 | 93.2 | 0.998 |
| **FLA16Rev** | GCCCCCGAAGAAGACTTTGA |  |  |  |  |
| **FLA17Fwd** | CAGTTAGGCGATTTGGGAAG | 82 | 80.8 | 94.4 | 0.993 |
| **FLA17Rev** | ACCGAACCATCAGCTTCTTG |  |  |  |  |
| **FLA18Fwd** | GTCCGAAACCGAAGACAAAG | 122 | 80.6 | 92.5 | 0.992 |
| **FLA18Rev** | ATCCACATCCGAGCTAATGG |  |  |  |  |
| **FLA19Fwd** | ACATCATCATCGTCGCCAAC | 127 | 81.8 | 97.6 | 0.995 |
| **FLA19Rev** | GCAACAAGAATGGAGATCGTG |  |  |  |  |
| **FLA20Fwd** | GACATGTTGTGCCGAGATTG | 85 | 81.3 | 103.7 | 0.987 |
| **FLA20Rev** | CCCCTTGAAAAAGTCGGAAG |  |  |  |  |
| **FLA21Fwd** | GCACTCCAAAACCGAACATC | 100 | 81.6 | 98.5 | 0.998 |
| **FLA21Rev** | GACGTTGAAGCCAGCTTTTC |  |  |  |  |
| **FLA23Fwd** | ACTATCAATGGCGGCTTCAC | 85 | 78.6 | 106 | 0.994 |
| **FLA23Rev** | AATGGTGTTTGAGGGGAGTG |  |  |  |  |
| **FLA24Fwd** | CGATCTCCTTCGTATGGTTG | 83 | 79.9 | 92 | 0.965 |
| **FLA24Rev** | TGACACCTCTGGAAGCCTAAG |  |  |  |  |
