# Supplementary material for: Identification of fasciclin-like arabinogalactan proteins in textile hemp (Cannabis sativa L.): in silico analyses and gene expression patterns in different tissues
Source: BMC Genomics. 2017 Sep 20;18:741. doi: 10.1186/s12864-017-3970-5 (PMC5606014; doi:10.1186/s12864-017-3970-5)
Supplement: Supplementary file 2 — Primers used to amplify three representative CsaFLAs promoters. (DOCX 11 kb) [file 12864_2017_3970_MOESM2_ESM.docx]

|  | **Name** | | **Primers (**5'→3') | |
| --- | --- | --- | --- | --- |
|  | **CsaFLA2-F1** |  | GTGTCTTTCGAGCTAGTCTA |  |
|  | **CsaFLA2-R1** |  | ACGGCTCCTTGTCAATCAAC |  |
|  | **CsaFLA2-F2** |  | TCTTGTGTTGTGTCGTGCTT |  |
|  | **CsaFLA2-R2** |  | CATTTTGACGAACAGTACTG |  |
|  | **CsaFLA7-F1** |  | TATGTTTAGTAGTGGAGTGA |  |
|  | **CsaFLA7-R1** |  | CTGCTTAAGGGATGCGAAAG |  |
|  | **CsaFLA7-F2** |  | ATGATTATTTTATTTTATTA |  |
|  | **CsaFLA7-R2** |  | ACATACTTGTACGCCATTTC |  |
|  | **CsaFLA16-F1** |  | ATTTGCTTGCAATGGGTGGCA |  |
|  | **CsaFLA16-R1** |  | GCCCCCGAAGAAGACTTTGA |  |
|  | **CsaFLA16-F2** |  | GTGTAAAGATACTTTTGAGG |  |
|  | **CsaFLA16-R2** |  | GTGTTTGGTATTCATGATGA |  |
