# Supplementary material for: Identification of fasciclin-like arabinogalactan proteins in textile hemp (Cannabis sativa L.): in silico analyses and gene expression patterns in different tissues
Source: BMC Genomics. 2017 Sep 20;18:741. doi: 10.1186/s12864-017-3970-5 (PMC5606014; doi:10.1186/s12864-017-3970-5)
Supplement: Supplementary file 3 — The coding sequences of the 23 CsaFLA genes. (DOCX 21 kb) [file 12864_2017_3970_MOESM3_ESM.docx]

| **Name** | **Coding Sequence** |
| --- | --- |
| *CsaFLA1* | ATGCAGCTCCGTCCGGCCTTAACCGCCGGGACACTGGTCCTAGCTATGGCCGTCATCTTCATCTTCTCCGGCACCGGTGTCGATGCTCACAACATCACGAAGATTCTCGCTAAGCATCCGGAGTTTTCTTCATTCAACCATTACCTAACCCTAACTCATCTCGCCTCAGAAATCAACCAGCGGACGACGATTACTGTCTGCGCCGTTGATAACGCCGCCATGGGCGATATTCTCTCGAAGCATCCGAATATCTATACTGTAAAGAATATTCTCTCGATTCATGTTCTTCTCGATTATTTCGGTGCCAAGAAGCTTCACCAGATCACTAACGGTACTGCTCTTGCTGCTACTATGTTTCAAGCTACCGGTTCCGCTCCTGGATCTACTGGATTCGTTAATATCACAGATCTACACGGTGGAAAGGTTGGATTCGCTCCTGAAGATAACAACGGAGCTTTTGCTGCGCATTTTGTGAAATCTGTTGAGGAAATTCCTTACAATATATCGGTGATTCAGATCAGCGGTGTTTTGCCTTCGGCTGCGGCTGCGGCGCCGACTCCAGCTCCGGCTGAGATTAATATTACCGGCATTATGTCGGCTCACGGCTGTAAAGCCTTCTCTGATGCTTTACTTGCTAACGACGCTATGGAAATTTACGAAGATGCCCTCGCTGGTGGTTTAACTGTGTTTTGTCCTTTGGATGATGCGTTCAAAGCTTTTTTGCCTAAATTCAAGAATCTAACCAAATCAGGGAAGAATTCGTTGTTAGAGTACCACGGAGTACCTGTTTACCAATCCATGTCGATGTTGAAATCAAACAACGGACTCATGAACACTCTCGCTACAGACGGAGCTAGCAAGTTCGATTTCACAGTCCAGAACGACGGAGAGCAAGTGACTCTCAAAACGAAGCTCGTCACGGCCAAAATCACCGGCACACTCATCGACGAACAACCAGTAGTGATCTACACTATCGATAAGGTATTAATGCCTAAGGAGCTTTTCAAGCACGAGAAAGCAGAGACTCCAGCTCCAGCTCCGGCACCGGAAAAGGCAGCAGACGCACCTAAATCTTCTAAGAAGAAAGGCAAGAAAGCGGCGCCGTCACCTGACGACGACGCCGATGCCGACGCTCCAGCACCAGACGATGATGACGTCGCAGATCAGACTGCTGATGATAACGGCGCCGTTAGATTTGACGGTAACAGATTTAGCTTCTTCGCTCTTATCGCTACTGCATGGCTAGGATTTTCAGTTCTGTAA |
| *CsaFLA2* | ATGCCTCATCATCACCAAAAATTTCTCCTCCTCTTCTTCTTCTTCCTCGTGGCAACCACATCCCACGCCCACAACATCACGAAAATCCTAGCCAAGCACCCCGAATTGTCGACCTTCAACCAGTACCTGAGCCGAACTCGGCTGGCGGCCGACATCAACCGCCGGCTGACCATCACAGTCCTGGCGGTGGACAATGCAGGAATGTCATCTCTCATCTCAAAGGGCTATTCTCTCTACACTATTCGCAACATCCTCTCACTCCACGTCCTTGTTGACTATTTTGGTGCGAAAAAGCTCCACCAGATCAGCAAGGGCTCCACCTTGACTTCCAGTGTCTTCCAAGCTAGTGGTGCCGCCCCAGGCACCTCCGGCTTCGTCAACATCACTGACCTCAAGGGTGGTAAGGTCGTATTTGGTGTTGAGGACAATGATGGTCATCTCACTTCTCACTTTGTCAAGTCCATCAAGGAGATTCCATACAATATCTCCGTCATTGAAATCAGTCAGGTGTTGAGTTCGGCCGAAGCCGAAGCGCCGACTTCAGGACCAAGCGAGTTGAACGTAACGACTATATTGTCAAAGCAAGGCTGCAAATCTTTCGCCGACTTGTTAATAGCCACTGGAGCCGACGCCACTTACCAGTCCAACACTGAGTCTGGCTTAACGGTGTTTTGTCCAACAGACGGTGTTGTCAAAGGTTTCATGCCAAAGTACAAAAACTTAACGACTGCCAAAAAAGTATCGTTACTGTTATATCACGGCATCCCCGTTTACCAATCCATCCAGATGCTTAAGCAAAATAACGGAGTCGTTAACACGCTGGCTACCGACCGTGCCAATAAATATGATTTCACGGTGCAAACTGACGGTGAAGACTTGACGTTAGAGACTACGGTGGTAACGTCGAAGGTTACTGGGACGTTGATTGACAAGGAGCCGTTAGCCATTTATAAGCTGAATAAGGTTTTGTTGCCTAAGGAGCTGTATAAGCCGACTGAAGCCACTTCGCCCAAATCCAGTAGCGATGACTCCGACGACGAAGAAGCCGATGCCCCAGAGGGTGATTCTGATGATCAGACGGCTGACGATAATGGTGCCGTTGGAATAAACGGTGGGAGAATGGCTGTGGTCTTTTTGAGCTTGTGCGTCGGATTTTTGCTTATGTGA |
| *CsaFLA3* | ATGAAAAAACAAGGCCTCATCTCCCTTTCCTTCATTTCCATCTTTTTCTCCATAGCCCTAGCTCAATCTCCAACCCAAGCTCCAACCCAAACCCTAGCCCAAGCACCAATTACATCACCAACACCATCCGAAGCGCCACTAGTCCAACCGCCGGCCCTGGCAAACCCTACAAACGCCACCGAGATCCTCGAAAAAGTCGGGGGATTCAGTGTCTTCGTCCACCTCCTCAAAACAACATCAGAGAATATTCAAATCGAAAACCAACTCAAATACATCTCAAACAGCTTGACAATCTTAGCCCCATCAAACAAAGCATTTTCCAATCTCAAACCAAACACTCTAAACTCACTCACCACAAAAGAAAAACTCCAACTCATACAAAACCACATCATTCCTTCCTTCATACCAATCCAAAATTTCCAAACCCTAATAAACCCTGTACGGACACAAGCAAATTATTCTCTTAATATTTTGGTCGAAGGTAGTTGGGTTAACATATCCACAGGAGTTGTTAATGCAACAATTAATGCAACAATTTATGAGGATAATCAGTTAGCTATTTACAAGGTCGATAAGGTACTTCTTCCTCTTAGAATTTTCCGTGTAAAACCGCGAAAAAAGGCGGTGGGTGCACCGGCACCGGCTCCCATATCAAGTATGGTTGTTAAGCCTGATGAGTTTCCAACATCTTCGTTGATTGCTCCTGCTTTGGCTGCTTTGCTTAAAGATGCTTCTGCTGTAAGTGGTGCCCTTTGTCTTAGTGGAAATGGGATTTTGAGCTTTGGAATTGCTGTGGTTTACGTGGTACTACTTTCTCTTTTTTAG |
| *CsaFLA4* | ATGGCGATGGAAGCAGACTTACCCATTTCCCATTTTACCCCTACACCATCCATACTCTTCCTCCTCTTTCTCCTCTCTTCCGCCATAGCCACCGCTCCCACCGCCGCTCTCAACATCACCAACCTCCTCTCCTCCTTCCCAGACTTCTCCGACTTCGCTTCACTCCTCTCCTCCTCATCATCTTCCCTTGCCGCCGATCTCTCCCGCCGATCCTCCATAACTCTCCTCGCCGTCCCAAACACATACCTCTCCGCTTCATCAGATCTCACGCGCCGCTTATCTTCATCCTCACGCGCTGATGTCGTCCGATACCACATACTCCTCGAATACCTCTCCCCTTCCGATCTCCGCCGGATCTCTTCCTCAGGTAAGCTTGTCACCACGCTCCTTCAAACCACCGGACGCGCCACCAACAACTTCGGTTCCGTCAACATCACCCGCGATCCAATCACCGGAATTGTTTCAGTCCGATCTCCGGCACCTTATTCACCCTCGAATGCTACCATTCTTAACCCTATCAAAACCCTACCGTACAATGTGAGTGTCTTCGCTGTTAATTCTCTTCTCGTTCCGTACGGATTCGATCTAATGGCTTCCGAGACTCGACCGCCATTAGGTTTGAATATCACTAAGGCTTTAATCGACGGTCATAACTTCAACGTTGCGGCTTCGATGTTAGCAGCTTCCGGCGTAGTAGAGGAATTTGAAGCCGACGAAGGTGGTGCCGGAATAACCCTATTTGTACCAGTGGATACTGCTTTCGCTCAACTTCCGTCAAATGTTGAGCTTCAATCATTACCGGCCGATAAGAAAGCAGTGGTGTTGAAATTCCATGTTCTTCATTCTTATTATCCTTTGGGTTCGCTTGAATCAATTGTGAACCCAGTTCAACCCACATTAGCTACGGAAGACATGGGCGCAGGAAGCTTCACTCTCAACATTTCTAGGGTTAATGGATCGGTGGCAATCAATACAGGTATCGTTCAAGTGTCGGTGACCCAAACAGTTTTTGATCAAAACCCAGTCGCCATTTTTGGGGTTTCTGGGGTTTTATTACCCAAAGAGATTTTTGGGAAGAATCCAATAACGACGACGACTCCCAAACCAGGAACACCCTTTGCTTCGGGTACGGCTCAGCCACCTGATATTGCTTTCTCGCCGGAGTTTACGCCTGGATTAGATGGCCCTCCTTCACACCTCTCATCTCCGCCGGGTTTAGGTCAAGATATAAGATCGAAAGCAGAAGCAGCAGCCGCCATTAATGGGTTCCATTTCTATACCTTGATCCTTGCTCTGTTGTGTATAGCCTTATCTCTTTAAATGGTAAATGGTATGAGATATAATTTATTATTTTGGTTGGTAATTAGATTTTATTTTTTTTAATTTATTTATTTTTGTGTTTTTTATTTTTTTTTTTACACGGGAAATTGAATTTATAA |
| *CsaFLA5* | ATGGCAATGGCCCTCTGTCTCTACCTCCCAGCTCTCCTACTCTTCTTCCCAACCCTTCTCTCAGCTTCTTCTTCTTCTCCTTCCACTACATTATCACCGTCGCAATCTCCACCATCTCCTTCTCCACCGCCACCGCCGCCTTCTCCTTCACTCCCATCCCCATCTGAGTCAACCCCAACATCTCCTCAATCACCACAACATTCATCCCACCGGAGGAGACACCGAAGATCCCGGCGAAGGCAACAACAACCTCCTTCGACAGCGGAGACGCCTCAGCAATTCAACAACATAATCGATGCCTTGATTGGCTCCGGAGACTTCAGCAATTGGGTCAATGTCATTTCAAACGCCGTTCTCCCTCTTAGCGCAACTCTCTTCGTCCCAGAAAATGAAGCTGTAACTGCCCCGACGATCGCCAGTCCCGGTCCGGAAGACCCATTGATTTTCCCTTACCATGTGGTACCACAGAGGCTCTCTTTTGCCGAACTACTTCTCTTCCAGACCAACACTCGCCTTCCCACGCTCCTCCCTGGGAAGTCTATCATCATCACCAACAACTCCCGGATCAACTTCACCATCGACGGCTCTCTGATCACTCAGCCGGATATTTACTCCACCGGCAACATTGCCGTTCACGGCGTTGGCTCTGTCTTTAATTACTCGGTCTTCGGTGATGGTTTAGACCTTCTGCCCAAATCCTCGAACCCAGAGCCGAATCAGTCGCAAGTTCGCCGGCCGCCAACGGTGGATCACCCGAACGGAGAGACGTATGGTTCGAGCTCGGACTCCGCGCCGCCGTGCCTGTGCATTGAATTGCCGGTTGTTTTCTTAGTGTTCTGTGGGGTTTTGATGTTCAAGATTCAGAGAAATGGCGGCCATGGACGGTGA |
| *CsaFLA6* | ATGGCTTTCACACCTCTCTCTATCCTCCTCCTGACTCTCATCACCATCTTTTCCCACCAAATTTCAGCCCAAGCCCCGGGCCCCGCCCCGGCCGGCCCCCTAAACTTCACCGCAATCCTCGAAAAAGGTGGCCAATACACCACCTTCCTCCGCCTTCTCTCCGACAGCCAAGTATTAAGCCAAATCGTAAACCAACTCAACACCTCCTCCGAAGGACTAACCGTCTTAGCCCCAACCGACAATGCCTTCAACAACCTCAAGGCCGGAACCCTAAACGGCCTCTCCCGTGAAGACCAAGTTAACTTAATCCTCTTCCACGTTCTCCCCAAGTACTACGCCCTATCAGAACTCCTCACCGTATCCAACCCCGTCAGAACTCAGTTCTCTGCTGATGGCCTTAACTTCACCGGTCAAGGCCGTCAAGTCAACGTCACCAGCGGAATGGTGGAGACTCAGGTCAACAATGCTCTGAGGATGCAATCCCCATTGGCTGTTTACCAGATCGACGATGTTTTGTTGCCACCAAGTCTTTTTGGAGCTAAGCCACCGGCCTCTGCTCCTCCTCCGGCCAAGACTCCGGCATCCAAGGACGACGGAGATAAGACCAAGCCTAAGGCATCCGGACCATCGTCCGATGATAGTACTGGGGATTCGAGTAATACACGTGTAGGGTTGGGATTGTTTGTTGGGATGGGTATTGCTTGCATGGCTGTTCTTTTTTGA |
| *CsaFLA7* | ATGGCGTACAAGTATGTTTTCATTGCCAGCAGTATGCTGCTGCTTCTGTGCTCATCAGCAATGGCTCAAAAAGCTGCTTCACCACCTCCCCTAATCCTAACTCCAACTCCGGCACCAGCCCCAGCTCCTGATTACGTGAATCTGACCGAATTACTTACTGTGGCCGGTCCATTCTCCACCTTCCTTGACTACCTTGTATCCACCAAAGTGATTGAAACCTTGCAAAACCAAGCCAACAACACTGAAGAAGGTGTAACCATCTTTGTCCCAAAAGACAGTGCTTTCGCATCCCTTAAGCAGAAGCCCTCTTTGAAAAACATCACCAATGATCAGCTCAAGTCACTGTTGCTGTTCCACGCTTTGCCACATTACTACAGCTTAGCTGAGTTCACCAATCTTAGTCAAAGCAGCCCAATCAACACCATGGCTGGTGGTCCATTCACTTTGAACTTCACTGATGTTTCAGGGACTGTCCACATCGGCTCGGGTTGGACCAACACAAAGGTCAGCAGCAGTGTTCATTCAACTGACCCTGTTGCCCTTTACCAGGTTGATAAAGTTCTCCTCCCTGAGGCCATTTTTGGAACTGATATCCCTCCAACCCCAGCTCCAGCACCATCCCCAGATATTGCTCCTGTTGCAGATGCGCCAGGAGATGAGGGAACTGATGCCAAGTCCCCATCATCTTCTACTTCACAAGGGAAGTCTGCTTCTCACAGGGTCAACAGCTTGGGAGTTTTGTGCCAAATGGTTTTGGCTGTTTCAGGTGGGCTGGCCTTGTTCTTGTGA |
| *CsaFLA8* | ATGGGTACTCATATCCGTGGCGACCAAAAGCTTGTCTTTTTCTCATTCTTCTTCATCACATTCGCCGGAATATGCTTCTCATTGCCGGAAAATGTCAACCCCAGGTCATTGTTCTCTTCCTCCAACGCTTCCACATCATCGGGTCAGATCAACTCCAACTCGGTCCTGGTCGCTCTCCTAGACTCGCATTACACTGAGCTCGCTGAGCTAGTCGAGAAGGCTCTGCTTCTCCAGACACTTGAGGAAGTCGTCGGCGCCGCCAACGTTACCATTTTCGCTCCACGCAACGAAGCCCTCGAGCGAGGACTTGACCCGGAGTTCAAGCTCTTCTTGCTCGAACCTGGTAATCTCAAATCGCTTCAAAAGCTTATCTTGTCTCACATCGTACCGACCCGGATCGGATCCAACGATTTACCCAAGAAACCCGACTCTGCTCACCACAGAACTCTTTCCCACGAACACATCCACCTAGAAAAGCAAGATTCCGGTGAGTGGACTGTGGATCTCGCTCGTCTCACTCATCCCGACTCGGTGACCCGACCCGATGGTGTGATCCACGGGATCGAAAGGGTTTTGATTCCTCGCTCAGTAGAAGACGATTTCAACCGTCGACGTAGTCTACGAACGATCACAGCCGTTAAACCAGAAGGGGCTCCAGTGGTTGACCCGAGAACAAACCGGTTGAAGAAACCAGCTCCACCTACCAAACCCGGTTCAGAACCGGCTCTACCGATCTACAACGCAATGGCTCCGGGTCCATCTCTAGCTCCAGCCCCAGCACCCGGACCCGGTGGCCCTCACCACCATTTCGATGGGGAGAGTCAAGTCAAGGACTTTATCCAAACCCTCCTTCATTACGGCGGTTACAATGAAATGGCTGACATTTTGGTTAACTTAACTTCGTTAGCCACCGAAATGGGTCGATTGGTTTCAGAGGGTTACGTACTGACTGTGCTGGCACCAAACGATGAAGCCATGGCTAAGCTTACTACTGACCAGCTCAGTGAGCCAGGAGCACCGGAGCAGATAATGTACTATCACCTCATACCGGAGTACCAAACTGAAGAGAGTATGTACAACTCAGTTCGCCGGTTCGGGAAGGTGAAATACGACACACTTCGATTACCACACAAGGTTTTGGCCCAAGAAGCTGATGGGTCGGTTCGGTTCGGGCATGGGGAGGGTTCGGCTTATTTGTTCGACCCAGATATCTACACCGACGGTCGGATTTCGGTTCAGGGAATCGATGGTGTTCTGTTCCCGCCTGAGGAGGTTGAATCCAAACCCGTTTCCCAAACGGTTCAACCCGCCAAAATTGTAGCCAAGCCCAAAAGAGGAAAATTGCTAGAAGTAACATGTCAAATGCTTAGAGTCTTTGGAAAGGATTCGCAATACCCGACATGCCACTAA |
| *CsaFLA9* | ATGCAAAATAACCAAAAACACAAACCAATAAATCCTAACAACAAAAACTCACTACACCAAAACATGAACACATCAAAAATGCTCATCAACACTCCCTCACTTGTTCTCATTCTCCTCCTCATTTTCATCACAAAATCTTCAGCTCAAACCGCGGCCCCGGCTCTCCCACCCGGTCCCCCGCCCTCGACAGACATCTACAAAATCCTAACAAAAGCCGGGCAATTCACCGTCCTAATTCGCCTACTCAAGAGCACACAAGTTGGTAACCAAATCAATAACCAACTCGGAAGCACAAACAGTGAACTAACCATGTTTGCCCCGTCCGATAGCGCCTTTTCGAACCTCAAAACCGGAACCCTAAACGGCCTAACCGACCAACAAAAAGTCCAACTCCTTCAATTCCACTTAGTCCCATCATTCATTTCAATAACCAATTTCCAAACAATGAGCAACCCTGTTCAAACTCAAGCCAGTGACACTTATGAGTACCCTCTTAACATAACAACCTCAGGAAGCCAAGTCAACATAACAACTGGAATAGTCAACACAACAATTTCAGGAACAGTTTATTCTGATAACCAATTGGCTGTTTACCAAGTTGATAAGGTTCTTCAACCACTTGGGATTTTTGCTCCAAGGCCTCTCCCACCAGCTCCTGCCCCAGCTCCACCTAAGGCTAGTAAGAAAAAAGCTACTGATGCTGATACCACTGGGCCTGCCACGTCGGATGACAGCTCGGATGCCATTGGTGGTAGTAATGGAGGTTTGGGCAAGGTGGTATTGTCTTTGTCTATGTCTATGTTTGTTGCTGCCATTTTTAAGTTGTGA |
| *CsaFLA10* | ATGCAAAGCTTTAGCTGCCATTATTCGAGTATACAAAACACCGTTCGATCAACAATTTCAGTTCCACACCCCAAAATGGTCGCACTAAACCATTATCTCATCTTCTGTCTTCTATTCGCCCTAAACGCCGTCGTTTCAGCCCACAACATCACAGAAATCCTCTCCGGATTCCCAGAGTACAGTGATTACAATAACTTTTTATCCCAAACAAAGCTCAGCGATGAAATCAATAGCCGCCAAACACTAACTGTCCTTGTCCTGAGTAACGGAGCTCTTTCCTCCCTTACAGCCAAACACCCTCTCTCCGTTATCAAGAAAGCCCTTAGCCTTCACGTCGTTCTGGACTACTTCGACCCGAAAAAGCTCCACCAGATCTCTCAGGGAAGCACCTTATCCACTACTCTTTACCAGACTTCCGGAAATGCCCCTGGAAATCTTGGCTTCGTCAACATCACCGACCTTAAGGGCGGCAAAGTTGGCTTCGGTTCTGCAGCTCCCGGCTCCAAGCTCGACTCCACCTACGTTAAGCCAGTCAAGCAGATTCCGTACAACATTTCGATCCTTGAGATCAGCGCGCCAATCATTGCGCCAGGGATCTTGACGGCTCCGGCTCCATCGGCTTCAGATGCTAATATAACGGCATTGCTTGAGAAAGCTGGGTGTAAGACATTTGCTTCGTTGATCGTTTCAAGCGGTGTGATTAAGACTTATATGACGGCGGTCGAGAAAGGTTTGACTCTCTTTGCACCGAACGATGAGGCTTTCAAGGCTGCTGGAGTTCCGGATCTGAGCAAACTCACCAACGCTGAGCTAGTTTCGGTCTTACTATATCATTCCCTGGCTGGTTACTCTCCCAAGGGAACGTTGAAGACCACGAGCAGCCCGATCAAAACCCTGGCTACCAACGGGGCCGGAAAATACGAACTGACTGTATCAACTGCCGGTGACGCCGTTACTCTCCACACAGGAGTCGACTCTTCCAGACTTGCCGACACGGTAACTGACTCAACCCCACTTGTCATATTCACTGTCGACAATGTCCTACTTCCAGCAGAGTTGTTCGGTAAATCGCCATCGCCTGCACCAGCTCCAGAACCAGTGAGTTCTCCTTCTCCGTCACCGGCACTTGCTCCGACTCCGGGACCAGCGACCGAAGCCCCAACCCCTCTCGGCGCTTCCCCACCGGCACCGCCAATGGAAACTCCTGGAGGAGCACCGGCGAACTCTCCCGAAGCTGATGCTGAAAACAGTACCGCAAAAGGAGCTGGTCACATGCACGCGCCTGCATTGCTGACTGCACTTTTCACTATCTCTGCCACTGTCACCTTCTCAATCTTCTTGTCCTAA |
| *CsaFLA11* | ATGGAGAAAATCACAAGATTAGCTCTACTAATAACCACAGCCATTTTCCTCCTTTGCAAACCTATCTCAGGCCAAAGTCCGGCGAAGTCACCGGCGCCAGGAGGCCCTGTTGACATAATAGCTGTTCTTAAAAAAGCAGGGCAATACACCACATTCATCAAGCTTCTAAAGGGTACCCAAGTATCTGACCAAATCAATTCCCAACTTAGTGGTTCAAGTCAGGGCATTACAGTCTTTGCACCCACCGACTCAGCCTTCTCTAGCCTCAAGACAGGCACTCTCAACTCCTTGACCAGTGAACAACAACTTCACCTAGTTCAGTACCATGTCCTCCCAGCCTTTTATACCATCTCCCAATTCCAAACTGTCTCCAACCCAATTCACACCCAAGCTGGTAATAGCGAAAATGGCCAGTACCCACTTAATGTAACAACCTCGACTAGTAACCAGGTCAACATTACCACTGGTGTGGTCAATGCTACCGTCAGTAATACCGTCTATACTGATGGCCAGCTTTCAGTATTCGAAGTCGACCAAGTTTTGCTTCCTTTAGATATTTTTGGAACGGCTACAGCTCCGGCACCGGCGCCCGTGGCTGATTCTAAGCCTGTTAAGAGTGTTGACCAAGATTCTGATGATGCTCCGGCCAAGTCTAAGGGCCCCGATGATGATGACGATTCTGGTGCTTTGAGCCTCAAATCAGGCTCAATTGGTGGGCTTTTATTTGCTGGAGCAGCTCTTCTTGCAGTCTTTTAA |
| *CsaFLA12* | ATGGCGAAAAATCAAATTTTGTTCACCTATTCTTGCGCAATAGTTTTCCTCTTCCTCTTCCATGATTGCGAAAATACTTTAACATTAGCTCAATCGTCATCACCTGCCCAATCACCATCGACAACACCTAATAAGAATCATCATCCTTCATCTGATTCCTCAGCTCAATCTCCGGATCAGCCATTAGTGGAAGCCCCACCAGCAGCGAAATCCAGGAGAAAAGGACAACCCACCAACATAACAGAAATCCTAGAGAAAGCCGGCGGTTTCAGCATCTTCATTCGCCTACTAACAAGCACTGACGTCATCAGTCCCGTCGAAAATGATCTCAACTCTTCCAATACCGTCACCATTTTGGCTCCAACCAATGCAGGATTCTCAGCTCTTAAGACCGGAACACTCAACACTCTCACCCCCCAACAAAAAACACAATTGGTTCAGTTCCACATCATCCCAACTTTCATATCCCTACAGAATTTCCAAACCCTAACAAACCCTGTTCACACTCAAGCCACTAACACTCGCGACTACCCATTAAACATTACTAGTACTGGCGGATCTTCTGTCAACATATCTACTGGAGTTGTCAACACAACCATATCTGGAACCATTTATTCCGATAACCAACTCGCTATTTATCGAGTTGATAGTGTCTTGCTTCCTATGAAACTTTTCGCTCCTAAGAAGGTGGTGAGCTCACTGGCACCGTCTCCAGCACCCGCAATGGCCTTAAAACCTAAAAAGAAATCGACGGAATCAGCTCCATCGTCTGGATCGCCCTCATCCTCCTCAAAACCATCTTTAACATCTACATCACCCACATCATCATCATCATCCAATGATGATGAATCAGTCGCCACATCAGCAGATACATCTGGTGCTGAACGACCAGTTGTTGTTGTTGCCACAGTCTCCGTTGGTCTTCTTGCTTTGTTCTTATGA |
| *CsaFLA13* | ATGATGAAACAAGTTATCATCTTGTCCTTCTTCATTGTTTTCCTCTTTCACTCTTCTTCTACTTTAGCTCAATCTCCAGCTCACTCCCCAACTCAGCCTCAAAAACCAATTCCTAAAGCTCAATCGCCTACTACTAAACCCGTCCTTGCTCAACCGCCATCTCAAGCAGTACTACCGGCTCCTTCTCAAGCTCCAACCCAAAAGCCTCTCCCTCACACCCCACCGCGAAAGCCAACCCCTAAGCCAGCCCCGCCTAATGTCACTGAGATCCTTGACAAAGCTGGAGGCTTCAGTGTCTTCGTCCGCCTCCTTAAAAACACTCAAGTCGTCAATCAGATCGAAAATCAACTCAACACTTCCAACAGCTTAACCATTTTGGCCCCAACCAACGGCGGATTCTCTAGTCTAAAAGCCGGCGCTCTCAACGGCCTTACCCCTGAGCAAAAAGTCCAACTCCTTCAATACCATATCCTTCCATCCTACGTTCCCCTCCAAAACTTCGAAACCCTAACCAACCCTGTCCGTACACAAGCCAGTAACACTGAGGATTACCCTATGAACATTACAACGGAGGGAAACTTCGTAAACATTTCTACTGGAATTGTTAACGCTACACTTTCTGGTACAGTCTACTCTGATAATCAACTAGCCATTTATAGAGTTGATACTGTTCTTCTTCCTTTAGGTATCTTTGGCTCGAAAGCGCCTACTCCTTCTCCTACTCCTGCTCCTACTCCAGCTCCACTCGCGCATTTAAAACCTAAGAAGTCTTCTACTCCAACAAGTACTTCATCATCATCCTCAGCTGCTTTGCCTTCCAACTCATCATTGGCACCTAAAGCGCCTAAAAAGCCATTAAATTTGTCGTCGACCACCTCTTCCTCGATCACTCCTGTGGCGGCATTGGATGAATCCGGTGTTGTTGCCCTAACTTCAAGTAATGGCGTCGTGATTGGCCTTGTTGTGACTGGGGTCGCTGCAGTTATGAGTATTTTGGGTTAA |
| *CsaFLA14* | ATGATTAAGAAGATGACTAATCCCAAAGCCTCCTCCTCTTCTATTCTCTTCATGCTCGTTGCCATTTTCTTATTCTCTTCATTATCATCATCCTTAGCTTTCAACATCACAAAACTCCTTGGCCAAAATCCTGAGCTTAGTACATTCAACAACTACTTAACTCAGACAAAGCTTAACGATGAGATCAATCGTCGCCAAACTATCACTGTCCTCGTTGTCGATAATTCCGCCGCAGCTTCCCTCTCCGGTAAGTCACTCGATGTAATTAAGAAAATCTTAAGCGTTCATGTGATTTTGGATTACTACGATGTTGAGAAGATTACCAAGCTTACTACCTCGAAAAAGACATCTACCGTCACTACTCTCTTCCAAGCCTCCGGCTCCGCCGTTGATCAAGAGGGTTTTCTCAAAGTCGCGCTGATCAACGAGGGTGAGATCGCCTTCGGCTCTGCCGTAAAGGGTGCCTCTCTAAACGCCAAGTTAGTCAAATCTGTGGCCGCGCAGCCATTCAACATCTCCGTTCTACAGATCACTGCGCCGGTCCAAGTATCGGGCATTGAGTCCAGCCCATCACCACCATCTCCAAAGGCAGCTACGCCTTCTGCTGCTCCCAAAAGGGCTCCCGCCCCGTCAAACAAGTCCGGGGCTGCTCCTTCCCCATCCAAAAATTCTGGGGCCAATACTCCCGCCACCGCACCTAGTACTGCTGATGCACCGGTTGCCGATACGCCGACAACAGCCGCAACTTCGCCTGCGCCTGCTGCTGCTGCTGCTGACGAACCCGTTGCTGATAATGCCCCGGTGAGTTCGGCTCCTCCGCAAGCTGATGCTGCAGCTGATCCACCGGTTTTGTCAAATTCCGGGGTGGTGAGGCAGGGGATGAAGATAGGTATTGTGGCGGCGGCATTGATGTCGTGGTTGGTTGTTTAA |
| *CsaFLA15* | ATGAAGCAAACTCTCATCTCTTTTTCATTCCTACTTCTCATCTTATCTCACACCACAATGAGTTTAACTCATCAAGCTCCAGCTAAAGCCCCATCTGCCCACATAGCCGCTACTAAAGGCGCCACCCCAGCCGTGGCGCCTACAAAACCTAAAGTAGCAACACCAACTCCAACAACATCCCCATCTCGAGCCCCGACCTCATCAGAAGCACAAGCTCCCTCAGCAGAGCCACTAGTTGAAGCTCCACCGCGAAAAGCCAAACCCGAGCCAACCGATGTGGTTAAGATTCTTGACAAAGCCGGAACCTTTAGTGTCTTCATCCGCATACTCAAAAGCACCCAAGTAATAGAACAGATCGAAAACCAACTCAACACTTCCAACAGCATGACAATTCTAGCCCCAACTAACGGCGCTTTCTCCGCTTTAAAACCCGGAACACTCAACTTCTTAAATGCAGAACAAAAGGTACAATTAGTACAATACCACATCTTACCTTCTTTCATTTCCATCTCGAGTTTCCAGACTCTCAGTAACCCTGTCCGTACACAAGCTAGTAACACTGACGAGTACCCTTTAAACATCACAACACAAGGTAATTGGGTTAACATATCAACTGGTGTTGTCAATACAACTATTTCTGGCACAATCTATGCCGATAGCCAACTCGCTATTTATAAAATCGACAAGGTTTTGCTTCCCATGGCCATTTTCGCTCCCACTAAGCTTCATAAGGCCCTTGCACCTGCGCCTGCATTGCTTGCTAAGCCTACAAAAGGCTTAGCAGGTAGCGGTAAAGGGTCAGATTCGTCATCTGACCCCTCATCGTCTACTTCAACATTGGATGATAACCCAGTTTCTTCAATGCATGCCTCTAGTGCTTTTCGTAATGGCATGGTCGCAACAATTGGTGTTATTAATGTTTTTGGTGCAATTGCAATTTTATTTTGA |
| *CsaFLA16* | ATGAATACCAAACACTTTGTCACGATCTTCTCATTCCTAACCCTAATCTTCTTCCATGCAACAACTTTAGCCACAACCCCAACTGCTCATGCCCCATCCCAATCACCAGCACAAGCACCAGCTAAACCCTTATTAGCTCAACCTCCAAAGAAGTCAAAGTCTTCTTCGGGGGCGCCTGCCCTAGACTCAGCGGCCTCTTCTGCTCCACCCTTGTCACAAGAGCCAATAGTCCAAGCTCCACCCCACAAAGGCCGAAGCAGGATTCCCACCGACGTGGCAGGAATCCTCGAAAAAGTAGGAGGCTTTAGTGTCTTCAACCGCCTCCTTAAAAGCACCGAAGTACTCACTCAAATCGAAAACCAACTGAGTGCCTCAAACAGCTTAACCATCTTAGCCCCAACAAACGATGCCTTCGCCTCTTCACTCAAACCCGGAACACTCAACACCTTAACCAAAGAACAAAAAATCCAAATGATACAATACCATGTTTTACCAACTTTCATTTCTCTTTCGAATTTCCAAACCCTAAGCAACCCTGTCCGTACACAAGCTGCTAATACGTATGATTATCCTATGAACATTACTACCGAAGGTAATTGGGTTAACATATCGACCGGTATTGTCAATGCTTCTATTACCGCCACTGTGTTCTCTGACGATCAGTTGGCTATTTATAGAGTGGATAAGGTTTTGCTTCCTCTCGGTGTTTTTGCACCGAGACCAAAGCTTCAACCTTCTCCTTCACCCTCTGCTCTTTTGGCTAAGCCAACAAAAGATTCTTCATCGAATTCATCATCATCATCGAGTTCTTCTTCAATGTCCTCCAGGGCGGATGGCCCTGGTGGAGCAAGTGAGGATAATGATGATGATCAGAAGACGAATAATGCTTCCAGTGCATCTGATTTTACTATTGGAGCTCGAACGATGTCGTTTGGAGCTGTAATAGTTTCCATGGTCGCAATAAAATACATTCTTGTGCTTTTTTAG |
| *CsaFLA17* | ATGGATACTCATGGCTATGGCGTCTCCTTCTTATTATTCTTTATTCTTTGTTCCTTTACTAACTCTTTCGCTGCATTGCCCAACAACCCATCTCAGAAAAGTAATTCTACTTCTTCCGGTTCTGGTCAGATTAATTCTAACTCGGTTCTGGTTGCGCTTCTCGACTCGCATTATACTGAGTTGGCCGAGTTAGTCGAGAAAGCCCTTTTGCTACAGACCTTAGAAGAGGCCGTCGGTAAACACAACATCACCATTTTCGCCCCGAGAAACGAAGCTCTAGAACGCCAACTCGACCCTGAATTCAAGCGATTCCTGCTCGAACCCGGGAACGTAAGGTCTCTCCAGACACTCTTAATGTTCCACATTATCCCCAAACGTATCGGCTCCGGCGAGTGGCCCGCTTCCGATTCCGTTCCGGTTAGACACAAAACTCTTTGGAACGACCGCGTTCATTTAACGAGCAAAAACTCCGGCGAAAAGGTTATCGACTCGGCCGAGATAGTCCGTCCCGACGACGTAGTAAGACCCGACGGTGTAATCCACGGAATCGAAAGTCTTCTAATCCCACGTTCAGTTCAAGAAGATTTCAACAGAAGAAGAAGTCTCCGATCAATCTCCGCGGTATTGCCGGAAGGTGCGCCGGTAGTTGATCCCAGAACCCACAGGTTAAAAAAGCCAGTTGCCCCTGTTCCGGCTGGCGCACCCCCAGTTCTTCCAATCTACGACGCTTTGGCTCCAGGTCCATCTCTAGCTCCAGCTCCGGCTCCGGGACCAGGTGGGCCGCGTGGCCACTTCGACGGAATGGCTCAAGTCAAGGACTTTATCCAAACCCTCGTGCATTACGGTGGGTACAACGAAATGGCTGATATTTTAGTTAATCTAACGTCTCTGGCCACCGAGATGGGTCGGCTCGTATCGGAAGGTTATGTAATTACGGTTTTAGCCCCGAACGACGAGGCCATGGCTAAACTGACTACGGACCAGCTTAGCGAGCCTGGGGCACCGGAACAGATTGTGTACTACCACATCATACCAGAGTACCAAACCGAAGAGAGTATGTACAATGCAGTTAGGCGATTTGGGAAGGTTCGTTACGATACCTTGAGGCTACCCCATAAGGTCATGGCTCAAGAAGCTGATGGTTCGGTGAAATTCGGCCATGGCGATAGCCAGGCTTACCTTTTCGACCCAGATATCTATACCGATGGCCGGATTTCAGTTCAGGGCATTGATGGGGTTCTTTTCCCACCTAATGAGGACCCCAATTCGGAGAAGAAAACAACTCCTCTTGTTAAGGTTGTCACCAAGCCCACCAGAAGAGGAAAGTTGATGGAAGTGGCCTGCAATGTGCTTGGTGTTTTTGGTGTATCATCTTCATGTCAATAA |
| *CsaFLA18* | ATGAACAAGCAAGCAATCATTTACTTCTCATTCTTGTTTTTGTGTTTCTTTTACCACTGCAAAGGAGCCACAATATTAGCTCACTCTCCAGCCCAACCTCCATCAAAACATGTAGCTGCTGCAGCCCCAACTAAAGCTAAGGCCTTAACCCCAACGAAAGCGCCAACAGCTTTGCCAGTGCCCGCGGTGGAGCCGCCATCTCAAGTGCCACTCGTCCAAGCGCCACCCCACAAAGCCCTTTACACACCAACAGACGTCACCAAAATCCTCGAAAAAGCCGGTATCTTCAGTGTCTTCATCCGCCTCCTTAAGAGTACTTCTGTAAGCATTCAGATTGAGAATCAACTGAATGTATCCAACACATTGACCATTTTTGCCCCAACAAATGGGGCCTTTGGTGCTCTGAAACCTGGCACTCTCAACACACTCTCTAATGAAGACAAAGTCCAGCTTGTCCAATACCATATTCTTCCCTCTTTGGTTTCACTACAAAACTTTGAGACTCTTAGCAATCCTGTACGGACACAAGCTAGTAATACCAATGATTTTCCTCTAAATGTTACTGTGGAAGGAAGCTCTGTTAACATATCTACTGGAATTGTTAATGCTACCATTTCTGGTACTGTTTATGAGGATAATCAACTTGCTATTTACAAGGTTGATAAGGTGTTGTTGCCTTTGGGAATCTTCGGTCCGAAACCGAAGACAAAGCAGCACCTAGCGCCTTCTCCCACACCATTGAAGCCATCTAAAGATACTAATGTGTCATTACCTTCTTCCTCTACTGAAGAATCCATTAGCTCGGATGTGGATGAAGGGGACAAGTCCTCAAAGTCGAAGGCTGCTGTACTTATGAATAA TGGTGTTGTA AACATTGGAG TTGTTATGAT TGTTGTAATCACTATGTGGG GTCATTTTTAG |
| *CsaFLA19* | ATGGCAAAAAATCAGATTTTGATCACCATTTCGTGCCTAATTTTATTCCTCTCCCTCTTCCATACTCTGACATTAGCTCAGTTGTCATCACCTGCCCAAGCACCATCAACAACACCAAATAAGAAGAATATTCATCCGTCATCACATCAATCGCCAGCTCAATCGCCGGATCAGCCATTGGTGGAAGCTCCACCAACGGCTATTTCCAGGAGAAAAGGACATCCCACCAACATTACAGAAATCTTGGAGAAAGCCGGCGATTTCAGCCTGTTTATTCGTCTACTAACAAACACTGATGTCATCACTCCCATCGAAAATGATCTCAACTCTTCCAACACCGTCACCATTTTTGCTCCAACCAATGGAGCATTCTCAGCCCTTAAGACTGGAACACTCAACACTCTCACCCCCCAACAAAAAACACAACTAGTCCAGTTTCACATCATCTCAGCCTTCATACCCCTGCAAAACTTCCAAACCCTAACAAACCCTGTTCATACTCGAGCCATTAACACTCGCGACTTCCCATTAAACATTACTAGTACTGACGGATCTTCTGTCAACATATCCACTGGTGTTGTCAACACAACCATATCTGGAACTGTTTATTCCGATAACCAACTCGCTATCTATCGAGTTAACAATGTCTTGCTTCCTATGAAAATTTTTGCTCCTAAGAAGTTGATGAGCTCACTGGCACCGTCTCCAGCACCCGCGGTGGCCTTAAAGCCTAAGAAAAAATCGATGGCATCTGCTGAATCGTCTGAATCACCCTCGTCCTCATTAAAACCATCTTCAACATCTACATCATCATCGTCGCCAACATCATCATCATCATCATCCAATGATGATGAAACAGTAGCCACTTCGACAAATACATCTGGTGCTGAACGGGGGCCAGTATTTGCCACGATCTCCATTCTTGTTGCTGTTGTTGCATTGTTCCTATGA |
| *CsaFLA20* | ATGGCCAAATCTCTCTTCTTCTTCTTCATTCTTTCTATCTTCTCCTCCTCTGCTCTCGGCTCTTGCTTGACCCTTTTAAACGCCGCCGAGATTCTCTCAAACTCCGGCTACCTCTCAATGTCCTTGACCCTTAAAATCGCCTCTCAAACCATTAAACACGATTCACCGACCGCCACCGTCTTTGCTCCGGCAGACCAAGCTTTCGTCAAGTCAGGCCAACCTTCCCTGTTTCTCCTTCGCCGCCACGTGTCCCCCGTAAAACTCTCACTCGAAACACTCAAGACTCTTCCTCGTGGCTCAGTAATACCCACCATGGTCCCCGACCACCCTCTAATCGTCACCGCTTCTCTCTCCGGCGACGGCTACATCTCCATAAACGACGTCAGGATCAACGAGAAAGCCGTTTTTGGTGNTGGGTTCGTAGCTCTGTACGGTATAGACGAGTTCATTGACTCATCTTTCTTCCGGACTGATCAGCCGCCGTCCCCAGCTCCGGCTCCGGCGCCTGCCCCGTTCCACGGCAAGACAGAGTCTTTTGCTTCGGTTGCTGAGTTTCTCAGGTCGAGAGGTTACTATATAATGGCCACGCTTCTTGATGCCCAATTGACCGGGCTCGGTGACGGGACCAAATTGACCATCTTTGCTCCGGTGGACCGAACTTTCGATTACTATGCATCGAATATAAGCGATTACGCTTTGATTTTCCGGAGACATGTTGTGCCGAGATTGATGACGTGGCAGGACTTGATTGGAAGCCAAGTTGTTGGGACAAAGCTTCCGACTTTTTCAAGGGGTTTTATGATCGAGGTGAGGGTGTCAAGTGATGGTATTCCTATGCTTAACGATGCACCAATTGTGTTTCAAGATATATATCGAAGTCAAAGGTTGATTGTTCATGGCCTTAATGGGTTTCTCAAGCCTTTCACTGATCAAGAATGGAATCAAGATTCTTTCTCTAATGGATTTGTTGGTGATGATTCTCATGGATAA |
| *CsaFLA21* | ATGGCTCATTGTTGCACCGGCTCGTGGCGTGCACCGGTCTACTTCACCGTGTCTGTAATCTTGGCCTTCATAGCCATCTCAACATCAATGCACTCCAAAACCGAACATCCATCCTCCCCAACCAAACTCAATTTCCATGAACTCTCCCTCAACGCCTCCAAAGCCTTAAGAAAAGCTGGCTTCAACGTCATGGCCACCCTTCTTCAAGTCTCCCCTGAGATCTTCCTCTTATCTCCAAACTCAACTATCTTTGCCATTCAAGATGATGCCATCTCCAACTCTTCACTCCCACCATGGCTTTTGAGAAACCTCCTTCAATATCACACCTCTCCTCTCAACCTTCCCATGAAAGACCTCTTAAAGAAGCCTCGAGGGAGTTGCTTGCCGACCCTTCACCGCCAAAAGAACATTGCCATCACTAACATCGACTTCAAAGAAACAACAGTTGATATCAACAACGTTTCAGTGACTCATCCTGATGTATTTCTTGCAGAAACCATTTCCGTCCACGGTGTTCTTGAGCCCTTTTCTTCGTTGGATCCTGAAGATGTTCATCAGGGTTGGAACTCTATCCAAGCCCCCACTTGCAACGCAATGTCTGTTCTAGTTTCGGATGCTGTGAAATCCACCAACATGGTTGAATGGTCGTGGATTGTCAGGTTGCTGAGCTCGAACGGATTCGTTCCTTTCGCTATAGGATTAAATTCTGTTCTTGAAGAGATTCTTAAAGACTACAAGGGATTGAATTCTGTAACAATCTTTGCTCCTCCAAATTTGCAGTCTTTGACATCTCCTTCACCCCTACTCAAAAGAACAGTATGGTTTCACATACTTCCTCAGAGGTTAACGTATAAAGAACTTACTGCATTGCCTGCTGGAACCTTGCTCATGACATTGGTTCGTGATCTATCTCTCGAGGTTGCAGGGACAGCAGGTTTCAAAGGTGGACTAATCATAAATGGAATCGAGATCGTGGCGCCCGACATTTTCACTTCAAAGAAGTTCACCGTGCATGGGATTTCTCGAGCTTTTGAGGTTGCTGATCAGGTAGCTGCAATCGGTACATAG |
| *CsaFLA23* | ATGGCGGCTTCACTTCTCATCTCTCTAACTCTTCTCTCATTTCTTTCTCTCTCCTCCTCACTCCCCTCAAACACCATTATCGATGCCTCCGAGATTCTCTCCGACTCTGGCTTCGCTTCCATGGCTCTTACTCTGGATCTCGTCTCCCAAACCCTAACCCAAAGGACTCCTTCTCTCACGATATTTGCTCCGGCCGATGACGCCTTTAAGAGATCAGGGCAACCCGCTCTATCTCTCCTCCGTTACCACTTCTGCCCCCTCACTTTGCCGCTGGAGACCCTCAAATCGCTTCCCTCTGGGACCAAGATCCCGACTCTGTTACCCGGCCGCACCTTGACCGTCACTCATTCCTCCTCCACCTCTGAAATTTCACTGAACAATGTCAAAATCAGTAGAAGATTTCCGATCTTCGATGATGGTTCTCTGATTGTCTTCGGAGTTCCAGAGTTCTTCGATCCCAATTTTCAAGCTCCCGGACCCGGTAATAGCCCGAGGTTTGGTCCAAGATGCAAATCGTTGCCATCGAAGGCTGCCGCCATGGGGTTTCCGGGAGCTTCTTGGTTTAAAGAAGCCAGTAGAGATTTGAGGTCCAATGGGTACTCATCCATGGCTTCGTTTCTGGATTTGCAGTTGTTGGGGTTCAACAAGGACCCGACTACAATGACTGTGTTTGCTCCTAATGACATGGCCATGGCGAATCGTCCAACCGATCAGGCTCAAGACCCATCAATCTTTCTACGACATGTCGTTCCTTGCAAGCTTCTTTGGAGTGATTTGATCAATTTCACTGATGGAACGGTGTTGCCTACATACTCGGATGGGTTCACCATCACCATTACCAGATCGGGTAGTACATTGATGATTAATGGAATTCCTGTAACCGTTTCCAACCTCCATTATAGTGATTCTGTCGTTGTTCATGGCCTAAATGAGCTTCTTACTGGACAAGCAACTACATCTGGGTCAGGTTAA |
| *CsaFLA24* | ATGAAGATGAACAGAGGTCGGTTTTTGAAGACTTCGATCTCCTTCGTATGGTTGGTGGTGCTTTTTGGCTGCCTTTTTGTGGTTCTAATTTCTGTTCTTAGGCTTCCAGAGGTGTCAAATAGTAGAAAGGCAATTGGGTTGTATCATAATACAAAAACCAGAGAAAGTTCAGAGTCTAGCTCTATTGGGAAATTTGGAGAGAAGATGATTGAAATGTTGCCTGAAGATCTTGCTTTCACTGTTTTTGTTCCTTCAGAGAGAGCCTTTGAACGAGATTTAAGGCTAAGGAAAAACGAAAGTCTGGTTGAGAAGATGAATGATGATACTTATGCTGTAATTTCTAGAGTTCTGGGATTCTCGGCTGTTCCAAGGACAATTATCACAGATGATGTATCTTCTGGTGAGGAGATTTTGTATGACTCTATATCTGGGTTTGTATTGTATATTTCCAAAGATGTGGATGGAATGTTAGTGGTTAACAGAGTTCGTTCTGAAAAAGTAGATATCAAAAGGAACAAAATTGTTGTACATGTAATGGATGGGGTTATCATGGATGCTGAATTTGAAGAATCAGTTCAGCCTGATGATGAGGATGAAGAAAAGTGA |
