# Supplementary figures and images for: Identification of fasciclin-like arabinogalactan proteins in textile hemp (Cannabis sativa L.): in silico analyses and gene expression patterns in different tissues
Source: BMC Genomics. 2017 Sep 20;18:741. doi: 10.1186/s12864-017-3970-5 (PMC5606014; doi:10.1186/s12864-017-3970-5)

**
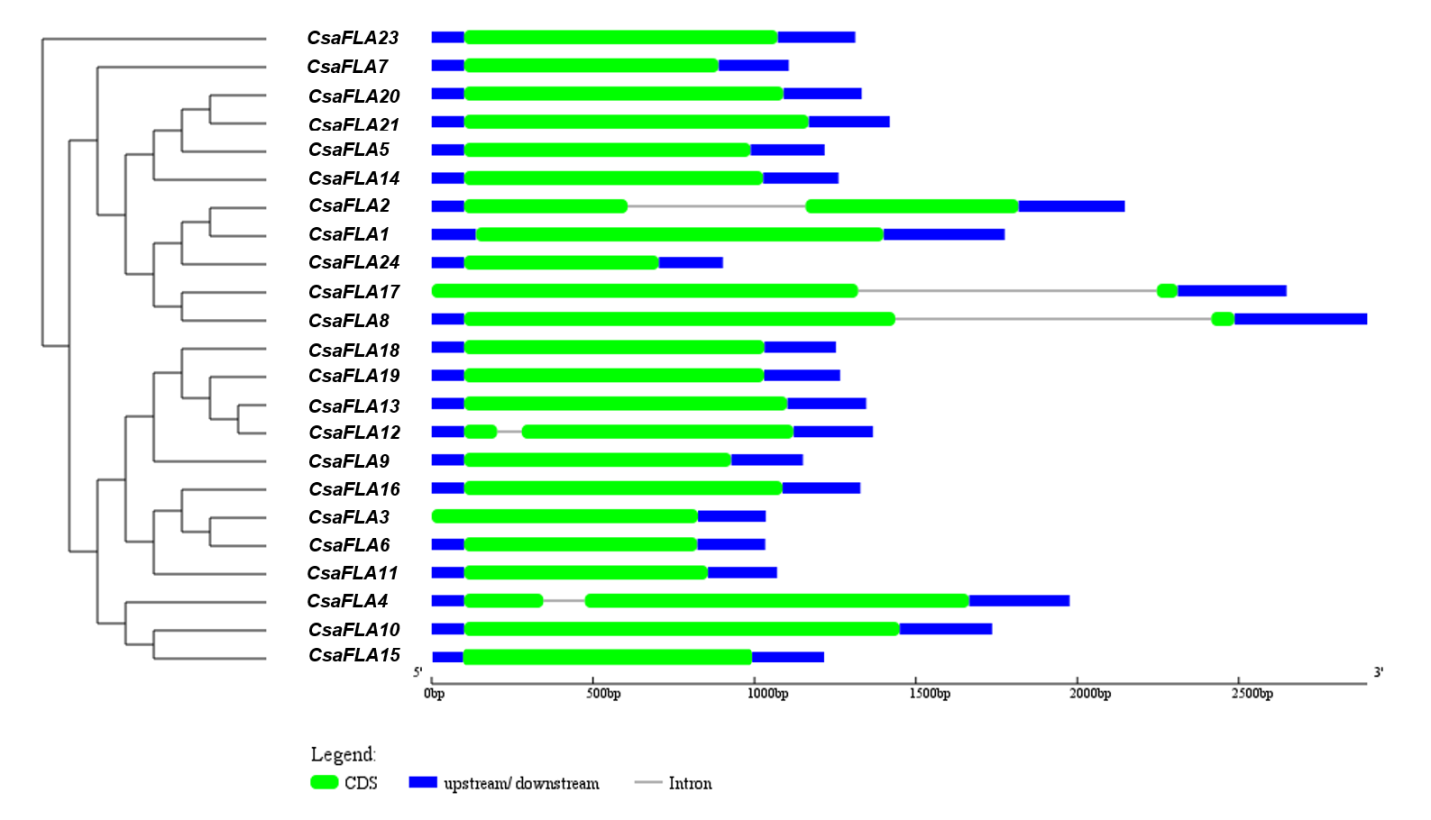
**

Supplement: Supplementary file 4 — Details highlighting the intron-exon structures, CDS and 5’-3’UTR of CsaFLAs. The sequence order follows that of the branches in the maximum-likelihood phylogenetic tree performed with the full-length nucleotide sequences. (DOCX 142 kb) [file 12864_2017_3970_MOESM4_ESM.docx]

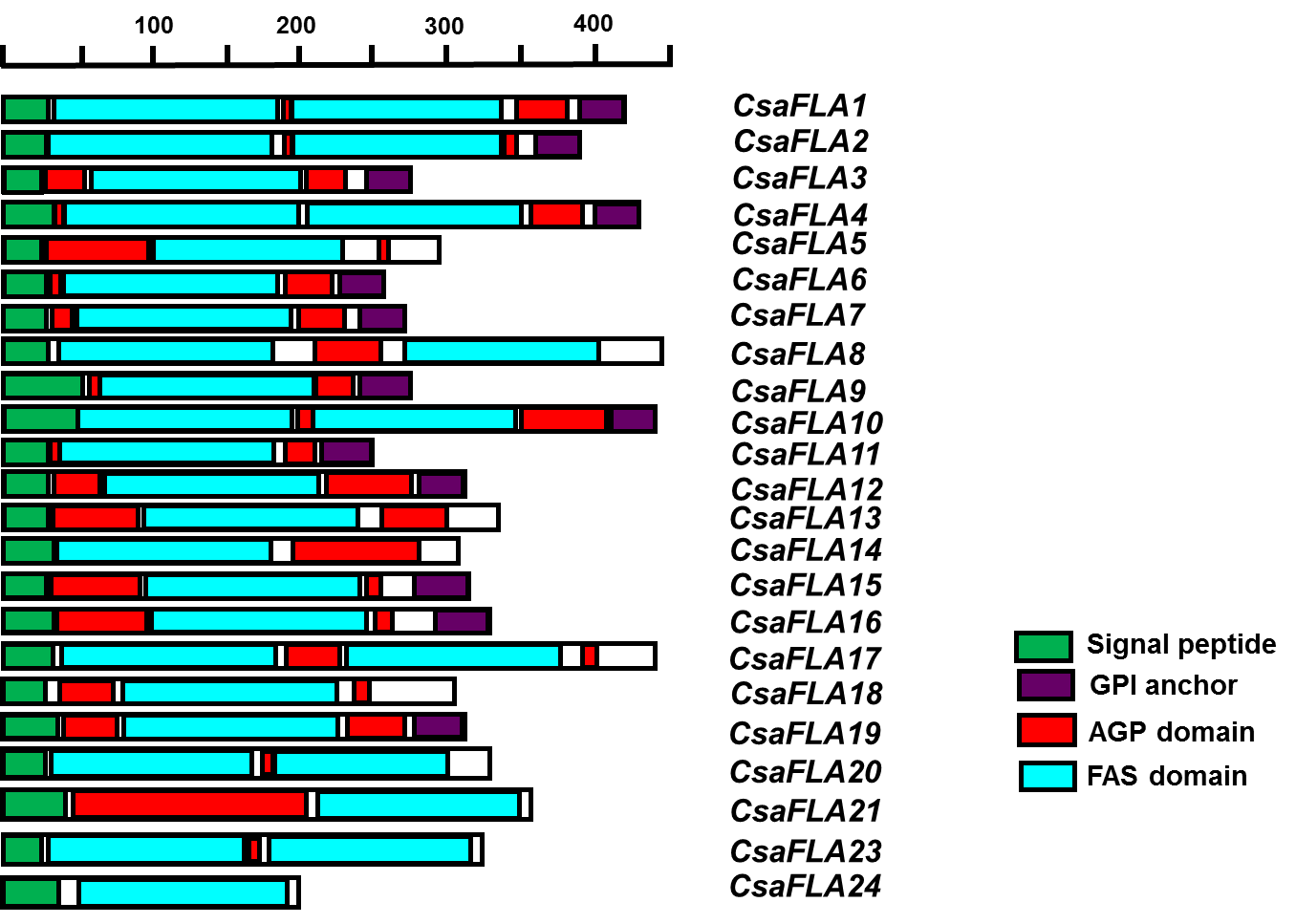

Supplement: Supplementary file 6 — Schematic representation of CsaFLA domains. The details relative to the signal peptide, GPI anchor, AGP and FAS domain(s) are indicated for the 23 CsaFLAs. (DOCX 86 kb) [file 12864_2017_3970_MOESM6_ESM.docx]

**
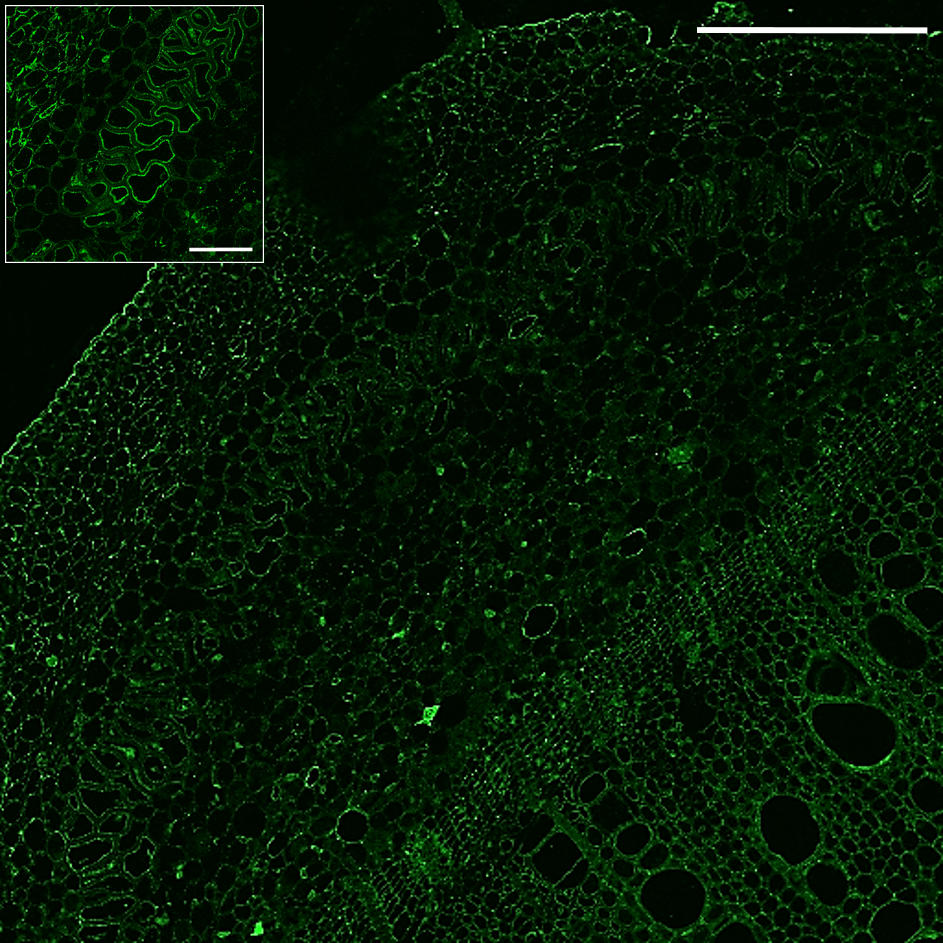
**

Supplement: Supplementary file 7 — AGP immunodetection in hemp stem with LM14 antibody. Inset shows a detail of bast fibres. Scale bars are 200 μm in the main picture and 50 μm in the inset. (DOCX 1645 kb) [file 12864_2017_3970_MOESM7_ESM.docx]

**
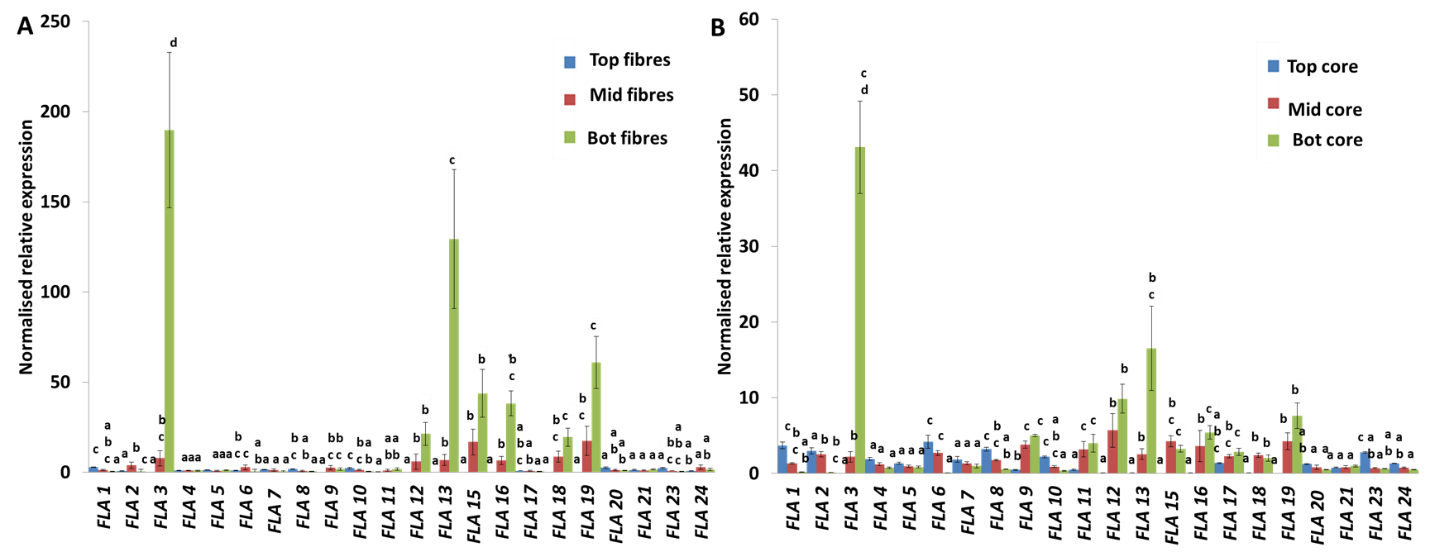
**

Supplement: Supplementary file 8 — Expression analysis of 22 CsaFLAs in fibres (A) and stem core (B) of C. sativa. Error bars indicate the standard error of the mean (n = 4). Different letters indicate statistically significant values at the one-way ANOVA test (p < 0.05). (DOCX 169 kb) [file 12864_2017_3970_MOESM8_ESM.docx]

**
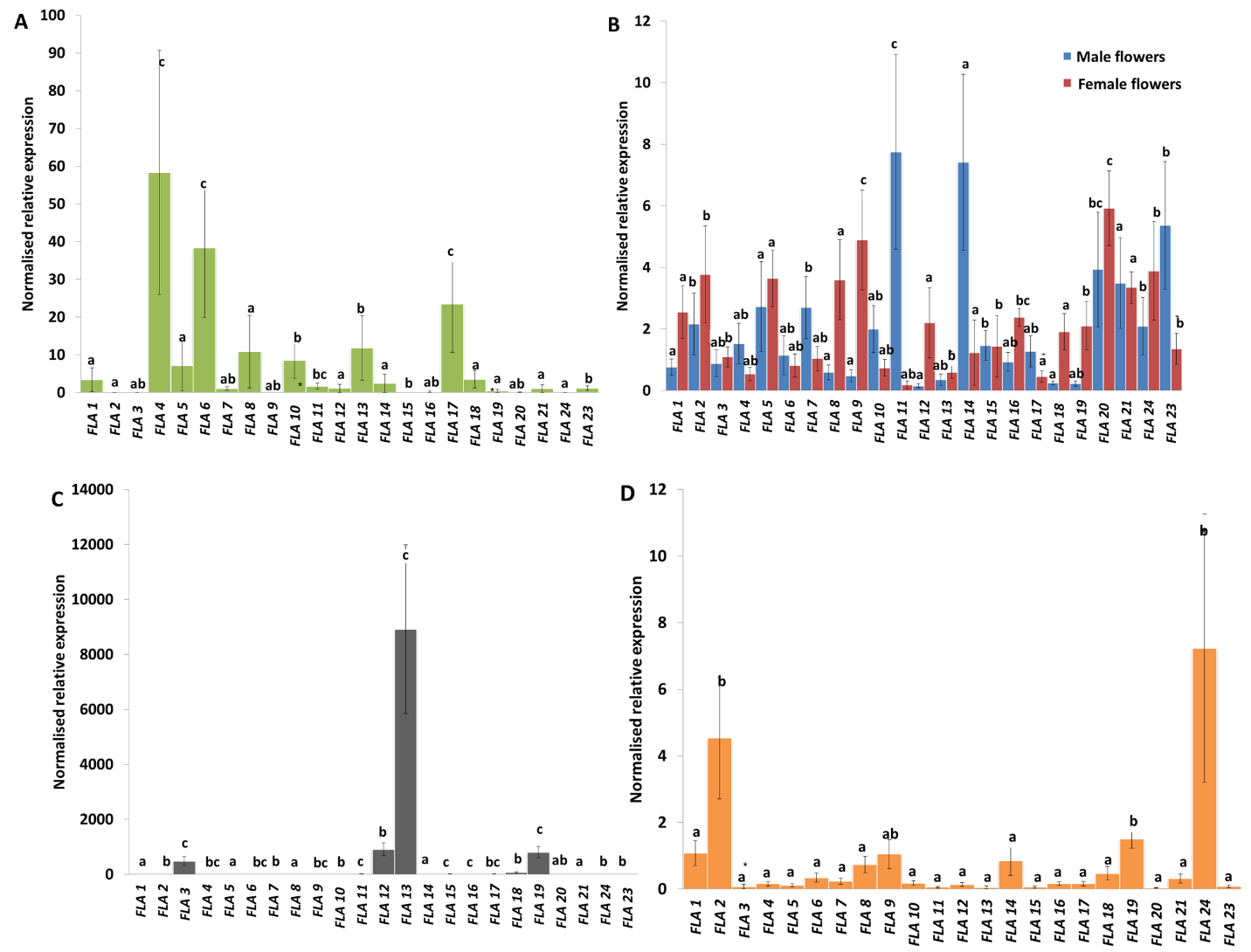
**

Supplement: Supplementary file 9 — Expression analysis of 23 CsaFLAs in hemp leaves (A), male and female flowers (B), in roots (C) seedlings (D). Different letters indicate statically significant values at the one-way ANOVA test (p < 0.05). (DOCX 239 kb) [file 12864_2017_3970_MOESM9_ESM.docx]
