# Supplementary material for: Identification of fasciclin-like arabinogalactan proteins in textile hemp (Cannabis sativa L.): in silico analyses and gene expression patterns in different tissues
Source: BMC Genomics. 2017 Sep 20;18:741. doi: 10.1186/s12864-017-3970-5 (PMC5606014; doi:10.1186/s12864-017-3970-5)
Supplement: Supplementary file 5 — CsaFLAs from Cannabis sativa. FAS domains are in turquoise, AGP domains are in red, signal peptide are in green and GPI anchors are in purple (the color-code is as after [7]) (DOCX 28 kb) [file 12864_2017_3970_MOESM5_ESM.docx]

| **Name** | **Precursor protein backbones** |
| --- | --- |
| CsaFLA1 | MQLRPALTAGTLVLAMAVIFIFSGTGVDAHNITKILAKHPEFSSFNHYLTLTHLASEINQRTTITVCAVDNAAMGDILSKHPNIYTVKNILSIHVLLDYFGAKKLHQITNGTALAATMFQATGSAPGSTGFVNITDLRGGKVGFAPEDNNGAFAAHFVKSVEEIPYNISVIQISGVLPSAAAAAPTPAPAEINITGIMSAHGCKAFSDALLANDAMEIYEDALAGGLTLFCPLDDAFKAFLPKFKNLTKSGKNSLLEYHGVPVYQSMSMLKSNNGLMNTLATDGASKFDFTVQNDGEQVTLKTKLVTAKITGTLIDEQPVVIYTIDKVLMPKELFKHEKAETPAPAPAPEKAADAPKSSKKKGKKAAPSPDDDADADAPAPDDDDVADQTADDNGAVRFDGNRFSFFALIATAWLGFSVL |
| CsaFLA2 | MPHHHQKFLLLFFFFLVATTSHAHNITKILAKHPELSTFNQYLSRTRLAADINRRLTITVLAVDNAGMSSLISKGYSLYTIRNILSLHVLVDYFGAKKLHQISKGSTLTSSVFQASGAAPGTSGFVNITDLKGGKVVFGVEDNDGHLTSHFVKSIKEIPYNISVIEISQVLSSAEAEAPTSGPSELNVTTILSKQGCKSFADLLIATGADATYQSNTESGLTVFCPTDGVVKGFMPKYKNLTTAKKVSLLLYHGIPVYQSIQMLKQNNGVVNTLATDRANKYDFTVQTDGEDLTLETTVVTSKVTGTLIDKEPLAIYKLNKVLLPKELYKPTEATSPKSSSDDSDDEEADAPEGDSDDQTADDNGAVGINGGRMAVVFLSLCVGFLLM |
| CsaFLA3 | MKKQGLISLSFISIFFSIALAQSPTQAPTQTLAQAPITSPTPSEAPLVQPPALANPTNATEILEKVGGFSVFVHLLKTTSENIQIENQLKYISNSLTILAPSNKAFSNLKPNTLNSLTTKEKLQLIQNHIIPSFIPIQNFQTLINPVRTQANYSLNILVEGSWVNISTGVVNATINATIYEDNQLAIYKVDKVLLPLRIFRVKPRKKAVGAPAPAPISSMVVKPDEFPTSSLIAPALAALLKDASAVSGALCLSGNGILSFGIAVVYVVLLSLF |
| CsaFLA4 | MAMEADLPISHFTPTPSILFLLFLLSSAIATAPTAALNITNLLSSFPDFSDFASLLSSSSSSLAADLSRRSSITLLAVPNTYLSASSDLTRRLSSSSRADVVRYHILLEYLSPSDLRRISSSGKLVTTLLQTTGRATNNFGSVNITRDPITGIVSVRSPAPYSPSNATILNPIKTLPYNVSVFAVNSLLVPYGFDLMASETRPPLGLNITKALIDGHNFNVAASMLAASGVVEEFEADEGGAGITLFVPVDTAFAQLPSNVELQSLPADKKAVVLKFHVLHSYYPLGSLESIVNPVQPTLATEDMGAGSFTLNISRVNGSVAINTGIVQVSVTQTVFDQNPVAIFGVSGVLLPKEIFGKNPITTTTPKPGTPFASGTAQPPDIAFSPEFTPGLDGPPSHLSSPPGLGQDIRSKAEAAAAINGFHFYTLILALLCIALSL |
| CsaFLA5 | MAMALCLYLPALLLFFPTLLSASSSSPSTTLSPSQSPPSPSPPPPPPSPSLPSPSESTPTSPQSPQHSSHRRRHRRSRRRQQQPPSTAETPQQFNNIIDALIGSGDFSNWVNVISNAVLPLSATLFVPENEAVTAPTIASPGPEDPLIFPYHVVPQRLSFAELLLFQTNTRLPTLLPGKSIIITNNSRINFTIDGSLITQPDIYSTGNIAVHGVGSVFNYSVFGDGLDLLPKSSNPEPNQSQVRRPPTVDHPNGETYGSSSDSAPPCLCIELPVVFLVFCGVLMFKIQRNGGHGR |
| CsaFLA6 | MAFTPLSILLLTLITIFSHQISAQAPGPAPAGPLNFTAILEKGGQYTTFLRLLSDSQVLSQIVNQLNTSSEGLTVLAPTDNAFNNLKAGTLNGLSREDQVNLILFHVLPKYYALSELLTVSNPVRTQFSADGLNFTGQGRQVNVTSGMVETQVNNALRMQSPLAVYQIDDVLLPPSLFGAKPPASAPPPAKTPASKDDGDKTKPKASGPSSDDSTGDSSNTRVGLGLFVGMGIACMAVLF |
| CsaFLA7 | MAYKYVFIASSMLLLLCSSAMAQKAASPPPLILTPTPAPAPAPDYVNLTELLTVAGPFSTFLDYLVSTKVIETLQNQANNTEEGVTIFVPKDSAFASLKQKPSLKNITNDQLKSLLLFHALPHYYSLAEFTNLSQSSPINTMAGGPFTLNFTDVSGTVHIGSGWTNTKVSSSVHSTDPVALYQVDKVLLPEAIFGTDIPPTPAPAPSPDIAPVADAPGDEGTDAKSPSSSTSQGKSASHRVNSLGVLCQMVLAVSGGLALF |
| CsaFLA8 | MGTHIRGDQKLVFFSFFFITFAGICFSLPENVNPRSLFSSSNASTSSGQINSNSVLVALLDSHYTELAELVEKALLLQTLEEVVGAANVTIFAPRNEALERGLDPEFKLFLLEPGNLKSLQKLILSHIVPTRIGSNDLPKKPDSAHHRTLSHEHIHLEKQDSGEWTVDLARLTHPDSVTRPDGVIHGIERVLIPRSVEDDFNRRRSLRTITAVKPEGAPVVDPRTNRLKKPAPPTKPGSEPALPIYNAMAPGPSLAPAPAPGPGGPHHHFDGESQVKDFIQTLLHYGGYNEMADILVNLTSLATEMGRLVSEGYVLTVLAPNDEAMAKLTTDQLSEPGAPEQIMYYHLIPEYQTEESMYNSVRRFGKVKYDTLRLPHKVLAQEADGSVRFGHGEGSAYLFDPDIYTDGRISVQGIDGVLFPPEEVESKPVSQTVQPAKIVAKPKRGKLLEVTCQMLRVFGKDSQYPTCH |
| CsaFLA9 | MQNNQKHKPINPNNKNSLHQNMNTSKMLINTPSLVLILLLIFITKSSAQTAAPALPPGPPPSTDIYKILTKAGQFTVLIRLLKSTQVGNQINNQLGSTNSELTMFAPSDSAFSNLKTGTLNGLTDQQKVQLLQFHLVPSFISITNFQTMSNPVQTQASDTYEYPLNITTSGSQVNITTGIVNTTISGTVYSDNQLAVYQVDKVLQPLGIFAPRPLPPAPAPAPPKASKKKATDADTTGPATSDDSSDAIGGSNGGLGKVVLSLSMSMFVAAIFKL |
| CsaFLA10 | MQSFSCHYSSIQNTVRSTISVPHPKMVALNHYLIFCLLFALNAVVSAHNITEILSGFPEYSDYNNFLSQTKLSDEINSRQTLTVLVLSNGALSSLTAKHPLSVIKKALSLHVVLDYFDPKKLHQISQGSTLSTTLYQTSGNAPGNLGFVNITDLKGGKVGFGSAAPGSKLDSTYVKPVKQIPYNISILEISAPIIAPGILTAPAPSASDANITALLEKAGCKTFASLIVSSGVIKTYMTAVEKGLTLFAPNDEAFKAAGVPDLSKLTNAELVSVLLYHSLAGYSPKGTLKTTSSPIKTLATNGAGKYELTVSTAGDAVTLHTGVDSSRLADTVTDSTPLVIFTVDNVLLPAELFGKSPSPAPAPEPVSSPSPSPALAPTPGPATEAPTPLGASPPAPPMETPGGAPANSPEADAENSTAKGAGHMHAPALLTALFTISATVTFSIFLS |
| CsaFLA11 | MEKITRLALLITTAIFLLCKPISGQSPAKSPAPGGPVDIIAVLKKAGQYTTFIKLLKGTQVSDQINSQLSGSSQGITVFAPTDSAFSSLKTGTLNSLTSEQQLHLVQYHVLPAFYTISQFQTVSNPIHTQAGNSENGQYPLNVTTSTSNQVNITTGVVNATVSNTVYTDGQLSVFEVDQVLLPLDIFGTATAPAPAPVADSKPVKSVDQDSDDAPAKSKGPDDDDDSGALSLKSGSIGGLLFAGAALLAVF |
| CsaFLA12 | MAKNQILFTYSCAIVFLFLFHDCENTLTLAQSSSPAQSPSTTPNKNHHPSSDSSAQSPDQPLVEAPPAAKSRRKGQPTNITEILEKAGGFSIFIRLLTSTDVISPVENDLNSSNTVTILAPTNAGFSALKTGTLNTLTPQQKTQLVQFHIIPTFISLQNFQTLTNPVHTQATNTRDYPLNITSTGGSSVNISTGVVNTTISGTIYSDNQLAIYRVDSVLLPMKLFAPKKVVSSLAPSPAPAMALKPKKKSTESAPSSGSPSSSSKPSLTSTSPTSSSSSNDDESVATSADTSGAERPVVVVATVSVGLLALFL |
| CsaFLA13 | MMKQVIILSFFIVFLFHSSSTLAQSPAHSPTQPQKPIPKAQSPTTKPVLAQPPSQAVLPAPSQAPTQKPLPHTPPRKPTPKPAPPNVTEILDKAGGFSVFVRLLKNTQVVNQIENQLNTSNSLTILAPTNGGFSSLKAGALNGLTPEQKVQLLQYHILPSYVPLQNFETLTNPVRTQASNTEDYPMNITTEGNFVNISTGIVNATLSGTVYSDNQLAIYRVDTVLLPLGIFGSKAPTPSPTPAPTPAPLAHLKPKKSSTPTSTSSSSSAALPSNSSLAPKAPKKPLNLSSTTSSSITPVAALDESGVVALTSSNGVVIGLVVTGVAAVMSILG |
| CsaFLA14 | MIKKMTNPKASSSSILFMLVAIFLFSSLSSSLAFNITKLLGQNPELSTFNNYLTQTKLNDEINRRQTITVLVVDNSAAASLSGKSLDVIKKILSVHVILDYYDVEKITKLTTSKKTSTVTTLFQASGSAVDQEGFLKVALINEGEIAFGSAVKGASLNAKLVKSVAAQPFNISVLQITAPVQVPGIESSPSPPSPKAATPSAAPKRAPAPSNKSGAAPSPSKNSGANTPATAPSTADAPVADTPTTAATSPAPAAAAADEPVADNAPVSSAPPQADAAADPPVLSNSGVVRQGMKIGIVAAALMSWLVV |
| CsaFLA15 | MKQTLISFSFLLLILSHTTMSLTHQAPAKAPSAHIAATKGATPAVAPTKPKVATPTPTTSPSRAPTSSEAQAPSAEPLVEAPPRKAKPEPTDVVKILDKAGTFSVFIRILKSTQVIEQIENQLNTSNSMTILAPTNGAFSALKPGTLNFLNAEQKVQLVQYHILPSFISISSFQTLSNPVRTQASNTDEYPLNITTQGNWVNISTGVVNTTISGTIYADSQLAIYKIDKVLLPMAIFAPTKLHKALAPAPALLAKPTKGLAGSGKGSDSSSDPSSSTSTLDDNPVSSMHASSAFRNGMVATIGVINVFGAIAILF |
| CsaFLA16 | MNTKHFVTIFSFLTLIFFHATTLATTPTAHAPSQSPAQAPAKPLLAQPPKKSKSSSGAPALDSAASSAPPLSQEPIVQAPPHKGRSRIPTDVAGILEKVGGFSVFNRLLKSTEVLTQIENQLSASNSLTILAPTNDAFASSLKPGTLNTLTKEQKIQMIQYHVLPTFISLSNFQTLSNPVRTQAANTYDYPMNITTEGNWVNISTGIVNASITATVFSDDQLAIYRVDKVLLPLGVFAPRPKLQPSPSPSALLAKPTKDSSSNSSSSSSSSSMSSRADGPGGASEDNDDDQKTNNASSASDFTIGARTMSFGAVIVSMVAIKYILVLF |
| CsaFLA17 | MDTHGYGVSFLLFFILCSFTNSFAALPNNPSQKSNSTSSGSGQINSNSVLVALLDSHYTELAELVEKALLLQTLEEAVGKHNITIFAPRNEALERQLDPEFKRFLLEPGNVRSLQTLLMFHIIPKRIGSGEWPASDSVPVRHKTLWNDRVHLTSKNSGEKVIDSAEIVRPDDVVRPDGVIHGIESLLIPRSVQEDFNRRRSLRSISAVLPEGAPVVDPRTHRLKKPVAPVPAGAPPVLPIYDALAPGPSLAPAPAPGPGGPRGHFDGMAQVKDFIQTLVHYGGYNEMADILVNLTSLATEMGRLVSEGYVITVLAPNDEAMAKLTTDQLSEPGAPEQIVYYHIIPEYQTEESMYNAVRRFGKVRYDTLRLPHKVMAQEADGSVKFGHGDSQAYLFDPDIYTDGRISVQGIDGVLFPPNEDPNSEKKTTPLVKVVTKPTRRGKLMEVACNVLGVFGVSSSCQ |
| CsaFLA18 | MNKQAIIYFSFLFLCFFYHCKGATILAHSPAQPPSKHVAAAAPTKAKALTPTKAPTALPVPAVEPPSQVPLVQAPPHKALYTPTDVTKILEKAGIFSVFIRLLKSTSVSIQIENQLNVSNTLTIFAPTNGAFGALKPGTLNTLSNEDKVQLVQYHILPSLVSLQNFETLSNPVRTQASNTNDFPLNVTVEGSSVNISTGIVNATISGTVYEDNQLAIYKVDKVLLPLGIFGPKPKTKQHLAPSPTPLKPSKDTNVSLPSSSTEESISSDVDEGDKSSKSKAAVLMNNGVVNIGVVMIVVITMWGHF |
| CsaFLA19 | MAKNQILITISCLILFLSLFHTLTLAQLSSPAQAPSTTPNKKNIHPSSHQSPAQSPDQPLVEAPPTAISRRKGHPTNITEILEKAGDFSLFIRLLTNTDVITPIENDLNSSNTVTIFAPTNGAFSALKTGTLNTLTPQQKTQLVQFHIISAFIPLQNFQTLTNPVHTRAINTRDFPLNITSTDGSSVNISTGVVNTTISGTVYSDNQLAIYRVNNVLLPMKIFAPKKLMSSLAPSPAPAVALKPKKKSMASAESSESPSSSLKPSSTSTSSSSPTSSSSSSNDDETVATSTNTSGAERGPVFATISILVAVVALFL |
| CsaFLA20 | MAKSLFFFFFILSIFSSSALGSCLTLLNAAEILSNSGYLSMSLTLKIASQTIKHDSPTATVFAPADQAFVKSGQPSLFLLRRHVSPVKLSLETLKTLPRGSVIPTMVPDHPLIVTASLSGDGYISINDVRINEKAVFGDGFVALYGIDKFIDSSFFRADQPPSPAPAPAPAPFHGKTDSFASVAEFLRSRGYYIMATLLDAQLTGLGDGTKLTIFAPVDRTFDYYASNISDYALIFRRHVVPRLMTWQDLIGSQVVGTKLPTFSRGFMIEVRSSDGIPMLNDAPVVFRDIYRSQRLIVHGLNGFLKPFTDQEWNQDSFSNGFVGDDSHG |
| CsaFLA21 | MAHCCTGSWRAPVYFTVSVILAFIAISTSMHSKTEHPSSPTKLNFHELSLNASKALRKAGFNVMATLLQVSPEIFLLSPNSTIFAIQDDAISNSSLPPWLLRNLLQYHTSPLNLPMKDLLKKPRGSCLPTLHRQKNIAITNIDFKETTVDINNVSVTHPDVFLAETISVHGVLEPFSSLDPEDVHQGWNSIQAPTCNAMSVLVSDAVKSTNMVEWSWIVRLLSSNGFVPFAIGLNSVLEEILKDYKGLNSVTIFAPPNLQSLTSPSPLLKRTVWFHILPQRLTYKELTALPAGTLLMTLVRDLSLEVAGTAGFKGGLIINGIEIVAPDIFTSKKFTVHGISRAFEVADQVAAIGT |
| CsaFLA23 | MAASLLISLTLLSFLSLSSSLPSNTIIDASEILSDSGFASMALTLDLVSQTLTQRTPSLTIFAPADDAFKRSGQPALSLLRYHFCPLTLPLETLKSLPSGTKIPTLLPGRTLTVTHSSSTSEISLNNVKISRRFPIFDDGSLIVFGVPEFFDPNFQAPGPGNSPRFGPRCKSLPSKAAAMGFPGASWFKEASRDLRSNGYSSMASFLDLQLLGFNKDPTTMTVFAPNDMAMANRPTDQAQDPSIFLRHVVPCKLLWSDLINFTDGTVLPTYSDGFTITITRSGSTLMINGIPVTVSNLHYSDSVVVHGLNELLTGQATTSGSG |
| CsaFLA24 | MKMNRGRFLKTSISFVWLVVLFGCLFVVLISVLRLPEVSNSRKAIGLYHNTKTRESSESSSIGKFGEKMIEMLPEDLAFTVFVPSERAFERDLRLRKNESLVEKMNDDTYAVISRVLGFSAVPRTIITDDVSSGEEILYDSISGFVLYISKDVDGMLVVNRVRSEKVDIKRNKIVVHVMDGVIMDAEFEESVQPDDEDEEK |

**Additional file 5: Table S4.** **CsaFLAs from *Cannabis sativa.*** FAS domains are in turquoise, AGP domains are in red, signal peptide are in green and GPI anchors are in purple (the color-code is as after [7]).
