# Supplementary material for: Identification of fasciclin-like arabinogalactan proteins in textile hemp (Cannabis sativa L.): in silico analyses and gene expression patterns in different tissues
Source: BMC Genomics. 2017 Sep 20;18:741. doi: 10.1186/s12864-017-3970-5 (PMC5606014; doi:10.1186/s12864-017-3970-5)
Supplement: Supplementary file 10 — Promoter sequences of a CsaFLA subset. (DOCX 18 kb) [file 12864_2017_3970_MOESM10_ESM.docx]

Promoters of the *CsaFLA*s upregulated at the top:

>*CsaFLA4*

CACTTGACAAGTGATTAAAGTATGAACAATAGTCTCTTGGTCACCATTACAAATAGGACAGATAGCACTTACCTCTACCCTTTTGGTTCGCAACATAACAGTGGTAGGAAGACAATTCATGGCCGCTCTCCATACAGTATTTTTGGCTTT

CGGTGGAACTTTAAGCTTCCATAAACTCTCCCAGAACGGTGAATCTTCAGTACTATGCCATTGTCCTTGACTTTCCTGAAGCAAATTGTAAGCACTTCGAACCGTGTAACCGCCAGATACATCTTTGTGCCAAGTGAGATGGTCTTCCGT

CGAAGACAAGTTAATTGGTATAGCAAGAATAAGTCTCCTATCCCTCTCCAGGAATAAATCATTTAACAGCTCCAAATCCCAACCTCCACCTTCTTCATGCATAAGATTCGACACTTTTGCATTCACAAGTGCCGGGTTATCAGAGATGAT

ACACGGATTTGAGTGATCTTACAACCAAGGCTCCCCCAAAACTGAAATATTCTCCCCCATCCCTATACACCACCGAACCCCATAGCCACCAAAGACTGAGCTTCTAGTACACTTCGCCACACAAAACTTGGGTTGTTGCCTAATTCCGCC

GAAAGATATGACCCGTTTGGATAGTATCGAGCCTTGAAAACCCGAGCCACCAAAGAGTTTGACCGAGAGTATCCCATTAGAATGTCATTCCAATACTTAAAATACAATCAAACATAGGAATTGAATGAAAATTGTTTTCTTTCTATTCTA

TTCTATTTCATTACTTCCAACCAAACGTTACCTAAATAGTAAATATATACAATTTTAAAATACAAAATATTAAATAAACATTATTTAAAATACAAACTTATATTTAAATAAAATACAAAATATGAAATGAGTAGACACTCTCATTTATAT

TTATTATTTGTGGACATTATTATTATACATTCGAACATCTTCAATTATTCCGAAAATACCCTTTGTCTTAATCTTCGTTTTTATACAACTCACTCTCTCACTCACTAACAGAAGAGAGAGAAAAAGAGTGTTTGAGAGAGAAACAACA

>*CsaFLA23*

CTAAACTTTGAACAAGGAAAATGCAAAAATTTTATAAAAATTGATATTTATGTAATTATTCCAATATTATTATCT

TTTAGGCTATAACAAAAGGAAAACCCTTCTTGTTTTTGTTTTATTTACATATTTTTTATTCAAAAAATAAAAATTTAAAACATTTTTATTTTTTTTTTTGGCTAATTAGGATTTTTACTCTCCGAACTTTAACATGTACCAAATCATGCT

CGTGAACTTTTAAGTTAAAAAATACAAATTTTTGTATCTATTAATCAAAAATACCCTCATATTATATTTTTTTTTAAAAAAAATATACCCATTTTTTGAGTAGATTGCCCAATATACCCATACTGTTTACTTAAGTCTTGCATATAATTT

ACATTAACTTAAGGGGTCTAATGGAGACATCATCCATAAAAAGTGGGTATATCTTAAAGGATATAAAAATGGAGGTAAAAATTAAAACAAGTAAAGTGGGTATACTAATTTTCTCTTAAAAATATCCCCTAAACTATTAAGATTGTTAGA

TTTAATGACATTTGTCTAATTTTAGTAAAAAAAAATCTATAAAATGAAAGTTGAGGTGCATGTTTGATATGTGTCAAAATTCGAGGAGTATGATTTGGTAGATATCAAACTTTAGGATCATGATTTAATACATAAATAATGACTGAAATA

GTAAAATTGAATGAAAGTACACAAAAATCTTTAAATCTACCCTCATTTGAAACCATAAGGAAAAGGGGCAATACCGTCATTTGAAAGTACATTCCTCTCTCCCCAAATCAACGAACACAAACTAATCAAATCTTTCCCGCCAAATAAGAA

ACTTCTCTCTTCTACAAATAAATTCTTCTTCCTAGCTCTGTCACTATCA

>*CsaFLA7*

ATATAATCTCACCCCACTTCCACCTTTTATTGCATCAGCTTCTCATTTCTTCACTGCCATTTCTCTCTGACCTCCTCTTGCTCTCAGTCTGAGGTGGGTTTCCTTAACACTCTGTTCTGCTCTTCTCATTGTACCTTCTTTTCTCAGATTTCTTTAGCTACATTTGTTTGTTTGTAATTTTTTTTTTTAAATGTTTCCATTTTGAGAATTCTTAATGCTTTCTTGCTTTCTTTTGTTTAATCTCACAATGTGTTTTACTTTTAGTAAGATCTGATCTAGATCTGCTTCTTTGCCATTATTGATGTACATTGTGTGAATGTGGGAATTGTTTGTGGCACTTCGACGGATTTGGTGAAATGGGTTTCACTAGATGTTGTTAGTGAGCTGAAAAGTAAGGTTCTTCAAAATGATTTAGATGTTTTCAGTTTAAAGGCCATTTTGATTTGGCTCTTCTTCTCTTCAAATTTTGAGCGTCCTCTACTGAGTTGGATCTCTCATTGAACAGTTTAAAGTATACTGTATCATATTTGAAAGATGAGAGAATTACATGTGCAGTGTGTCATTGCAGCTGTTCTTAATCGGCTCTTCTTCTCTTCAAATTTTGAGCGTCCTCTACTGAGATGGATCTCTCATTGAACAGTTTAAAGTATACTGTATCATATTTGAAAGATGAGAGAATTACATGTGCAGTGTGTCATTGCAGCTGTTCTTAATTTATTATATTCCACTAGATCTTCTTTCCCCACCTAACAATTTCATCTTATTAGAAAATGGGTATGTATAGTTGACCAAGATCTAGTTCTTCAAATCAGTAGTACTCACTATGTAGTGTTAAAAAACAGGTTCCATTTCGAAATAAAACATAGAATGAATGATAACCCACATTATGATTTGACCGGAGCATAACACTAATTCCCTTCTTTTATTAATACTGAGATCTGAATTGGGATATGAAATTGTTGTTTTTATAGGAATTTGGAGAGTCTGGTATTTCTCACAATAAGCACTTTTCAAAGACAGTTTGTCCGAA

>*CsaFLA1*

TTCTTCTTCTTCTTCTCACTCTCTCTTTCACTGAAACTCACCTATATATAAACACCTCTTCTCTTTCACTTCTCACTCCTCTCTCTTTCTCTCTCTAAAAACTCCTTTCATTTTCTCTCTCTAAATTCTCAGACACA

>*CsaFLA8*

CTTTACTTTCTCTCTCTACAACTTCTCTCTCTCTCTCTTCTCATTTTCTCTCTCTTCTCTTCTATCAGTGTCTGAAACTCGCCGCATTAATCTTGGGTCTGCCGACGGAAGAAGCC

>*CsaFLA10*

TGCATTGATAAGTGGAGGCTAATGCCTAATGGCGTCCAATGCACGGCCCCAACCAAACGGATAAACGACTAGTTAGTACTTTTTCTTGAGAAAGAAAATTTTGGTTTTTGAACTTCAAACTAAGTTCAACTATTCCTTTTAAAAAAAAAAAAAAAACTAAGTTTAACTAATTCTTATTAAAAATTATTATCATAATAGTTCTTATTTTGGATAAAAACTAACTTAACCATACATGTGAGTATGTGACCCTTTGTTGTGGTAACTAACTGGTCCATTCATCTCTTTCACCCTCAACCCCAATGGTTTAGAACCGTTACATTACAAAAGAGTCATTTCCATCTTTTTTGCAATAATTCAAAAAGTCGAGGACATTTCTGTCATTTCATCCAAAAGAAAAAAAACCATAAAGAAAGCCAACAGCATATAACTCCTTTTCCAGCTCTGACACCCACTCCCTCTCTCTCTATTTATTTATCTCTTTGCCTT

>*CsaFLA20*

ACATGCACTAAATATTTTATGAGTTTTTACATAATTTATACAAAATTATGGCTTTTATCAATATAGTCGTTTTATGGGAAAGATTATGTAAAAAGAGTTCGGAGAATAAATCCTAAACGACAAAATTAATTGTTCTTTTTATTCACTTTC

AAACGACATGTCGTAGATTCTCAACAAATTTTACGGAACTGAACATTTTGGCAACCCTTATCCCACCCATAAAATTTTCCTATATTTTAATAACTACTGAATAACTTTTGTTCAATTTATTTTTCTATCTATAATGGAGCTGTCAAAACA

TTATTCATTATCACAATGATTCATATTAAAAAGAAAGATTAAGAAAAGTTCTATAATGCACCTCATTAAAAGGAGTATACCGATACATCTCTATCCGTTTTAGTATCTAAAATAATTTTTAAGCGGGTGTATTGTAAATTATCTTATAAC

TTTTAGGAAAATTCTATAATATACCCTTTAAAAGGATGTACCGATACATCTCTATCTATTTTAGTATCTGAAGTAATTTTTTAATCAAAATTTTTCTCATGGTTATTTAAGACATCCAGCTAAATTTTAAGAAATTCAGAAAAGTTTAAC

ATACCAAAAACTACCTTCAAACAGTGCGTTACATGCGTGACTATTTTATTTTATAGTCGTGTAAAATAGACTGTTTAAACATTGTTTTTTATATTGTAAATTATTCCAAATTTCTTAAAATTTTGTAAGATATGTTAAATAGATATAAAC

TACATGAATATGAAAAAAAATTAGACTAAAAAATTTTTTTGATGCCGAAATAAGTAGAAAGTGCATCAGTACATCTCTTTTAAAGGAGTGTATTGTAAAAACACTTTTATAGTCTATCTCAAATTTTAATTAATTATATTTACTAGAAAA

AATAAAACGACACGTCGTAGATTATCAACGAACTTTACCAACTGAATTGTTTGGCGCCCAACAATCAAAGCTTTAAGTTTAAACTCTGTAAAAGACATCTTCTTCGACTAAGGCAAAGAAAGCCAAGAACCATTCTCTCTCTCCCACTAC

C

Promoters of the *CsaFLA*s upregulated at the snap point:

>*CsaFLA2*

GTGTCTTTCGAGCTAGTCTATTCATGCTTATGTGGCGTGCTTTTTATGCTTCTTGTGTTGTGTCGTGCTTAAGCATATATTTTTTGTGTTATATCGTATGCCAATTTGTAGCATCATGTCGTGTACCCAAACTCATTTTTTTTTTTGTGCCTAGTCATACTCGTGATTTTCAAATTCTTACAATTTTATGTGGTGTCAAAAAACTCAAACCCTATTTACTAGCATTGGTGAAATGTAAAGACAGAAACAAATAAATTAACAAACTAATTAAAACAAAACAAAACTAATGTAGCATACTGATCATTTTATTTGTTTTATGTACTTATTCTTATTTTGGGTCAGATTAATGCTAATTACTACTAATATATACACATAAAATTACTCAGTTTTATTACAAATAGATTATTAATACCCAATAATGTAATAGAAAAGGGACCCTCAAAGACATGCCCACATCATCTCCACCCAAATCCAAGCTGTCATAAACCAAAAGCAATTCCCATTATTAGGACTACAAATAAGACCACGACCCGCTCTTATGATCTTCACCATTAACATTTATATACCCACTAAAAACTACTAAGCACCCAGTACTGTTCGTCAAA

>*CsaFLA6*

CGTAGCTAAAGGTGGTTGTCGTCCAGTAAAGAAGAAGTCGAAGGTGTCTTTGCCTGAAAAAAATTCCAACCCCGACGGCCAGAAGGGAAAGAAGAAGTCAACTTCAAGACAACTGTGATGAAACTGTAATGCAATAGTTATATTTGTTGCGAGCCAACTAACATAAAAGCACAAAATAACTAATAATTCCACATATTTAATACAAACAAATATTACAACAATCATGTTTAATCTGTTCAAGTCGCGTATTTGACAATTCTGGGTCTTATTTTTATAATACTATGATATTGTAGTAGCAAAATCATTATTCAACTAACGTGCAACTTAATTGTAACTAGAAATGTTAAATGTGAATGTGTATGTGTATCTATAAAAATCTATTTTTCAGTACTTTTTTTTGTCACGATCGATAATTAAATGTACCTTAATAGTAATATGTAGAGAAATAACTGTTAGACCTTTATAAATTAATTTTATATCAACTAATTTAGTATATTAATAAAACTAAGTATGTTATAATAATTTCTTGAAAGAGCTTTGTATTGTATTGACAATATGAAACATTTTATCCACTCATGTGGCAGCAATCACACGTCAAGATCCATCAGCAACTACAATTATCACTATGGTTGTTGCTCCAGAACCACCAACATTTAATGGATGTCTCTCTGTTCCAGCTACCGCTATTTTTTTTTAGTTCATGTTCCAGGTCTATGCCTCCTATTTCCCCTGCTACTACATCTGGTTTTGTTCTACCCTCATATCCCACAAATCACCAACTATCTACACTATCCTCTCCTACATCATGTCAAGAGCCACACCTTTAAACTCAGCAACCACAAAATTTTACCCTACAAACACCCAACGCAGTACCCACCATTAACTCTCATTCAGTGCAAACAAGATCTCAATTTGGAATATCAAAACCAAAAACTTACAATGCAACTACATATATACTCTTCCAGAATCTCTCTTGCCTAATGAATCACAATCTCTGAAGCAAGCTCTTGCTAATCCTAAGCGGTTTTCTTCAATCACTCATGTATATATATAA

>*CsaFLA24*

AATAGAAATAAAAGTAAAATTAAAACATGTAAAATAAAATGTATACAATCTAATTTTTTTCGGGGTAAGTTGAATACTTTTATTAATCAATTAATAAAATTTACCTTTAATTTTATATTTAATTGAATCATATATCTTTTCAAATGTATTGTAACCCAAAATACCCTGACATAAGAGAGTCACATGGACAGTAGTTTGAAGTGACAGAGGAAAAACTAGTATAATATTAAAAAAAAGAAAAAAAAAATAGTAAAAATAGTAGTTTAAAAAAAAAAGGTAAAAATAAAAAAAGACAATATTTATAATTTCTTTTTTTTTTTTTTCAACATATTACATAAAAGGCCCAATAGTCAATCGGGACCCAGTATTGGTCAGACTGGGCTTATTGGAGAAGTGCCTATAATTGGTTCAATAGAAAAAGAGTACAGCGTTCAACCTCAAATCAAATCACAGAGAGCTGAACCTGAGTTGTCTCTCTCCCAAGTCCCGAGTCCAATAGAAAATGTCAGTGGTTTATTGTCACTTGTCAGATCTATCCACCGGAAATCGGAAATGAATTTACTAAGTTTCCGAATGAGTAAGGTATTTTCAATTTCCTACATTAATGTGTTTTTCTTCTTTCTGATTCAATTTTCCCAGGAACCAAACGTACATGGCTATTGATTTGTAGGAATGATGCAGGCAGCTCGGGAATTACTCACTCCTCAACAATTAACTGAAAAGGTTTCATTTTGCATGATTGGCGTAATGGGTTTTCTTAGAGCCAGCTTGTTTGTTAAACATCGATGCCTGTGGTGGCTATGAGGGAGTTCGTGTACGATTTTGTTCTTGCTTTCGGTCAAGCTCACGATCATTTTTCATCAATAATTTAAAGATTTGGGGGAAGTTTTGAAAACGCGCTTTTGGATTTTAGATTTACTTATAAAAAGCAAGTTTTTGACACTACATCTAACTTTTACAGTAGAACTAGTGAAATTTCACTTCATTTAGTTGCAAATCATTTGCTTTCAAAATAATCGGATAATT

Promoters of the *CsaFLA*s upregulated at the bottom:

>*CsaFLA3*

TTTTAATAATTCTCTTATAATAATATATATAAGTTTAGCTCCCAAGTTCCTCAAATGAAAACTTCGTTTAAATTTCCCATGCAAACCAAAAGTCACTTTTTAAAAAGAGATCATATAAAAACTTATCTCTTTTCACAATTACGAAGACAAGAGACATTATAACTACGACGTCGTTTTTAAATATAACCAATAAATACATTAAAAAAACTACAACCTTTCACCTAATTCCTTTTCTCTCATCAAAAATACCACAAAATTATATGAGCAAATATTTACATATATATAAAGCCCTTTCCTCCCTCCAAACATATTAATTATTATGTATTGAATCATCATCATCATCATAACCCTAGCTAATAATCTCAAAGAAAACAAAAAAAAAA

>*CsaFLA16*

AATGAGGCATCTGAAGCTGTTTCCGCTGGAGAATTTGAAGTCAGTGATGCAAGTATGTTTTTGTTTGCTTTATTTTTGCTCTTAACAAGAAACATGTAATATTGTATGCCTCTATGTATGCTTTTATGGTTTGTAGTTTATTATGCTATTGTTTTTTCAGATAGATGATTTAGTTTTATGACTGAAATATTCTACCTACGTGTATTGAACTTTTCTGCAGTGTCTGAAGCTGGAATTATTATATTACCTCATCCAGAGGATGCAAATGGAGGAGTGTCTTTGGAGGACAAAGATACATCTGAACCAGAACAAGCTCCTTCGAAATGGCCAAAAAAACCCGTAAATCAGCATTCTGATGTGTTTAATCGCGAGGATTCTTGGTTTGATACTCCACCTGAGGGGTTTAGTTTATCAGTAACTTCTTAAAACTCTTATTATTTGGTGCATGAATTTATCCTTCATACCATAACATATAACATCACACTAATTTGATGGGAGCTATCTATCTTTCCATAACATGTACCAATCGTACAGTACTGAACTATTATATCTGTTTCTTTCTTTTTCATTAAATATGACATAATAGCCAAACAAAAAAGTGCATGCATGACATAAAATTTAATTACGCAAATTAAATTATTTTCTCTCTATAAATATATAGTTAATAATAATTAAAATATAAAGTATGTAGTACTGTACCACCTAATTATTGATTGATAGTACTATTCTCTACCTTATTATTTAACACTTATAAAATCCTCACTTCAATCCCAAACCAAAGCACCAAACACAACTCCTCTAAAAAAAACACCAATATATTGAATCTAAACATAACAACACAATAGCCATAATAATCATC

>*CsaFLA13*

CAACTATTCATAAAATAGGCATTTGTCTCTGCAATATAGGCAAATTTCAACAACCTCTCCCAATTTAGCTTTTATGTTGATTATAGTAATAAGACATTTCAGTTCCATAAAAATATATGCGGTAATGCATATTAATATATAATCACCTTAACGGCTAAAAAAAAATGATACCTAACAATTTATTTTTTATTATCAATTTTGTATTTACAAACTTCATTTACTATACACACAATTACACTCATGTTTATTATTTTAATGAATTTTTATGATTGTTTTTTATTAAATTCTCTTCAAGAAATTATTTTTTTTTCTTTTAAAAATATATATGTAATGAGTATCTAATAAAAATCTTTACCCGTTAGGTAATCCTCACTCCGTCCCGTTTTAATTTAAAAAGGTATATATGGAATAAATTTAGAAAGTAAGTATTAGGGCAGGGAGCAATATCCCTGCACCCACTCCGCCCGTTTACATCCCTATCCTTATAAGCTTTAATTCCTCAATAATGAGATGAAAACAAGTGTAGTACTTATTAATTACATATACACAATAATTATTATTTATTTTTCTTTGGATATATTTTTAATATATAGGTTAGATGTCTTTTCTTAAGCTAATCTATATCTAGGGCGAGGTTCATATATATATATTTATATACATGAGTCATCATAATCTAAATCGAACACCCAACTCACATATATATCTTTGATATATATTATATATCATAAAGTTGGGCTATCTCCCACAACGTCACAAACCATCATTATCAATCTTAATTTTTGCTCTTCCATTAGTGTCAAAAACAAACATTGCATATCTATCTTAGAAAACTATGTATTACGTACCATATAGTTTAATTTGTTAAACTTATAATCACTATACATGCACCCTTTAACCTAATATTACTATACCTATAAAAACCCTTCTTCCCCTTCTTCCAACTCCATCATCAAAAACAATTATATCATCAAATCCCACACGTCTTAAACAACCCAAGAAAAAAGAAAAGAAAAGAAAATTTCACTACAACAAA

>*CsaFLA12*

TTTTTTTCACATAACTAACAATAAAATAAATAAATTATTATTATTTTATGTCTCTAATAATGAATTAAAATAAATAAAATAAAAATAACAAATTCAAATAAAAAAATAATTGTATGTATAAATAATTGTTTAATTTTATTTAAATTGTAT

GTAAAAAAAAATTATATATAAGAATGGAATATATATTTGATTTATTAAGTTTTGAAAAATATAATTGTATTACATGTATTAGACATTTAAATTAATATTTTCTTTACATTAAAATGTTATTTGTATATATATATAATTTTTTTTATTTTT

TTTATAAATATGTGTGGATTGGATTGAATCGGTTACTTTTACATGTCAATTAACATTCAATCTGCACAAGTACGGATATCCGCACTTTGCAGATTAAATTAGAATAGATCGTGCAAATTAGATTAGATCAACTACTGTACAGATCGTCTC

TCATATAACTAAATAATGTGATATTACACCCTTTCATTTAATTTATTCATAGTATCAATAATTTTGCAATAATTAAACTTTATTTCTCTCCCAACATATGTAATATATAAACCCCCTATCTCTCATATCTTAATTAAATAAACATTCAAG

AAAAACTCATATAATTATTTCATAATAAAACTTCTTTCTAGAGAAGAAGTGAGCTACACCACTCAAATACCTACAAATAAATAATTAAAAAAAAATAACC

>*CsaFLA15*

TTTAACATTTCTCTATATTTTATGATCATATATATCACTCTCACTTCAATATTATTTAACGTACCTTTCCAACTCAAAAGTAGATATCATCAACACATTATAATCCAACTAGCTAGCCACCTTAATATTTGGTACTTCCCACAACTAAAT

AATAATAACAATAATCATTATAACCACCTCATGATTTAATTAATCTCCCTTCAAACCATATTAACTTATATAAAACCCCAATATTCCTCCCATTCACCGTATCATCAACTCACAAAATCTCTCAACATAATAATAATAAAAAATAATAAT

AATAATAACTAAATAATTAAACCCTAGCTATATACAAAG

>*CsaFLA18*

CGAGATTAAACCAATCCCTCTAACTGGTTTACACTAGTTTTCCATAACAGTGTGCTTAAAGTCATTGTGATCAGCCCAAAAGTTGTAGAATTTAAACGGTTAAACTCCTATATTCTCCATAATAGCTGAGGAAACAATAATAACACAATGATCCCATATAGTCTCCCAACAATAAGAAGTAATCAAGTTAAGAAATATGTCTAACCCAAGAGCAAGCCAATTATTATTGGAGTCCTCCAGATTTGTAGTAGAGATAAAGCCCCTACCTACTCTATCATCATAGCTGAAAATTGTATTAAAATCCCCCAATATCAACCAAAACTTAACTGGAAATTTAAGGCTTATTTTATATTATTTGGGACTTCAAAATTTTAATAAAAAGGATAGAAATGATTATAATAAAATATAAAATAATACATAATTATATTAAAATAAGTTTTAGTATTGTAATCATGTATAAAAGCCGAAAAAATTTTATTTAGGGATAATTCTCAAATGATTTAGAGCAACCAATCCATATTTTAGGCAAGGTGACATCTTTTTTGGACTCTAATATATGTAAATTGTAATCTAAGTTGTAAAATATTAAAAAATGAGTAATTTGCAACACAAATTTTTAAGTTTAATAAATAAATATCTAAATTTAATTTTTGGCCGTAATAATATTACCTAAATTATAATTTTAAAATTTTTGTAAGTACCTAACTGTGAAATACTAAATAAATTGGCAATTGGCACGTGTTAATTCTTGATTGATCCAAATTATTAAATTATAATTTACAAAAATGTTTAAATATACCAATAAAAAAATGATATGTGGATAATAACTTAATATTTACCGGCCAGATACTTAGAGAGTTTTAGAATTATAATATAAGTATTATTACCACTAAAAATTAAACTTAAATATTTATTTGCAACCGAAAATTAAATTTAAATATTTAAATCATAAATTATTCTTAAAAAATTAGCATCTCAATCAATAAGGTTTACGCTCCCTTATCATGATAAACACTTAATTGGATGACATGTCTATTATATAAATATATATTATAAAAG

>*CsaFLA19*

GTTAATCGTAAATATTATTTAATTTTTTGGTTTAATTATTGGGTTTATTTTTTGAAATTTGAATTTAGCCGTGTAAAAAAATTTCAAAAATAGTTGTTAATTGGGTTTAATTATTGGATTTATTTATTTGTTAACGTTTAACGTTAACAGTCTGTTAAGTTAATCGTGTAAGGCTAAATAAAAAAAAGAATCGTTAAACCAAGAATCGTTAAACCAAGAATTGCCGTTAGATAGGCACTTTTTTAATATATAAAGATATACTCAATTTCACCGAATATATATGAGATGATTTCTACTTAAATCAATTATTATCTCACATAATGATCATGTCTTATATATGTCTACAGATTTTTATTATATGCATGCACACTATTATATGAAACGAATGGATTAGATTTAGAACATAGCGTCTGTATAATTGGTAGGACAAATGCAAATAATTGTGATCTAGCTATATATGAAAGTGATTTCATGATCATATATATACAATAAATAATAATATGATCATGAAATTGTTAAAATGATTATTGATAGCATCACATTTGTTCGATAGTTCCCTCTGATCTCTTTCTTTTATTAGTACTACTCACTGCATGGACACTCGAACTCTTATATATAATTAATGTGACATTACACCCTTTAATTTAACTTATTATTGCAAAACTTTACTTCTCTCTCAACCAACATATAATGAAGTAAACTATATAGTGAAATAATATATAAATACTCACATCTCTCAACTTAAATTCATTCAAGTTTAACTACATTAATAACCATTTTCAAACCAATTTTCCATCAACATAATTTTTTTCTAGCTAAGAAATCCAACCTATATCAATTAATAAAAATTAATAACC
