# Supplementary material for: Identification of fasciclin-like arabinogalactan proteins in textile hemp (Cannabis sativa L.): in silico analyses and gene expression patterns in different tissues
Source: BMC Genomics. 2017 Sep 20;18:741. doi: 10.1186/s12864-017-3970-5 (PMC5606014; doi:10.1186/s12864-017-3970-5)
Supplement: Supplementary file 11 — Alignment of the promoters amplified from Santhica 27 with those from Finola and Purple Kush. (DOCX 18 kb) [file 12864_2017_3970_MOESM11_ESM.docx]

**Additional file 11: Alignment of the promoters amplified from Santhica 27 with those from Finola and Purple Kush.** At The Cannabis genome Browser, for the promoter of *CsaFLA7*, the Finola sequence has the coordinates 14556424:3018-3911, the Purple Kush has the coordinates scaffold71750:1289-2318. For the promoter of *CsaFLA16*, the Finola sequence upstream the start ATG could not be retrieved; the Purple Kush sequence has the coordinates scaffold4787:3046-4047. The conserved SOC1 and MYB3 motifs in the promoters of *CsaFLA*7 and *CsaFLA16* from Santhica 27 are highlighted in yellow.

*CsaFLA7* promoter

PurpleKush ATGATTATTTTATTTTATTATGGTAGTGGAGTATTGTATTGCCTTTAAAGTTTAGAGTGA

Finola ATGATTATTTTATTTTATTATGGTAGTGGAGTATTGTATTGCCTTTAAAGTTTAGAGTGA

Santhica ATGATTATTTTATTTTATTATGGTAGTGGAGTATTGTATTGCCTTTAAAGTTTAGAGTGA

************************************************************

PurpleKush GTGACTCATCTCATCCTTTTCCTACCCTAAAACTCACAAAGCACTTCCATGAGTTTCATC

Finola GTGACTCATCTCATCCTTTTCCTACCCTAAAACTCACAAAGCACTTCCATGAGTTTCATC

Santhica GTGACTCATCTCATCCTTTTCCTACCCTAAAACTCACAAAGCACTTCCATGAGTTTCATC

************************************************************

PurpleKush AACCAAATTGAAATTGGTGAGAGTTTCTCACGAGTCACTCATGACAAGAGAATTTGAATA

Finola AACCAAATTGAAATTGGTGAGAGTTTCTCACGAGTCACTCATGACAAGAGAATTTGAATA

Santhica AACCAAATTGAAATTGGTGAGAGTTTCTCACGAGTCACTCATGACAAGAGAATTTGAATA

************************************************************

PurpleKush TAAAATGGAGATCTTGCCATTTGGTAGGGTGGAGTGCAAGATTGGTGTTTAAAATGTTTA

Finola TAAAATGGAGATCTTGCCATTTGGTAGGGTGGAGTGCAAGATTGGTGTTTAAAATGTTTA

Santhica TAAAATGGAGATCTTGCCATTTGGTAGGGTGGAGTGCAAGATTGGTGTTTAAAATGTTTA

************************************************************

PurpleKush TTAAACTAATAAGTTGCAGGTGATTTAGAAAGCCCACCCTACACTAAGTTTGTTTCTCAA

Finola TTAAACTAATAAGTTGCAGGTGATTTAGAAAGCCCACCCTACACTAAAATTGTTTCTCAA

Santhica TTAAACTAATAAGTTGCAGGTGATTTAGAAAGCCCACCCTACACTAAGTTTGTTTCTCAA

***********************************************.:***********

PurpleKush ATGGCCTCTTTTCTTTTAGAAACTCATTCCAATTAATTATTACTTTATTTTGTGTCCAAC

Finola ATGGCCTCTTTTCTTTTAGAAACTCATTCCAATTAATTATTACTTTATTTTGTGTCCAAC

Santhica ATGGCCTCTTTTCTTTTAGAAACTCATTCCAATTAATTATTACTTTATTTTGTGTCCAAC

************************************************************

PurpleKush TAAGAAAGAAAGAAACCACCTACACCTACTCAAGACTTTATATGTCTTTTACCACACACA

Finola TAAGAAAGAAAGAAACCACCTACACCTACTCAAGACTTTATATGTCTTTTACCACACACA

Santhica TAAGAAAGAAAGAAACCACCTACACCTACTCAAGACTTTATATGTCTTTTACCACACACA

************************************************************

PurpleKush AATTGTTTTTACTAAGGTCACCTACTAGCTGTTTTTATTGTGTGTTTTTGTTTTTATCTA

Finola AATTGTTTTTACTAAGGTCACCTACTAGCTGTTTTTATTGTGTGTTTTTGTTTTTATCTA

Santhica AATTGTTTTTACTAAGGTCACCTACTAGCTGTTTTTATTGTGTGTTTTTGTTTTTATCTA

************************************************************

PurpleKush ACTAATAACTCCATCTTTTATATAATCTCACCCCACTTCCACCTTTTATTGCATCAGCTT

Finola ACTAATAACTCCATCTTTTATATAATCTCACCCCACTTCCACCTTTTATTGCATCAGCTT

Santhica ACTAATAACTCCATCTTTTATATAATCTCACCCCACTTCCACCTTTTATTGCATCAGCTT

************************************************************

PurpleKush CTCATTTCTTCACTGCCATTTCTCTCTGACCTCCTCTTGCTCTCAGTCTGAGGTGGGTTT

Finola CTCATTTCTTCACTGCCATTTCTCTCTGACCTCCTCTTGCTCTCAGTCTGAGGTGGGTTT

Santhica CTCATTTCTTCACTGCCATTTCTCTCTGACCTCCTCTTGCTCTCAGTCTGAGGTGGGTTT

************************************************************

PurpleKush CCTTAACACTCTGTTCTGCTCTTCTCATTGTACCTTCTTTTCTCAGATTTCTTTaGCTAC

Finola CCTTAACACTCTGTTCTGCTCTTCTCATTGTACCTTCTTTTCTCAGATTTCTTTaGCTAC

Santhica CCTTAACACTCTGTTCTGCTCTTCTCATTGTACCTTCTTTTCTCAGATTTCTTTTGCTAC

******************************************************:*****

PurpleKush ATTTGTTTGTTTGTAATTTTTTTTTTTAAATGTTTCCATTTTGAGAATTCTTAATGCTTT

Finola ATTTGTTTGTTTGTAATTTTTTTTTTTAAATGTTTCCATTTTGAGAATTCTTAATGCTTT

Santhica ATTTGTTTGTTTGTAATTTTTTTTTTTTAATGTTTCCATTTTGAGAATTCTTAATGCTTT

***************************:********************************

PurpleKush CTTGCTTTCTTTTGTTTAATCTCACAATGTGTTTTACTTTTAGTAAGATCTGATCTAGAT

Finola CTTGCTTTCTTTTGTTTAATCTCACAATGTGTTTTACTTTTAGTAAGATCTGATCTAGAT

Santhica CTTGCTTTCTTTTGTTTAATCTCACAATGTGTTTTACTTTTAGTAAGATCTGATCTAGAT

************************************************************

PurpleKush CTGCTTCTTTGCCATTATTGATGTACATTGTGTGAATGTGGGAATTGTTTGTGGCACTTC

Finola CTGCTTCTTTGCCATTATTGATGTACATTGTGTGAATGTGGGAATTGTTTGTGGCACTTC

Santhica CTGCTTCTTTGCCATTATTGATGTACATTGTGTGAATGTGGGAATTGTTTGTGGCACTTC

************************************************************

PurpleKush GACGGATTTGGTGAAATGGGTTTCACTAGATGTTGTTAGTGAGCTGAAAAGTAAGGTTCT

Finola GACGGATTTGGTGAAATGGGTTTCACTAGATGTTGTTAGTGAGCTGAAAAGTAAGGTTCT

Santhica GACGGATTTGGTGAAATGGGTTTCACTAGATGTTGTTAGTGAGCTGAAAAGTAAGGTTCT

************************************************************

PurpleKush TCAAAATGATTTAGATGTTTTCAGTTTAAAGGCCATTTTGATTTGGCTCTTCTTCTCTTC

Finola TCAAAATGATTTAGATGTTTTCAGTTTAAAGGCCATTTTGATTTGGCTCTTCTTCTCTTC

Santhica TCAAAATGATTTAGATGTTTTCAGTTTAAAGGCCATTTTGATTTGGCTCTTCTTCTCTTC

************************************************************

PurpleKush AAATTTTGAGCGTCCTCTACTGAGTTGGATCTCTCATTGAACAGTTTAAAGTATACTGTA

Finola AAATTTTGAGCGTCCTCTACTGAGTTGGAT-TCTCATTGAACAGTTTAAAGTATACTGTA

Santhica AAATTTTGAGCGTCCTCTACTGAGTTGGATCTCTCATTGAACAGTTTAAAGTATACTGTA

****************************** *****************************

PurpleKush TCATATTTGAAAGATGAGAGAATTACATGTGCAGTGTGTCATTGCAGCTGTTCTTAATCG

Finola TCATATTTGA--------------------------------------------------

Santhica TCATATTTGA--------------------------------------------------

**********

PurpleKush GCTCTTCTTCTCTTCAAATTTTGAGCGTCCTCTACTGAGATGGATCTCTCATTGAACAGT

Finola ------------------------------------------------------------

Santhica ------------------------------------------------------------

PurpleKush TTAAAGTATACTGTATCATATTTGAAAGATGAGAGAATTACATGTGCAGTGTGTCATTGC

Finola -------------------------AAGATGAGAGAATTACATGTGCAGTGTGTCATTGC

Santhica -------------------------AAGATGAGAGAATTACATGTGCAGTGTGTCATTGC

***********************************

PurpleKush AGCTGTTCTTAATTTATTATATTCCACTAGATCTTCTTTCCCCACCTAACAATTTCATCT

Finola AGCTGTTCTTAATTTATTATATTCCACTAGATCTTCTTTCCCCACCTAACAATTTCATCT

Santhica AGCTGTTCTTAATCTATTATATTCCACTAGATCTTCTTTCCCCACCTAACAATTTCATCT

************* **********************************************

PurpleKush TATTAGAAAATGGGTATGTATAGTTGACCAAGATCTAGTTCTTCAAATCAGTAGTACTCA

Finola TATTAGAAAATGGGTATGTATAGTTGACCAAGATCTAGTTCTTCAAATCAGTAGTACTCA

Santhica TATTAGAAAATGGGTATGTATAGTTGACCAAGATCTAGTTCTTCAAATCAGTAGTACTCA

************************************************************

PurpleKush CTATGTAGTGTTAAAAAACAGGTTCCATTTCGAAATAAAACATAGAATGAATGATAACCC

Finola CTATGTAGTGTTAAAAAACAGGTTCCATTTCGAAATAAAACATAGAATGAATGATAACCC

Santhica CTATGTAGTGTTAAAAAACAGGTTCCATTTCGAAATAAAACATAGAATGAATGATAACCC

************************************************************

PurpleKush ACATTATGATTTGACCGGAGCATAACACTAATTCCCTTCTTTTATTAATACTGAGATCTG

Finola ACATTATGATTTGACCGGAGCATAACACTAATTCCCTTCTTTTATTAATACTGAGATCTG

Santhica ACATTATGATTTGACCGGAGCATAACACTAATTCCCTTCTTTTATTAATACTGAGATCTG

************************************************************

PurpleKush AATTGGGATATGAAATTGTTGTTTTTATAGGAATTTGGAGAGTCTGGTATTTCTCACAAT

Finola AATTGGGATATGAAATTGTTGTTTTTATAGGAATTTGGAGAGTCTGGTATTTCTCACAAT

Santhica AATTGGGATATGAAATTGTTGTTTTTATAGGAATTTGGAGAGTCTGGTATTTCTCACAAT

************************************************************

PurpleKush AAGCACTTTTCAAAGACAGTTTGTCCGAA

Finola AAGCACTTTTCAAAGACAGTTTGTCCGAA

Santhica AAGCACTTTTCAAAGACAGTTTGTCCGAA

*****************************

*CsaFLA16* promoter

Santhica ------------------------------------------------------------

PurpleKush ATTTGCTTGCAATGGGTGGCAATTCAATCCAAACACGACCTGTTTATAAATGAATTCACC

Santhica ------------------------------------------------------------

PurpleKush TGACATAATAACTTTTACTTGTTTAATAGGCATTTAACATGTTAATATGTTTATTGACTT

Santhica ------------------------------------------------------------

PurpleKush ATGTAATCAATCATTTAATATGTTTGTGACCTATTTAACCAACTTAATGTCATTTACATA

Santhica ------------------------------------------------------------

PurpleKush ATAAATATGAGTTCATTGTTGTGCCATTTTTGAGTTAGCAAATTATTGGTGTCAACCTGA

Santhica ------------------------------------------------------------

PurpleKush AAATGATCCAAAACTATTAAAAAAAAAAACTCAAATACATTTACTTCGTGTGAGTTTGAG

Santhica ------------------------------------------------------------

PurpleKush TCTTGTTATGTGTCTGACTTATTTTGCTACAATATTATTGCTGGCAAAAGTGAACGTGGA

Santhica ------------------------------------------------------------

PurpleKush TTATATATTGCAGGGTCTAAAGCTAAAAATCCGGTTCTTATCAATGACATGGAGTTCTCG

Santhica ------------------------------------------------------------

PurpleKush AGTACCCTTTTCACAGAAGTTGAATATACCGTGTCCAAAACGCCATTGAGCTCAAAAAAT

Santhica ------------------------------------------------------------

PurpleKush ACCGGTTTTGATATTCAACTAAAAGAACCGGAAGAAAAGTTGGGGAAGAAGAAGACAGAA

Santhica ------------------------------------------------------------

PurpleKush AGTCAGTTTACTTTATTGGACACATCGTTTAATCCCATGAGCAGTGATTTAGAAACGAAA

Santhica ------------------------------------------------------------

PurpleKush ACCAAACAAATGGAAGTGGGGAGTTGTAAAGCTGTTTCAAGAGATGAGCTTGGTATTCAG

Santhica ------------------------------------------------------------

PurpleKush GAGATGCCTTCAACTTCAGCGTCTTCCCAACTTGGCTCGCATTCAAGCTCTGAAAAAGTA

Santhica ------------------------------------------------------------

PurpleKush GGAGAGGTATCTCATAGGACAAAGAAATTGACTGAAGCTACTCTTAAATCGTCTCTGAAA

Santhica ------------------------------------------------------------

PurpleKush GCTTCTCGGAAAAACAAACTTAGTCGCTCTGTTACATGGGCTGATGAGAAATCTGATAGC

Santhica -----------------------------------TGAC--ATGATACGCAGCTATTTAG

PurpleKush TGCAGGAAAAGTAATCTCTGTGAGGTTAGAGAAATTGAAGATAAAAAAGAAGCTAATT--

***. ::.*:*.*.*****:**

Santhica GTGACACTATAGAATACTCAAGCTATG-------------CATCCAACGCGTTGGGAGCT

PurpleKush ------CTATA--TTAGGCAAGAAAGGCAAAGTTCCTCTTAAACCTTC-----GGGGTCA

***** :** ****.:* * .*:**::* ***. *:

Santhica CTCCCATATGGT------------CGACCTGCAGGCGGCCGCGAATTCACTAGTGATTGT

PurpleKush CTGAAAGCTGGTACCTCCATGTCTTGGGCTGATGAAAAGTGCGATACC-----AAAGTGT

** ..* .**** *. ***.:*.... ****:: * :.* ***

Santhica GTAAAGATACTTTTGAGGTTAGAGAAATAAAAGATTTGAATCAAACTTCAGATATGCTTA

PurpleKush GTAAAGATACTTTTGAGGTTAGAGAAATAAAAGATTTGAATCAAACTTCTGATATGCTTA

*************************************************:**********

Santhica GTATGGACATTGGAGATGGCGACTTGTTGCGCCTTGCATCAGCAGAGGCTTGTGTAAATG

PurpleKush GTATGGACATTGGAGATGGTGACTTGTTGCGCCTTGCATCAGCAGAGGCTTGTGTAAATG

******************* ****************************************

Santhica CATTGAATGAGGCATCAGAAGCTGTTTCCGCTGGAGAATTTGAAGTCAGTGATGCAAGTA

PurpleKush CATTGAATGAGGCATCTGAAGCTGTTTCCGCTGGAGAATTTGAAGTCAGTGATGCAAGTA

****************:*******************************************

Santhica TGTTTTTGTTTGCCTTATTTTTGCTCTTAACAAGAAACATGTAATATTGTATGCCTCTAT

PurpleKush TGTTTTTGTTTGCTTTATTTTTGCTCTTAACAAGAAACATGTAATATTGTATGCCTCTAT

************* **********************************************

Santhica GTATGCTTTTATGGTTTGTAGTTTATTATGCTATTGTTTTTTCAGATAGATGATTTAGTT

PurpleKush GTATGCTTTTATGGTTTGTAGTTTATTATGCTATTGTTTTTTCAGATAGATGATTTAGTT

************************************************************

Santhica TTATGACTGAAATATTCTACCTACGTGTATTGAACTTTTCTGCAGTGTCTGAAGCTGGAA

PurpleKush TTATGACTGAAATATTCTACCTACGTGTATTGAACTTTTCTGCAGTGTCTGAAGCTGGAA

************************************************************

Santhica TTATTCTATTACCACATCCAGAGGATGCAAATGGAGGAGAGTCTTTGGTGGACAAAGATA

PurpleKush TTATTATATTACCTCATCCAGAGGATGCAAATGGAGGAGTGTCTTTGGAGGACAAAGATA

*****.*******:*************************:********:***********

Santhica CATCTGAACCAGAACAAGCTCCTTCGAAATGGCCAAAAAAACCCGTAAATCAGCATTCTG

PurpleKush CATCTGAACCAGAACAAGCTCCTTCGAAATGGCCAAAAAAACCCGTAAATCAGCATTCTG

************************************************************

Santhica ATGTGTTTAATCGCGAGGATTCTTGGTTTGATACTCCACCTGAGGGGTTTAGTTTATCAG

PurpleKush ATGTGTTTAATCGCGAGGATTCTTGGTTTGATACTCCACCTGAGGGGTTTAGTTTATCAG

************************************************************

Santhica TAACTTCTTAAAACTCTTATTATTTGGTGCATGAATTTATCCTTCATACCATAACATATA

PurpleKush TAACTTCTTAAAACTCTTATTATTTGGTGCATGAATTTATCCTTCATACCATAACATATA

************************************************************

Santhica ACATCACACTAATTTGATGGGAGCTATCTATCTTTCCATAACATGTACCAATCGTACAGT

PurpleKush ACATCACACTAATTTGATGGGAGCTATCTATCTTTCCATAACATGTACCAATCGTACAGT

************************************************************

Santhica ACTGAACTATTATATCTGTTTCTTTCTTTTTCATTAAATATGACATAATAGCCAAACAAA

PurpleKush ACTGAACTATTATATCTGTTTCTTTCTTTTTCATTAAATATGACATAATAGCCAAACAAA

************************************************************

Santhica AAAGTGCATGCATGACATAAAACTTAATTACGCAAATTAAATTATTTTCTCTCTATAAAT

PurpleKush AAAGTGCATGCATGACATAAAATTTAATTACGCAAATTAAATTATTTTCTCTCTATAAAT

********************** *************************************

Santhica ATATAGTTAATAATAATTAAAATATAAAGTATGTAGTACTGTACCACCTAATTATTGATT

PurpleKush ATATAGTTAATAATAATTAAAATATAAAGTATGTAGTACTGTACCACCTAATTATTGATT

************************************************************

Santhica GATAGTACTATTCTCTACCTTATTATTTAACACTTATAAAATCCTCACTTCAATCCCAAA

PurpleKush GATAGTACTATTCTCTACCTTATTATTTAACACTTATAAAATCCTCACTTCAATCCCAAA

************************************************************

Santhica CCAAAGCACCAAACACAACTCCTCTAAAAAAAACACCAATATATTGAATCTAAACATAAC

PurpleKush CCAAAGCACCAAACACAACTCCTCTAAAAAAAACACCAATATATTGAATCTAAACATAAC

************************************************************

Santhica AACACAATAGCCATAATAATCATCATGAATACCAAACACAAATCGAATTCCCGCGGCCGC

PurpleKush AACACAATAGCCATAATAATCATC------------------------------------

************************

Santhica CATGGCGGCCGGGAGCATGCGACGTCGGGCCCAATTCGCCCTATAGTGAGTCGTATTACA

PurpleKush ------------------------------------------------------------

Santhica ATCACTG

PurpleKush -------
